# Relative Burden of Large CNVs on a Range of Neurodevelopmental Phenotypes

**DOI:** 10.1371/journal.pgen.1002334

**Published:** 2011-11-10

**Authors:** Santhosh Girirajan, Zoran Brkanac, Bradley P. Coe, Carl Baker, Laura Vives, Tiffany H. Vu, Neil Shafer, Raphael Bernier, Giovanni B. Ferrero, Margherita Silengo, Stephen T. Warren, Carlos S. Moreno, Marco Fichera, Corrado Romano, Wendy H. Raskind, Evan E. Eichler

**Affiliations:** 1 Department of Genome Sciences, University of Washington School of Medicine, Seattle, Washington, United States of America; 2 Department of Psychiatry and Behavioral Sciences, University of Washington School of Medicine, Seattle, Washington, United States of America; 3 Department of Pediatrics, University of Torino, Turin, Italy; 4 Departments of Human Genetics, Biochemistry, and Pediatrics, Emory University School of Medicine, Atlanta, Georgia, United States of America; 5 Department of Pathology and Laboratory Medicine, Emory University School of Medicine, Atlanta, Georgia, United States of America; 6 IRCCS Associazione Oasi Maria Santissima, Troina, Italy; 7 Department of Medicine, Division of Medical Genetics, University of Washington School of Medicine, Seattle, Washington, United States of America; 8 Howard Hughes Medical Institute, University of Washington School of Medicine, Seattle, Washington, United States of America; University of Minnesota, United States of America

## Abstract

While numerous studies have implicated copy number variants (CNVs) in a range of neurological phenotypes, the impact relative to disease severity has been difficult to ascertain due to small sample sizes, lack of phenotypic details, and heterogeneity in platforms used for discovery. Using a customized microarray enriched for genomic hotspots, we assayed for large CNVs among 1,227 individuals with various neurological deficits including dyslexia (376), sporadic autism (350), and intellectual disability (ID) (501), as well as 337 controls. We show that the frequency of large CNVs (>1 Mbp) is significantly greater for ID–associated phenotypes compared to autism (p = 9.58×10^−11^, odds ratio = 4.59), dyslexia (p = 3.81×10^−18^, odds ratio = 14.45), or controls (p = 2.75×10^−17^, odds ratio = 13.71). There is a striking difference in the frequency of rare CNVs (>50 kbp) in autism (10%, p = 2.4×10^−6^, odds ratio = 6) or ID (16%, p = 3.55×10^−12^, odds ratio = 10) compared to dyslexia (2%) with essentially no difference in large CNV burden among dyslexia patients compared to controls. Rare CNVs were more likely to arise *de novo* (64%) in ID when compared to autism (40%) or dyslexia (0%). We observed a significantly increased large CNV burden in individuals with ID and multiple congenital anomalies (MCA) compared to ID alone (p = 0.001, odds ratio = 2.54). Our data suggest that large CNV burden positively correlates with the severity of childhood disability: ID with MCA being most severely affected and dyslexics being indistinguishable from controls. When autism without ID was considered separately, the increase in CNV burden was modest compared to controls (p = 0.07, odds ratio = 2.33).

## Author Summary

Deletions and duplications, termed copy number variants (CNVs), have been implicated in a variety of neurodevelopmental disorders including intellectual disability (ID), autism, and schizophrenia. Our understanding of the relevance of large, rare CNVs in a range of neurodevelopmental phenotypes, varying in severity and prevalence, has been difficult because these studies were restricted to the analysis of one disorder at a time using different CNV detection platforms, insufficient sample sizes, and a lack of detailed clinical information. We tested 1,227 individuals with different neurological diseases including dyslexia, autism, and ID using the same CNV detection platform. We observed striking differences in CNV burden and inheritance characteristics among these cohorts and show that ID is the primary correlate of large CNV burden. This correlation is well illustrated by a comparison of autism patients with and without ID—where the latter show only modest increases in large CNV burden compared to controls. We also find significant depletion in the frequency of large CNVs in dyslexia compared to the other cohorts. Further studies on larger sets of individuals using high-resolution arrays and next-generation sequencing are warranted for a detailed understanding of the relative contribution of genetic variants to neurodevelopmental disorders.

## Introduction

Recent studies have implicated large, rare CNVs in a range of neurodevelopmental disorders including intellectual disability (ID) [1], [2], autism [3], [4], schizophrenia [5], [6], bipolar disorder [7], [8], epilepsy [9], [10], and attention deficit hyperactivity disorder (ADHD) [11], [12]. Several themes have emerged from these studies: first, a significant enrichment for rare CNVs in individuals with the disease compared to unaffected controls was observed, independently, for each of these disorders; second, the same recurrent CNVs are associated with different neuropsychiatric phenotypes; and third, locus heterogeneity is substantial as many distinct variants can lead to similar phenotypes.

Our understanding of the relevance of rare CNVs across a broad spectrum of neurodevelopmental disorders, varying in severity and prevalence, is limited as previous studies were restricted to the analysis of one phenotype at a time and each of such studies was performed using different CNV genotyping methodologies with distinct platform-specific biases, making comparisons difficult. We undertook a systematic analysis of 1,227 cases and 337 controls to assess the relative contribution of CNVs in three phenotypically distinct neurodevelopmental disorders. We designed a whole-genome custom microarray targeted to genomic hotspots for comparative genomic hybridization (CGH) to identify potentially pathogenic CNVs that contribute to ID, autism, and dyslexia.

## Results

We analyzed 1,227 individuals ascertained for three neurodevelopmental disorders: 376 dyslexic children with a verbal IQ (VIQ) ≥90 on the Wechsler Intelligence Scale for Children [13] and dyslexia defined as poor performance and IQ-performance discrepancy in one or more of a set of standardized reading measures, 350 cases with sporadic autism from the Simons Simplex Collection (SSC), and 501 cases with ID. We used 337 NIMH control individuals for comparison. Further, based on the presence or absence of ID (full-scale IQ score cutoff of 70), autism cases were divided into those with ID (n = 97) or without ID (n = 253) (see Materials and Methods). Based on the presence of multiple congenital anomalies (MCA), individuals with ID were divided into those with ID only—i.e. idiopathic ID (n = 428)—and those with ID and MCA (n = 73).

All copy number variation analyses were performed using a custom microarray with a high probe density (∼2.6 kbp) targeted to 107 genomic hotspot regions [14] (∼251 Mbp) and a median probe spacing of ∼36 kbp in the genomic backbone (see Materials and Methods, Table S1). We used a Hidden Markov Model (HMM)-based algorithm to identify deletions and duplications. We restricted our analysis to CNVs >50 kbp in size to reduce false positive calls and validated all relevant CNVs using a second custom designed high-density array. To empirically determine the validation rate of the array at different genomic regions, we examined 118 CNVs detected in 24 samples and confirmed 117 events (>99% accuracy, see Table S2). While we were easily able to detect smaller events in the hotspot regions, the specificity of the array restricted our CNV discovery to >50 kbp in hotspot-associated regions and to >300 kbp in regions not associated with genomic hotspots (Figure S1).

### Analysis for large CNV burden in neurodevelopmental phenotypes

**Figure 1:**
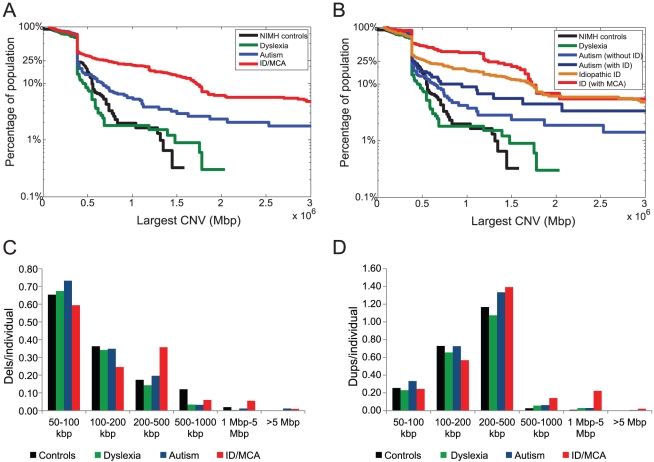
CNV burden in neurodevelopmental disorders. (A) The figure shows the population frequency of the largest CNV (as a survivor function) in individuals with ID, autism, dyslexia, and controls. (B) Population frequency of the largest CNV is shown for ID, ID with MCA, autism with ID, autism without ID, dyslexia, and NIMH control individuals. (C) Histograms depicting deletions per individual at each size range are shown. Note that 35 NIMH control samples carried an approximately 560 kbp deletion involving *PRAME* on distal 22q11.2. (D) Duplications per individual at each size range are shown. The hotspot chip has higher coverage over segmental duplication regions and therefore there is an expected abundance of duplications per individual compared to deletions.

After quality control (QC) filtering and manual curation, we obtained 5,086 CNVs in 1,395 out of 1,564 individuals (89.2%) with high-quality array CGH data (Table 1; Datasets S1, S2, S3, S4). Using these data, we compared the CNV enrichment between the multiple cohorts tested. We found a significant excess of large CNVs (>1 Mbp) in individuals with ID (p = 2.75×10^−17^, odds ratio = 13.71) or autism (p = 0.012, odds ratio = 2.99) when compared to controls analyzed on the same microarray platform (Figure 1). The frequency of large CNVs among children with dyslexia was similar to controls (p = 0.64, odds ratio = 0.94), although this might indicate a lack of statistical power in our study to detect any subtle enrichment (power >0.8 to detect 4.2% increase in burden) for large CNVs in dyslexia.

**Table 1:** Summary of disease cohorts and CNV analysis.

All	Total analyzed	Passed QC	Total CNVs	Average CNV size (bp)	Proportion of deletions	Proportion of CNVs disrupting genes	Average gene density
Controls	337	306	1,074	229,701	0.38	0.33	3.70
Dyslexia	376	322	1,041	217,135	0.37	0.34	3.82
Autism (no ID)	253	246	923	249,996	0.33	0.36	4.15
Autism (with ID)	97	90	362	342,637	0.41	0.33	4.95
Combined Autism	350	336	1,285	276,094	0.35	0.35	4.55
ID	428	358	1,306	442,519	0.33	0.38	6.12
ID/MCA	73	73	380	637,004	0.36	0.39	8.20
Combined ID cohort	501	431	1,686	486,353	0.34	0.38	7.16

For breaking genes, one or both of the CNV breakpoints should traverse a gene. CNV: Copy Number Variant; ID: Intellectual Disability; MCA: Multiple Congenital Anomalies; QC: Quality Control.

Within the neurodevelopmental disorder cohorts, a comparison showed a significantly greater large CNV burden in individuals with ID compared to autism (p = 9.58×10^−11^, odds ratio = 4.58) or dyslexia (p = 3.81×10^−18^, odds ratio = 14.45). When we partitioned the ID cohort into subsets with and without MCA, we observed a significantly increased large CNV burden in individuals with ID/MCA compared to ID alone (p = 0.001, odds ratio = 2.54). This trend was also observed when individuals with autism were separated into those with ID and without ID (Figure 1), although not statistically significant (p = 0.102, odds ratio = 2.1). When compared to controls, we noted a trend for increase in large CNV burden for autism without ID (p = 0.07, odds ratio = 2.33) as well as autism with ID (p = 0.0048, odds ratio = 4.85). In addition, a gene-based analysis showed an incremental increase in the proportion of disrupted genes and average gene density per CNV with higher estimates for the ID/MCA cohort as compared to ID alone or autism (Table 1). We also note that within the cohorts no bias towards deletions or duplications was observed in relation to phenotypic severity or variability (Tables S3, S4, S5, S6, S7). Overall, our results suggest a positive correlation of the severity of the phenotype to the size and gene density of CNVs.

### Rare CNVs in dyslexia, autism, and intellectual disability phenotypes

**Figure 2:**
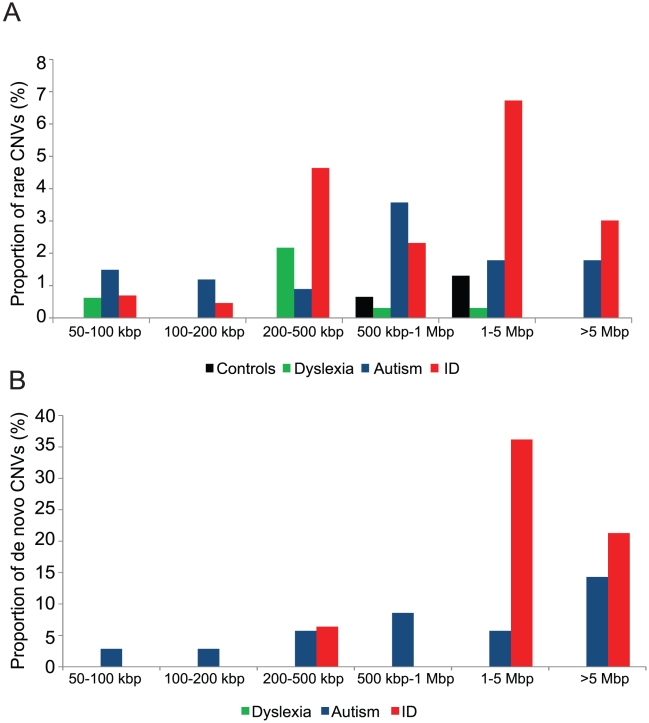
Rare CNVs and *de novo* rates in neurodevelopmental disorders. (A) The proportion of rare CNVs as a function of size is shown for NIMH controls and dyslexia, autism, and ID cohorts. To identify rare CNVs, we compared the pattern of CNVs from each of these cohorts to the CNV frequency map from 8,329 controls genotyped on Illumina arrays. (B) The proportion of *de novo* occurrence of CNVs among the three cohorts is shown for each size range. Note that the CNVs from the dyslexia cohort are all inherited. DNA from parents of NIMH controls was not available and hence not tested for *de novo* CNV frequency.

To identify rare CNVs of likely pathogenic significance, we compared the pattern of CNVs from dyslexia, autism, ID, and NIMH control cohorts to a map developed from an expanded set of 8,329 normal individuals genotyped with Illumina microarrays and to the publicly available Database of Genomic Variants [15] (see Materials and Methods). We eliminated common copy number polymorphisms and CNVs from our cases if they had a reciprocal overlap of 50% or more of their length with CNVs found in these 8,329 controls. After filtering, we compared the groups. We found a significant increase of rare CNVs in individuals with autism (35/336, 10%; p = 2.4×10^−6^, odds ratio = 6) or ID (69/431, 16%; p = 3.55×10^−12^, odds ratio = 10) compared to individuals with dyslexia (6/322, 2%) (Figure 2A, Table 2). In fact, when analyzed separately, the frequency of rare CNVs in NIMH controls (6/306, 2%) was not different compared to dyslexia (p = 0.57, odds ratio = 0.94) (Table S8).

**Table 2:** Rare CNVs in neurodevelopmental disorders.

Cohort	Total individuals analyzed	Number of individuals with rare CNVs	Total rare CNVs	Number of individuals with two rare CNVs	RefSeq genes	Median size of CNV	Hotspot CNVs	Genomic disorder CNVs
Dyslexia	322	6 (1.9%)	6 (1.9%)	0 (0%)	10	302 kbp	3 (50.0%)	0
Autism (with ID)	90	10 (11.1%)	11 (12.2%)	1 (10%)	445	1.62 Mbp	6 (54.5%)	5
Autism (no ID)	246	25 (10.2%)	25 (10.2%)	0 (0%)	235	633 kbp	15 (60.0%)	3
Combined Autism	336	35 (10.4%)	36 (10.7%)	1 (2.9%)	680	662 kbp	21 (58.3%)	8
Idiopathic ID	358	60 (16.8%)	64 (17.9%)	4 (6.7%)	1537	849 kbp	20 (31.3%)	15
ID (with MCA)	73	9 (12.3%)	13 (17.8%)	4 (44.4%)	678	1.86 Mbp	5 (38.5%)	5
Combined ID cohort	431	69 (16.0%)	77 (17.9%)	8 (11.6%)	2215	1.5 Mbp	25 (32.5%)	20

CNV: Copy Number Variant; ID: Intellectual Disability; MCA: Multiple Congenital Anomalies.

Given the high population prevalence of dyslexia [16], we then relaxed our selection to include events present at an allele frequency of <0.1% in controls (8/8,635) and identified four additional CNVs—i.e., a total of 10 CNV events (Table 3). The analysis of hotspot regions identified only one individual with dyslexia who carried a 15q11.2 BP1–BP2 deletion, which has previously been associated with ID [17], schizophrenia [18], [19], and epilepsy [20]; however, this deletion was also observed in 25/8,635 of our total control individuals. None of the seven deletions and three duplications detected in our dyslexia cohort mapped to candidate loci known to be associated with dyslexia [21].

**Table 3:** List of rare CNVs identified in neurodevelopmental disorders.

Chr	Start	End	Size	Chr. Band	CNV	Sample	Cohort	Type	Gene count	Inheritance	Control count	DECIPHER count	DGV count
chr14	21533898	22239332	705434	14q11.2	deletion	Si238	Autism_ID	non HS	12	maternal	0	2	6
chr12	15036168	29977743	14941575	12p12.3p11.2	deletion	Si159	Autism_ID	non HS	3	de novo	0	0	0
chr15	66881730	71972563	5090833	15q23q24.1	deletion	Si169	Autism_ID	HS assoc	2	de novo	0	0	0
chr3	59681529	79199503	19517974	3p14.2p12.3	deletion	Si163	Autism_ID	non HS	3	de novo	0	0	0
chr5	175479593	175584441	104848	5q35.2	deletion	Si118	Autism_ID	HS	1	paternal	0	0	4
chr7	31583983	31702682	118699	7p15.3	duplication	Si309	Autism_ID	HS	2	maternal	0	0	2
chr16	21645311	22520339	875028	16p12.1	duplication	Si247	Autism_No ID	HS	3	NA	0	2	0
chr22	38790464	39138992	348528	22q13.1	deletion	Si126	Autism_No ID	non HS	4	de novo	0	1	0
chr3	67276200	72402720	5126520	3p14.1p13	deletion	Si140	Autism_No ID	non HS	2	de novo	0	1	1
chr1	145303997	145357746	53749	1q21.1	homozyg deletion	Si192	Autism_No ID	HS	0	both	0	0	0
chr1	145303997	145357746	53749	1q21.1	deletion	Si85	Autism_No ID	HS	6	paternal	0	0	0
chr11	21703096	26791696	5088600	11p14.5	duplication	Si45	Autism_No ID	non HS	0	maternal	0	0	0
chr16	72859686	72917454	57768	16p22.3	deletion	Si99	Autism_No ID	HS	5	maternal	0	0	0
chr17	15301836	16542913	1241077	17p11.2	duplication	Si153	Autism_No ID	HS	147	paternal	0	0	0
chr17	32250000	32400000	150000	17q12	duplication	Si114	Autism_No ID	HS	1	paternal	0	0	0
chr17	42115600	42437714	322114	17q21.32	duplication	Si186	Autism_No ID	HS	53	de novo	0	0	0
chr17	58889277	59561178	671901	17q23.3	duplication	Si87	Autism_No ID	non HS	1	maternal	0	0	0
chr18	69039514	69822140	782626	18q22.3	deletion	Si173	Autism_No ID	non HS	2	maternal	0	0	0
chr19	14760644	15064029	303385	19p13.12	deletion	Si125	Autism_No ID	non HS	5	maternal	0	0	0
chr2	95159338	95228560	69222	2q11.1	duplication	Si226	Autism_No ID	HS	1	de novo	0	0	0
chr20	12657983	13311383	653400	20p12.1	deletion	Si20	Autism_No ID	non HS	17	paternal	0	0	0
chr22	18562002	18748556	186554	22q11.21	duplication	Si207	Autism_No ID	HS	5	de novo	0	0	0
chr3	84868129	85439477	571348	3p12.1	duplication	Si128	Autism_No ID	non HS	73	paternal	0	0	0
chr5	370492	1003781	633289	5p15.33	duplication	Si82	Autism_No ID	HS	17	paternal	0	0	0
chr5	175504664	175584441	79777	5q35.2	deletion	Si191	Autism_No ID	HS	3	maternal	0	0	4
chr6	51096930	51899775	802845	6p12.2	duplication	Si142	Autism_No ID	non HS	4	paternal	0	0	0
chr7	152102637	153356944	1254307	7q36.2	duplication	Si119	Autism_No ID	HS	123	paternal	0	0	0
chr7	153451569	154285634	834065	7q36.2	deletion	Si132	Autism_No ID	non HS	0	maternal	0	0	0
chr1	144106777	144451305	344528	1q21.1	deletion	2602	Dyslexia	HS	1	NA	2	3	0
chr3	139732796	140171095	438299	3q22.3	duplication	1806	Dyslexia	non HS	142	NA	1	1	0
chr6	65179840	66364033	1184193	6q12	deletion	2803	Dyslexia	non HS	1	paternal	1	1	0
chr4	123017758	123458923	441165	4q27	duplication	2286	Dyslexia	non HS	0	maternal	0	0	0
chr7	40606348	40819984	213636	7p14.1	deletion	2244	Dyslexia	non HS	1	maternal	0	0	0
chr7	68820751	68904999	84248	7q11.22	deletion	2867	Dyslexia	HS	2	paternal	0	0	0
chr7	69876932	70546042	669110	7q11.22	duplication	1102	Dyslexia	HS	1	paternal	0	0	0
chr7	110381300	110851860	470560	7q31.1	deletion	3437	Dyslexia	non HS	102	paternal	2	0	0
chr8	11373083	11434911	61828	8p23.1	deletion	1012	Dyslexia	HS	16	maternal	0	0	0
chr9	6348644	6740836	392192	9p24.1	deletion	1004	Dyslexia	non HS	11	paternal	0	0	0
chr9	1	9098781	9098780	9p24	deletion	3381	ID	non HS	3	de novo	0	15	0
chr11	121813520	134447248	12633728	11q24.1-q25	deletion	2597	ID	non HS	33	de novo	0	15	0
chr18	1	15313807	15313806	18p11.21	duplication	2492	ID	non HS	1	de novo	0	4	0
chr9	73827781	79830447	6002666	9q21.13	deletion	3413	ID	non HS	1	de novo	0	4	0
chr3	196825112	197208742	383630	3q29	deletion	3331	ID	HS	0	NA	0	2	1
chr6	161747330	162612669	865339	6q26	deletion	2562	ID	non HS	0	maternal	0	2	0
chr6	162129914	162555946	426032	6q26	deletion	2548	ID	non HS	48	paternal	0	2	0
chr9	218822	3742630	3523808	9p24	deletion	2615	ID	non HS	1	de novo	0	2	0
chr3	71242809	77832202	6589393	3p13	deletion	2509	ID	non HS	0	de novo	0	1	0
chr3	127000260	131353408	4353148	3q21.3	duplication	2237	ID	non HS	1	NA	0	1	0
chr6	107959196	111971187	4011991	6q21	deletion	2644	ID	non HS	0	de novo	0	1	0
chrX	146437800	147110597	672797	Xq27	duplication	2643	ID	non HS	6	NA	0	1	0
chrY	6895278	7233586	338308	Yp11.2	duplication	2580	ID	non HS	2	NA	0	1	0
chr1	76466419	77200494	734075	1p31.1	duplication	3399	ID	non HS	0	maternal	0	0	0
chr1	90483825	90786224	302399	1p22.2	duplication	1799	ID	non HS	48	NA	0	0	0
chr1	235537560	237086860	1549300	1q43	deletion	2518	ID	non HS	2	NA	0	0	0
chr10	128662416	129042087	379671	10q26.2	deletion	1402	ID	non HS	23	maternal	0	0	0
chr11	22232079	25091772	2859693	11p14.3	deletion	1613	ID	non HS	2	de novo	0	0	0
chr13	95576502	96051348	474846	13q31.3q32.2	deletion	2175	ID	non HS	3	NA	0	0	0
chr14	40428504	40755943	327439	14q21.1	duplication	3322	ID	non HS	89	NA	0	0	1
chr15	80767738	100147041	19379303	15q25	duplication	2522	ID	non HS	36	paternal or de novo	0	0	0
chr17	34089604	34566438	476834	17q12	duplication	72	ID	HS	4	NA	0	0	0
chr18	50965716	52820402	1854686	18q21	duplication	1164	ID	non HS	37	de novo	0	0	0
chr19	60032498	61147051	1114553	19q13.42	deletion	3262	ID	non HS	24	de novo	0	0	0
chr2	153753287	183588035	29834748	2q24.3q32.1	duplication	2559	ID	non HS	5	de novo	0	0	0
chr2	188179827	188853079	673252	2q32.1	duplication	2522	ID	non HS	1	paternal or de novo	0	0	0
chr3	50846910	58424157	7577247	3p21.31p14.3	duplication	3448	ID	non HS	54	de novo	0	0	0
chr3	62489781	63320060	832020	3p14.2	duplication	3445	ID	non HS	413	paternal	0	0	0
chr3	137464270	137768886	304616	3q22.3	deletion	3349	ID	non HS	25	NA	0	0	0
chr4	7148184	7818626	670442	4p16.1	duplication	2488	ID	non HS	2	NA	0	0	0
chr4	43534966	45590689	2055723	4p13	deletion	1318	ID	non HS	0	paternal	0	0	0
chr4	57346833	86106712	28759879	4q12q21.33	duplication	2154	ID	non HS	2	de novo	0	0	0
chr4	152300259	152723977	423718	4q31.3	deletion	699	ID	non HS	9	de novo	0	0	0
chr5	28427525	28630668	203143	5p14.1	deletion	2569	ID	non HS	4	NA	0	0	0
chr5	98793016	98851760	58744	5q21.1	deletion	1519	ID	HS	1	NA	0	0	0
chr5	156526018	164133824	7607806	5q33.3q34	duplication	3448	ID	non HS	1	de novo	0	0	0
chr6	92767349	92988105	220756	6q16.1	deletion	3316	ID	non HS	15	NA	0	0	0
chr7	45180992	45274014	93022	7p13	deletion	3296	ID	HS	1	paternal or de novo	0	0	1
chr7	51216627	51313401	96774	7p12.1	deletion	2571	ID	non HS	24	NA	0	0	0
chr7	68713709	69068502	354793	7q11.22	deletion	2433	ID	HS	66	NA	0	0	0
chr7	72300576	72486542	185966	7q11.23	duplication	2424	ID	HS	47	maternal	0	0	0
chr7	89028789	89548951	520162	7q21.13	duplication	3352	ID	non HS	42	NA	0	0	0
chr8	123404368	123637074	232706	8q24.13	deletion	3247	ID	non HS	0	de novo	0	0	0
chr9	21080948	21484861	403913	9p21.3	deletion	2462	ID	non HS	5	NA	0	0	0
chr9	38634661	38791196	156535	9p13.1	deletion	3236	ID	HS	0	paternal	0	0	0
chrX	28400903	28659988	259085	Xp21.3	duplication	3321	ID	non HS	0	NA	0	0	0
chrX	65611216	65934000	322784	Xq12	deletion	2597	ID	non HS	2	NA	0	0	2
chrX	88508702	91291323	2782621	Xq21.31q31.32	deletion	2511	ID	non HS	4	NA	0	0	0
chrY	3072083	6154525	3082442	Yp11.2	duplication	699	ID	HS	2	de novo	0	0	0
chr22	40103633	41458051	1354418	22q13.2	deletion	GB43	ID/MCA	non HS	2	NA	0	2	0
chr4	110560125	113895249	3335124	4q25	deletion	GB6	ID/MCA	non HS	15	de novo	0	1	0
chr1	174979260	238257861	63278601	1q24qter	duplication	GB88	ID/MCA	non HS	2	46,XX,t(1;5)(q23;p15) balanced	0	0	0
chr13	83679489	84385310	705821	13q31.1 dup	duplication	GB71	ID/MCA	non HS	9	maternal	0	0	0
chr3	164241146	168622524	4381378	3q26.1	deletion	GB42	ID/MCA	non HS	11	maternal	0	0	0
chr5	90252	1630763	1540511	5p15.33	deletion	GB88	ID/MCA	HS	0	46,XX,t(1;5)(q23;p15) balanced	0	0	0
chr7	27294680	28799546	1504866	7p15.3	duplication	GB65	ID/MCA	non HS	23	de novo	0	0	0
chr9	201336	16672312	16470976	part trisomy 9	duplication	GB71	ID/MCA	HS assoc	1	46,XX rcp (8;10)(q2.2;q21.2)+t (9;12)(p2.2;p1.3)	0	0	0

This list does not contain known genomic disorders. Please refer to Table S9 for known genomic disorders identified in this study. CNV: Copy Number Variant; ID: Intellectual Disability; HS: Hotspot; MCA: Multiple Congenital Anomalies.

Analysis of 336 individuals from the SSC autism cohort showed that 35 individuals (10%) carried 36 rare CNVs (680 RefSeq genes, median size = 662 kbp) and about 58% (21/36) of these CNVs mapped to genomic hotspots (Table 2). Only eight of the events (all hotspot sites) associated with genomic disorders, including 22q11.2 deletion (*TBX1*, DiGeorge syndrome), 17p12 duplication (*PMP22*, Charcot-Marie-Tooth disease), and 15q11.2q13.1 duplication (*UBE3A* and *SNRPN*) (Table S9). In addition, as reported previously [3], [22], [23], the autism-associated proximal 16p11.2 deletion (*TBX6*) was observed in approximately 1% (3/336) of all autism cases analyzed. Interestingly, one case with a *de novo* 16p11.2 deletion also inherited a 2 Mbp duplication 22q11.2 (*TBX1*) from the mother.

Among 431 cases with ID (358 cases with ID only and 73 cases with ID plus MCA), 69 individuals carried 77 rare CNVs (2,215 RefSeq genes, median size = 1.5 Mbp) that were either of known pathogenic significance or not observed in a total set of 8,635 controls, and 32% (25/77, median size = 1.42 Mbp) of these variants localized to genomic hotspot regions (Table 2). This is a significant enrichment for rare CNVs in the ID cohort compared to autism (p = 0.019, odds ratio = 1.6) or dyslexia cohorts (p = 3.55×10^−12^, odds ratio = 10). Interestingly, 20/77 CNVs (16 hotspot and four non-hotspot sites) mapped to a known genomic disorder site, including those associated with variable phenotypes such as 15q13.1q13.3 (*CHRNA7*), 16p11.2 proximal (*TBX6*; two cases) and distal (*SH2B1*) hotspots, 16p13.11 (*MYH11*; three cases), 17q12 (*TCF2*), and 3q29 (*DLG1*) as well as syndromic regions such as 7q11.23 (Williams syndrome), 17q21.31 (*MAPT*), 5q35 (Sotos syndrome), 8p23.1, 22q13 (Phelan-McDermid syndrome) [24] and 1p36 [25].

We next sought to determine whether these rare CNVs were inherited or if they arose *de novo* in the probands. Parental DNA samples were available to investigate inheritance for 90 out of 123 rare CNVs detected in all three disease cohorts (Table S8). In four cases, only maternal DNA was available. We find that 44/90 CNVs arose *de novo* and a majority (77%, 34/44) of these *de novo* CNVs were large (>1 Mbp). Overall, we find a greater proportion of *de novo* events in ID (64%, 30/47) compared to autism (40%, 14/35; p = 0.027, odds ratio = 2.6) or dyslexia (0/8; p = 0.0009, odds ratio = infinity) (Figure 2B). These data are suggestive of a general trend of increased *de novo* rates and CNV size with increased severity of the disorder.

### Novel, rare CNVs reveal potential candidate genes

**Figure 3:**
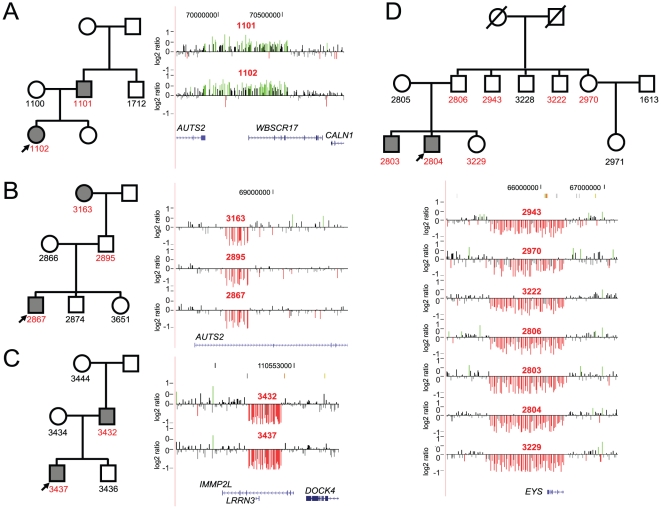
Pedigree shows the inheritance of a 669 kbp duplication encompassing *AUTS2* and *WBSCR17* from a father to the daughter. (A) The father has features of dyslexia with a verbal IQ (VIQ) of 122, WATT^a^ 93, WRAT3sp^b^ 83 and WIATsp^c^ 94. The daughter's scores are VIQ 122, WID^d^ 86, WATT 91, WRAT3sp 90, and WIATsp 94. (B) In this pedigree the 84 kbp deletion within *AUTS2* is transmitted to the proband (VIQ 122, WATT 97, WRAT3sp 96, WIATsp 94) from the affected paternal grandmother (VIQ 118, WATT 88) through the unaffected father. (C) A deletion within *IMMP2L* is shown for this family. The deletion is transmitted to the proband (VIQ 111, WID 83, WATT 82, WRAT3sp 85, WIATsp 81) from his affected father (VIQ 84, WRAT3sp 68, WIAT-2sp 66). Interestingly, *IMMP2L* variants have been associated with Tourette syndrome, ADHD, and autism. (D) A 1.2 Mbp deletion within *EYS* is shown in several family members of this large pedigree. While the proband (VIQ 107, WID 56, WATT 81, WRAT3sp 87, WIATsp 87) and his affected brother (VIQ 101, WID 66, WATT 82, WRAT3sp 78, WIATsp 85) carried the deletion, so did many other unaffected relatives, including the father. Although no inference can be drawn for its role in dyslexia, as the deletion does not segregate with the phenotype, recessive mutations in *EYS* have been associated with retinitis pigmentosa. ^a^WATT- WRMT-R Woodcock Reading Mastery Test – Revised; Word Attack subtest [67]. A measure of untimed reading of single non-words. ^b^WRAT3sp - Wide Range Achievement Tests – Third Addition; Spelling subtest [68]. Spelling of single words from dictation in writing. ^c^WIAT(2)sp - Wechsler Individual Achievement Test (2^nd^ edition); Spelling subtest [69]. Spelling of single words from dictation in writing. ^d^WID - WRMT-R Woodcock Reading Mastery Test – Revised; Word Identification subtest [67]. A measure of untimed reading of single words.

We then focused on rare CNVs involving single genes or regions of potential interest. In the dyslexia cohort, two unrelated families carried CNVs on chromosome 7q11.23 that involved the autism susceptibility candidate 2 (*AUTS2*, MIM# 607270). A 669 kbp duplication that included *AUTS2* and *WBSCR17* was transmitted from an affected father to the daughter and an approximately 84 kbp deletion was transmitted from the affected paternal grandmother through the unaffected father to the proband (Figure 3). In addition, we also identified a 354 kbp deletion encompassing *AUTS2* in one individual with idiopathic ID, pervasive developmental delay, partial epilepsy, and left hemihypertrophy. An approximately 1.2 Mbp deletion encompassing the *eyes shut* drosophila homolog gene (*EYS*, MIM# 612424) on chromosome 6q12 was detected in an affected proband and several unaffected family members. Although autosomal recessive single-nucleotide mutations in *EYS* have been reported in patients with retinitis pigmentosa [26], [27], the role of heterozygous microdeletions involving this gene is unknown.

We also identified a 471 kbp deletion involving *IMMP2L* inherited by the proband from the affected mother (Figure 3). Deletions involving *IMMP2L* have been associated with ADHD [12], autism [28], and Tourette syndrome [29]. Recently, Pagnamenta and colleagues also reported a 594 kbp *IMMP2L-DOCK4* deletion resulting in a fusion transcript and an intragenic *DOCK4* deletion segregating with dyslexia [30]. Our results are best interpreted within the context of candidate gene identification in dyslexia. Although at least nine chromosomal loci are associated with dyslexia, for two of these loci the candidate genes were identified on the basis of a rare balanced chromosomal translocations disrupting *ROBO1* [31] and *DYX1C1*/*EKN1* [32], [33]. More recently, a Danish Cytogenetic Registry study of all cases with chromosomal translocations identified additional novel dyslexia candidate genes affirming the value of rare structural variants in understanding the genetics of dyslexia [34]. Our study is the first to systematically characterize rare CNVs in dyslexia and thus evaluate the contribution of rare deletions and duplications to this common genetic disorder.

**Figure 4:**
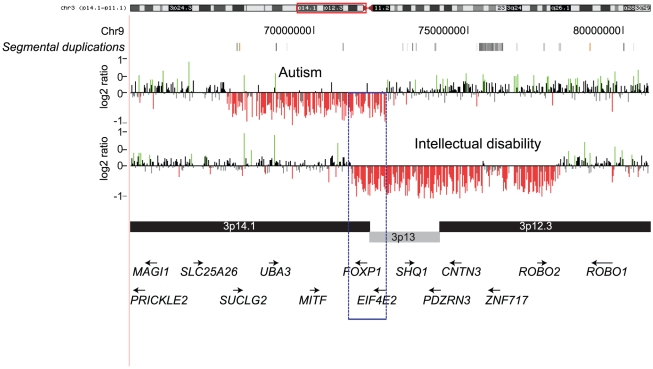
*FOXP1* deletions in individuals with autism and ID. Two deletions (5 Mbp and 6.6 Mbp) are shown intersecting at a common region of 1.16 Mbp containing *FOXP1*. Note that the deletion in the autism individual also covers *ROBO2* and *CNTN3*.

Within the autism cohort, several novel deletions and duplications involving neurologically-relevant genes were identified. A 5 Mbp *de novo* deletion involving *FOXP1* on chromosome 3p14.1 was identified in an individual with features of idiopathic autism (full-scale IQ = 75). An additional 6.6 Mbp *de novo* deletion overlapping *FOXP1* was also identified in an individual with idiopathic ID (Figure 4). A review of the DECIPHER database revealed a similar-sized deletion disrupting *FOXP1* in an individual with developmental delay, sensorineural deafness, hypotonia, club foot, and dislocation of hip. Recently, *FOXP1* was implicated in autism, ID, and language impairment [35], [36], [37]. It is believed that *FOXP1*interacts with *FOXP2* and *CNTNAP2*, both implicated in speech disorders and autism [38], [39]. The overlapping 1.16 Mbp region of the deletion common to both autism and ID indicates a potential involvement of *FOXP1* in pathways related to both of these disorders. Other variants involving functionally relevant genes include 7q36.2 deletion and duplication (*DPP6*), 17q23.3 duplication (*SCN4A*), and 17q21.32 duplication (*WNT3* and *WNT9B*).

**Figure 5:**
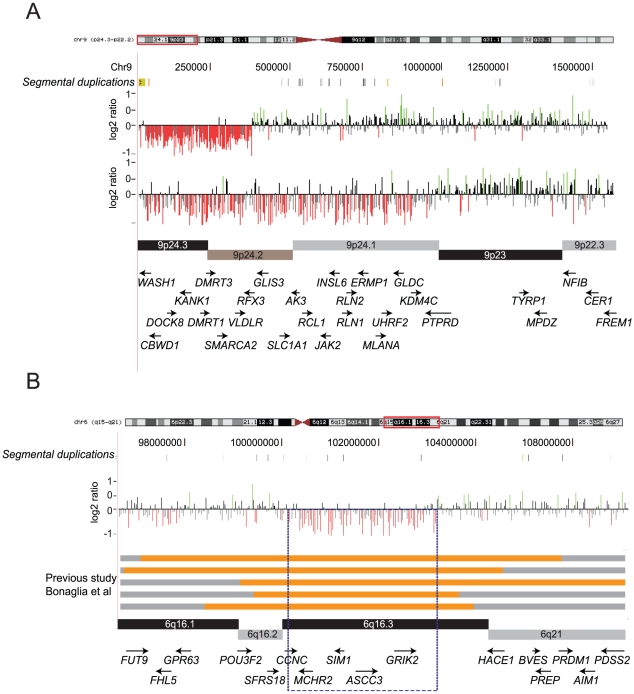
Novel CNVs identified in the ID cohort. (A) Overlapping deletions on chromosome 9p24 are shown. Deletions of this region containing *DMRT1* and *DMRT3* have also been associated with urogenital abnormalites and sex reversal. (B) A ∼3 Mbp deletion on 6q16 is shown encompassing *SIM1*. Larger deletions of this region were previously reported and have been associated with obesity. Orange bars denote deletions and gray regions are non-deleted regions from previous studies [41]. Taken together with other published studies, this deletion narrows down the critical region (dotted box) to about 2.9 Mbp.

Our analysis of the ID cohort was enriched for singleton events often involving genes related to developmental or neurological functions including *SYNPR*, *GABRA*, *AUTS2*, *FOXP1*, *FKBP6*, *COBL*, and *FMR1*. However, within the same cohort we were also able to detect novel overlapping deletions (3.5 Mbp and 9 Mbp) on chromosome 9p24 in two unrelated individuals (Figure 5A). Both cases exhibited clinical features of ID and Pervasive Developmental Delay-Not Otherwise Specified. The distal breakpoints of these deletions map to segmental duplications while the proximal end maps within a high density of repeat elements. A survey of this region in the DECIPHER database [40] revealed about 15 cases with overlapping deletions. Variable clinical presentations and heterogeneity of deletion breakpoints preclude further genotype-phenotype correlation studies for this region (Figure S2). We also identified a nonrecurrent 6q16 deletion (chr6: 100,383,567-103,310,184) that potentially narrows the critical region for this recently described Prader-Willi-like syndrome [41] to approximately 2.9 Mbp. The refined critical region contains only five genes including the obesity-associated *SIM1* [42] and the autism-associated *GRIK2* [43] (Figure 5B). About 70% of children with 6q16 deletion manifest obesity [41]; however, our case with the smaller deletion, encompassing *SIM1*, showed no evidence of obesity at 10 years of age (Table S10).

### Multiple large CNVs associate with phenotypic severity

**Figure 6:**
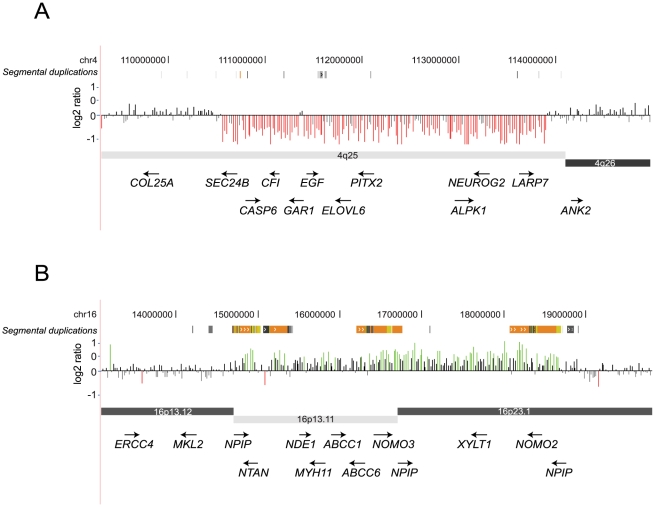
Two large CNV hits in a case with ID/MCA. A 3.3 Mbp deletion containing *PITX2* as well as the 3.4 Mbp paternally inherited 16p13.33 duplication is shown for an individual with ID plus MCA. This individual has features of Rieger syndrome including visual defects, mild hypotonia, right congenital glaucoma, left microophalmia, and anterior segment dysgenesis. Other features include cleft uvula, hypodontia and conical teeth, hyperplasia of frenulum of tongue, midface hypoplasia, strabismus, and deafness.

We find that 8/69 (11.6%) cases in the ID cohort carried more than one large, rare CNV and *all* of these individuals presented with severe clinical features (Tables S6, S7, S8). A striking difference (p = 0.008, odds ratio = 11.2) in multiple CNV rates was also observed when the ID cohort was divided into those with severe MCA (44%) and those with idiopathic ID (6.7%). Notable examples are co-occurrences of a 3.4 Mbp 16p13.11 duplication and a 3.3 Mbp deletion on chromosome 4q25 involving *PITX2* in a case with features of Rieger syndrome [44] (Figure 6) and a 17p13.3 deletion (*YWHAE*, Miller-Dieker syndrome) and 3q29 duplication (*DLG1*) in a child with cryptorchidism, ventricular septal defect, and seizures. The 3q29 duplication is a recurrent interstitial rearrangement [45], [46] potentially mediated by flanking segmental duplications of high sequence identity (27 kbp size, 96% identity). The 17p13.3 deletion is a previously reported nonrecurrent rearrangement associated with Miller-Dieker syndrome [47], [48]. In contrast, only 1/35 (2.9%) autism cases and none of dyslexia individuals carried another large CNV. This observation suggests that the severity of the phenotypes can be influenced by more than one large, rare CNV co-occurring in the same individual. During this analysis we considered the possibility of a derivative chromosome representing an unbalanced translocation possibly creating the impression of multiple CNVs in our cases. We carefully reviewed available chromosomal analysis data (G-banded karyotyping or FISH) for each of the individuals with two hits reported in our study. We did find one case with two hits where apparent CNVs represent a derivative chromosome inherited from a balanced translocation carrier parent (Table S8D).

## Discussion

Initial discoveries of significant enrichment of rare CNVs for ID and autism led to testing the CNV basis for other behavioral and neurodevelopmental disorders of varying population frequency and severity, such as schizophrenia, ADHD, epilepsy, bipolar disorder, and Tourette syndrome. However, comparisons between these studies have been difficult due to differences in study design, insufficient sample sizes, and lack of detailed phenotype information. In this study, we compared 1,564 individuals (cases and controls) on a single platform of relatively modest density with the same type of detection bias. We utilized the duplication architecture of the human genome to custom design a DNA oligonucleotide microarray enriched for genomic hotspots, i.e., regions flanked by high-identity segmental duplications. This array has an advantage over several other commercial arrays in that there is a 25-fold enrichment for recurrent events in the genomic hotspots compared to the rest of the genome [49]. Therefore, fewer samples are required to identify several unrelated individuals with the same pathogenic mutation. We find that our array has a comparable diagnostic yield of 16% for the ID cohort compared to other clinical chromosomal microarray studies reported in the literature (Figure S3).

In strong agreement with previous studies, our data suggest that multiple, rare CNVs contribute to the etiology of autism and ID. In contrast, we find no increase in large pathogenic CNVs in individuals with dyslexia compared to controls. Notwithstanding, our analysis revealed novel regions of potential relevance to the etiology of dyslexia. Two unrelated children (2/322, 0.6%) with dyslexia carried CNVs encompassing *AUTS2*, both inherited from a parent. While the phenotype of dyslexia segregated with the *AUTS2* duplication in the first family (Figure 3A), in the second family the deletion was inherited from affected grandmother through unaffected father (Figure 3B). This could be due to a phenomenon described as “compensation”, where some adults that reported difficulties with reading in childhood no longer evidence signs of dyslexia [50]. Previous studies of *de novo* chromosomal translocations and inversions identified breakpoints within *AUTS2* in individuals with autism and/or ID phenotypes [51], [52], [53], [54], [55]. More recently, unique *AUTS2* deletions and duplications were observed in Juvenile Myoclonic Epilepsy [10] and ADHD [11], [12]. It is interesting to note that ADHD and dyslexia are frequently comorbid and may have shared genetic risk factors [56], [57], [58]. When our study is taken together with recent CNV studies of ADHD [11], [12], *AUTS2* CNVs were observed in 5/2,306 combined cases and 3/46,947 unscreened controls (p = 1.12×10^−5^, odds ratio = 33.9), indicating that *AUTS2* might have an important role in pathways related to cognition. While the function of *AUTS2* is still unclear, it is strongly expressed in fetal and adult brains, particularly in the frontal, parietal, and temporal lobes [59]. Interestingly, *AUTS2* and the 7q11.2 region were identified as having the strongest statistical signal for positive selection in early modern humans as compared to the Neanderthal genome [60], indicating that *AUTS2* might be important for a specialized human function such as cognition.

The CNV profile we observed in individuals with dyslexia was essentially the same as that in control individuals. This is not surprising if we take into consideration that all the subjects in our dyslexia sample had a VIQ above the 25%ile and the mean VIQ of the cohort was 110 (2/3 standard deviations above the general population mean), and given that we have shown that the CNV profile correlates with the severity of ID. The genes involved in dyslexia are likely to affect more specialized cognitive functions, may not adversely affect general intelligence, and may be more amenable to discovery with high-density arrays capable of detecting single gene or single exon CNVs or SNP microarrays that can leverage SNP allele frequency information in addition to signal intensity. In addition, all hybridization-based platforms fail to detect copy number neutral changes, such as balanced chromosomal rearrangements and inversions. This is particularly germane to dyslexia where a large number of candidate genes have been identified through mapping of translocation breakpoints [21], [34].

A comparison of rare *de novo* CNV rates for autism shows that our estimates (4%, 14/336) fall within a range of 4–10% reported previously by other large-scale, high-density array studies [4], [61], [62], [63]. This suggests that no platform-specific bias exists for large variants and also that the contribution of large CNVs is consistent across all studies for autism. We find a significantly greater enrichment for large CNVs, higher *de novo* rates, and a higher frequency of two rare CNV hits in individuals with ID-associated phenotypes compared to autism or dyslexia. This observation is exemplified by the fact that individuals with autism with ID have more large CNVs than those with autism only. We also find a significant difference between individuals with autism versus those with dyslexia. Sanders and colleagues recently analyzed 1,124 SSC families affected with autism spectrum disorder (ASD). Using stepwise linear models, they evaluated the relationship between intellectual functioning, sex, and the number of genes within rare, *de novo* CNVs. While the number of genes affected correlated with the size of the *de novo* CNV, the authors did not find a strong correlation of the Autism Diagnostic Observation Schedule (ADOS) combined severity score (p = 0.25, R^2^ = 0.005) or full-scale IQ (p = 0.02, R^2^ = 0.08) with the size of the CNV. In contrast, we considered all large CNVs (common and rare, *de novo* and transmitted) identified in a relatively smaller sample size and essentially bifurcated the autism cohort using a full-scale IQ score cutoff of 70. There was also a greater enrichment of two hits in the ID cohort (11.6%) compared to the autism cohort (2.8%). In fact, one individual carrying a 16p11.2 deletion with autism and features of ID also has a maternally inherited 22q11.2 duplication (*TBX1*) providing further evidence for the two-hit hypothesis we previously proposed for severe developmental delay [64]. Further, the frequency of two hits was even more striking when only individuals with ID/MCA were considered (44%), albeit the number of cases is few. We believe these data provide support for an incremental effect of CNV size and number on the severity of phenotypic outcome.

Our experimental design is biased towards interrogating hotspot regions in the human genome. A comparison to recently reported studies [61], [63] suggests that the majority of false-negative calls will reside within non-hotspot regions due to a lack of probe coverage (<10 probes). While the detection power of our array increases with the size of the variant, we would certainly miss smaller and intragenic CNVs, for example in autism candidate genes such as *NRXN1* [65], [66], *CACNA1C*, *SLC4A10*, *MAGI1* [63], *SYNGAP1*, *DLGAP2* [62], *NLGN1*, *ASTN2* [67], and exonic copy number variants in *ASPM*, *DPP10*, *CNTNAP2*, *A2BP1*, *PCDH9* [68], and *PTCHD1* [69]. While we find no excess of large CNVs in dyslexia, there is still the possibility that large CNVs are relevant in some familial cases of the disease as well as occasional sporadic cases. Further studies are warranted for a more detailed analysis of all the three neurodevelopmental cohorts using high-resolution arrays and next-generation exome and/or whole-genome sequencing. While it can be difficult to compare data derived from different microarrays, there is value to multiple array platforms and cross-platform validation. The depositing of the resulting data into publicly available databases will facilitate the continued elucidation of recurring clinically significant CNV and genotype-phenotype correlations.

## Materials and Methods

### Ethics statement

Patients from each of study cohort were recruited after appropriate human subjects approval and informed consent. Informed consent was also obtained to publish photographs.

### Patient ascertainment based on severity of phenotypes

DNA samples were obtained from cases ascertained for three neurodevelopmental disorders of varying severity: (1) ID/developmental delay and MCA, (2) dyslexia or reading impairment, and (3) idiopathic autism. We defined severity of clinical features based on presence or absence of ID (IQ<70) for the autism group and congenital malformation for the ID group. Our dyslexia cohort had no ID or congenital malformation cases; as an IQ≤90 and the presence of congenital malformations were exclusion criteria. Individuals with idiopathic autism were partitioned into those with autism and ID (IQ<70) and those without ID (IQ>70). For the ID cohort, those individuals with brain malformations, gross craniofacial dysmorphology, cardiac defects, and neurological deficits were separated into an ID plus MCA (ID/MCA) group. Thus, in the order of severity, the ID/MCA cohort is considered the most severe, followed by ID only, autism with ID, autism without ID, dyslexia, and normal controls. However, we note that although the individuals with dyslexia do not have ID, they have severe impairments in core phonological measures leading to significantly reduced reading abilities despite normal IQ (IQ≥90). Detailed descriptions of each of the cohorts are given below.

### Ascertainment of individuals with dyslexia or reading disability

For the dyslexia subject set, children were considered eligible for the study if they met researcher-defined criteria based on test scores from a standardized battery of tests. DNA samples were obtained from two cohorts. The first cohort included probands aged 6 to 16 from 198 families who were initially ascertained at the University of Washington (UW) multidisciplinary Learning Disability Center (UWLDC) under protocols approved by the UW Institutional Review Board. For the UWLDC cohort, probands were required to have a prorated VIQ at or above 90 (≥25%ile) on the Wechsler Intelligence Scale for Children – 3^rd^ edition [13], with performance below the age-specific population mean and at least one standard deviation below the VIQ on one or more out of 10 research measures of reading, writing, or spelling. As a group, on average, probands met the impairment criteria between 6 and 7 measures. As expected by ascertainment requirements, the average VIQ of probands in this cohort was 110 (≥75%ile) [70], [71]. Siblings older than 6.5 years were invited to participate, and additional family members were added using a sequential sampling strategy to extend pedigrees through family members with the most extreme impairment values on the same 10 research measures. Detailed recruitment and evaluation procedures for the UWLDC cohort were described earlier [50], [72] (see Table S4A).

For the second cohort, 178 children aged 5 to 12 were recruited from a special K-6 school for students with dyslexia or via their direct relatives in the Atlanta area (The Schenck School, Atlanta, GA). For this cohort, children were required to have a psychological battery of tests completed by a licensed psychologist and usually have a diagnosis of a reading disability. Based on strong verbal comprehension score, perceptual reasoning score, Peabody picture vocabulary test, or other cognitive tests that measure intelligence, these children have average to above-average intelligence. Both cohorts were composed of individuals with >90% Caucasian ethnicity with an approximately equal number of males and females. Except for ADHD, children with other psychiatric and neurological disorders, moderate to severe receptive language disorders, developmental disabilities, or other conditions known to affect cognition were excluded based on parental questionnaire. Clinical details are shown in Table S4B.

### Ascertainment of individuals with features of autism with or without ID

For the autism cohort, families were identified through the SSC (www.sfari.org) [73]. The Simons Foundation-funded SSC includes families with no more than one child with autism ascertained through 12 data collection sites across North America. Of the 350 individuals included in this study 297 (85%) are of Caucasian ethnicity. Inclusion criteria in the collection requires that the child with autism meet ASD criteria on the ADOS [74], on the Autism Diagnostic Interview, Revised (ADI-R) [75], and meet expert clinical judgment. Nonverbal IQ estimate must also be greater than 35. Children with significant hearing, vision, or motor problems, significant birth complications (e.g. extended NICU stay), or with a diagnosis of ASD-related disorders, such as Fragile X, were excluded. Children with a relative (up to third degree) with ASD or sibling who showed ASD-related symptoms were also excluded. Diagnostic evaluations, cognitive assessment, and phenotypic characterization were conducted at each site with data collection, data entry, and data validation methods standardized across sites to ensure reliability of sample collection. We further partitioned the autism cohort into those associated with ID (average full scale IQ = 49) consisting of 97 cases (73 males, 24 females; median age, 12 years) and those without ID (average full scale IQ = 98.9) comprising 253 cases (228 males, 25 females; median age, 11 years and 11 months). Clinical details are shown in Table S5.

### Ascertainment of individuals with intellectual disability with or without congenital malformation

The idiopathic ID cohort was selected from individuals admitted to the IRCCS Associazione Oasi Maria Santissima and screened for ID according to the Diagnostic and Statistical Manual of Mental Disorders-IV-Text Revision (DSM-IV-TR) criteria. This cohort consists of 428 cases (153 females, 275 males; median age, 15 years) of Caucasian ethnicity with idiopathic ID and previously excluded for common causes of ID, including Fragile X syndrome, trisomies 21 and 13. In addition, classical genomic syndromes such as Smith-Magenis, DiGeorge, Prader-Willi/Angelman, and Williams syndromes, if recognized by clinical evaluation, were followed up for confirmation using targeted multiplex ligation-dependent probe amplification and excluded. We note that cases with phenotypic variability that escape clinical detection might not have been excluded. Typically, idiopathic cases of ID with no classical constellation of clinical features suggestive of a known disorder or those with mild to moderate ID without significant congenital malformation were included in this cohort. Clinical details are shown in Table S6.

Individuals with features of ID with MCA not necessarily assigned to a specific syndrome were evaluated and recruited at the University of Torino. This cohort consists of 73 individuals (32 females and 41 males) of Caucasian ethnicity with a median age of 2 years at diagnosis. Clinical features of these individuals included brain malformations, craniofacial dysmorphology, and neurological deficits along with variable ID (Table S7). Informed consent was obtained from all the subjects included in both the studies.

### Ascertainment of normal controls

The control cohort consisted of 337 DNA samples obtained from the Rutgers University Cell and DNA Repository (www.rucdr.org). These individuals were ascertained by the NIMH Genetics Initiative [76] through an online self-report based on the Composite International Diagnostic Instrument Short-Form (CIDI-SF) [77] and screened specifically for eight mental health disorders, including major depression, bipolar disorder, and psychosis, but were not screened for dyslexia and therefore not ideal for such comparisons. Those who did not meet DSM-IV criteria for major depression, denied a history of bipolar disorder or psychosis, and reported exclusively European origins were included [78], [79].

Additionally, CNV data from 8,329 additional cell line and blood-derived controls were used to assess the frequency of our putative pathogenic CNVs in a larger population of neurologically normal individuals. These data were derived primarily from genome-wide association studies of non-neurological phenotypes. Although these data were not ascertained specifically for neurological disorders, they consist of adult individuals providing informed consent. Specifically, datasets from the following sources were included in our analysis: Human Genome Diversity Project [49], [80]; National Institute of Neurological Disorders and Stroke (NINDS) (dbGaP accession no. phs000089) [49], [81]; Pharmacogenomics and Risk of Cardiovascular Disease (PARC/PARC2) [82], [83]; parents of asthmatic children courtesy of Stephanie London [49]; Fred Hutchinson Cancer Research Center (prerelease data provided courtesy of Aaron Aragaki, Charles Kooperberg, and Rebecca Jackson as part of an ongoing genome-wide association study to identify genetic components of hip fracture in the Women's Health Initiative); InCHIANTI (data provided by InCHIANTI study of aging, www.inchiantistudy.net) [49], [84]; and the Wellcome Trust Case Control Consortium phase 2 (National Blood Service) [7]. All samples were genotyped on Illumina arrays using methodology described previously [49] [85] and either natively processed in hg18 or re-mapped after CNV calling (NINDS and PARC) to hg18 using the UCSC LiftOver tool (http://genome.ucsc.edu).

### Array CGH and analysis

We designed custom targeted hotspot v1.0 arrays comprised of 135,000 probes (by Roche NimbleGen) with higher density probe coverage (median probe spacing 2.6 kbp) in the genomic hotspots (regions flanked by segmental duplications) and a lower probe density in the genomic backbone (median probe spacing 36 kbp). All microarray hybridization experiments were performed as described previously [86], using a single unaffected male (GM15724 from Coriell) as reference. All validation experiments were performed using two custom array designs: (1) a custom targeted 4×180 K Agilent chip with median probe spacing of 2 kbp in the genomic hotspots and whole-genome backbone coverage of one probe every 36 kbp (Agilent Technologies) and (2) a custom targeted 3×720 K NimbleGen or 2×400 K Agilent chip with median probe spacing of 500 bp in the genomic hotspots and probe spacing of 14 kbp in the genomic backbone.

All arrays were analyzed by mapping probe coordinates to the human genome assembly Build 36 (hg18). Using chromosome-specific means and standard deviations, normalized log intensity ratios for each sample were transformed into z-scores. These z-scores were then classified as “increased”, “normal”, or “decreased” in copy number using a three-state HMM. The HMM was applied using HMMSeg [87]. For each sample, HMM state assignments of probes were merged into segments if consecutive probes of the same state less than 50 kbp apart. If two segments of the same state were separated by an intervening sequence of ≤5 probes and ≤10 kbp, both segments and intervening sequence were called as a single variant. Further, we employed stringent QC measures and empirically estimated post-HMM filtering thresholds (absolute z-score >1.5 and >10 probes) to increase the specificity of our experiments. With these filtering criteria, we were able to thoroughly scan HMM outputs for CNV events and manually check the validity of each call by examining the normalized log intensity ratios across a chromosome. For the Agilent arrays, data analysis was performed following feature extraction using DNA analytics with ADM-2 setting according to the manufacturer's instructions. All CNVs calls were visually inspected in the UCSC genome browser.

First, we carried out validation on 24 samples from the developmental delay cohort and confirmed 117/118 HMM-inferred calls with a validation rate of 99.15%. Next, we validated 84 CNVs from an independent set of cases both validated using fluorescence *in situ* hybridization (FISH) and locus-specific custom high-density arrays [64] (Girirajan and Eichler, unpublished). We also validated all 44 calls from the autism cohort, 22 calls from the dyslexia cohort, and 78 calls from the developmental delay cohort. In addition, in an analysis of 517 individuals with epilepsy using this array design, 61/63 CNVs were validated on a different array platform [10].

## Supporting Information

Dataset S1 CNV calls in autism.
(XLSX)

Dataset S2 CNV calls in ID.
(XLSX)

Dataset S3 CNV calls in NIMH controls.
(XLSX)

Dataset S4 CNV calls in dyslexia.
(XLSX)

Figure S1 Probe densities and CNV detection threshold of hotspot v1 chip. Although the median density of the chip was designed to be approximately 2.6 kbp in the genomic hotspots and 36 kbp in genomic backbone, the limitations of the chip design (probe assignment restricted to only up to five mismatches) precluded uniform distribution of the probes throughout the genome. Therefore, the actual probe density varied across regions of the human genome. (a) The plot shows the size-wise distribution of CNVs and the density of array probes targeted to the genomic hotspots and non-hotspot regions. Note that non-hotspot regions contain two different probe densities (probe spacing of 20,000 bp and about 30,000 bp labeled *a* & *b*) and the hotspots (shaded) are covered every 10,000 bp. (b) The histogram shows the CNV detection threshold for the hotspot v1 chip. The data represent all the CNV calls obtained from analyzing cases with intellectual disability (ID). The number of CNVs detected in the aggregate at different size thresholds is shown on the Y-axis. We utilized a threshold of >10 probes, >1.5 z-score, and >50 kbp for CNV detection analysis. While we were easily able to detect events >50 kbp in the hotspot regions, we were only able to call variants ranging from 150 kbp (hotspot-associated CNVs) and 300 kbp onwards (mostly non-hotspot CNVs) with confidence (i.e., able to validate) for the non-hotspot regions.
(PDF)

Figure S2 Comparison of 9p24 deletions identified in our study to DECIPHER database. The figure shows genome browser snapshot of the 9p24 region with red and blue bars denoting deletions and duplications respectively in the DECIPHER database. Major clinical features observed in these patients are also shown. Please note that the black bars at the top denote CNVs identified in our study. PDD-NOS, Pervasive developmental delay-not otherwise specified; ID/DD, intellectual disability/developmental delay.
(PDF)

Figure S3 Diagnostic yield of different microarray reports from literature. Histograms show the number of rare CNVs (usually disease-associated) observed under different diagnostic centers. Data is shown for sample sizes >50. Data obtained from Table 2 of Miller et al., AJHG.
(PDF)

Table S1 Chromosomal regions and probes targeted in the hotspot chip. List of all regions targeted in the hotspot chip. The list is derived from the original curation of segmental duplication regions and genomic hotspots from Bailey et al, 2002 [14].
(PDF)

Table S2 Confirmation of CNVs arrays using custom high-density arrays. Validation of CNVs identified using NimbleGen hotspotv1 arrays (12×135 K) using a higher density 3×720K NimbleGen or 2×400K Agilent arrays.
(PDF)

Table S3 Global analysis for CNVs in neurodevelopmental disorders. Summary and characteristics of deletions and duplications identified in the four cohorts studied.
(PDF)

Table S4 (A) Characteristics of dyslexia cases from UW. Dyslexia measurements and scores of children tested at the UW. WATT- WRMT-R Woodcock Reading Mastery Test – Revised; Word Attack subtest. A measure of untimed reading of single non-words. WRAT3sp - Wide Range Achievement Tests – Third Addition; Spelling subtest. Spelling of single words from dictation in writing. WIAT(2)sp - Wechsler Individual Achievement Test (2^nd^ edition); Spelling subtest. Spelling of single words from dictation in writing. WID - WRMT-R Woodcock Reading Mastery Test – Revised; Word Identification subtest. A measure of untimed reading of single words. (B) Characteristics of dyslexia cases recruited from Atlanta. Gender, phenotype and age of children recruited from the Atlanta collection is shown.
(PDF)

Table S5 Clinical details of cases with autism (both with and without ID). Clinical features of individuals recruited through the Simons Simplex Collection (n = 350) are shown.
(PDF)

Table S6 Clinical details of cases with idiopathic ID. Clinical features of individuals with ID are shown. These individuals were recruited and evaluated at the IRCCS Associazione Oasi Maria Santissima, Troina.
(PDF)

Table S7 Clinical features of cases with ID plus MCA. Presenting clinical features, additional malformations, and preliminary cytogenetic and genetic evaluations performed for these cases are shown.
(PDF)

Table S8 (A) Comparison of rare CNV rates in the cohorts studied. (B) Rare CNVs in dyslexia, autism, and ID. (C) Inheritance of rare CNVs in the disease cohorts. (D) Individuals with two rare copy number variants (two hits).
(PDF)

Table S9 Genomic disorders identified in 1,227 cases with ID, autism, and ID/MCA. Genomic disorders are defined as copy number variants that are previously identified to be associated significantly in individuals with disease compared to controls. These CNVs can either map within genomic hotspot (HS) or non-hotspot regions (non-HS).
(PDF)

Table S10 Clinical features of a case with 6q16 deletion. Comparison of clinical features from published sources with those in the current study.
(PDF)
