# Supplementary material for: Relative Burden of Large CNVs on a Range of Neurodevelopmental Phenotypes
Source: PLoS Genet. 2011 Nov 10;7(11):e1002334. doi: 10.1371/journal.pgen.1002334 (PMC3213131; doi:10.1371/journal.pgen.1002334)

**Figure S1. Probe densities and CNV detection threshold of hotspot v1 chip.**
Although the median density of the chip was designed to be approximately 2.6 kbp in the genomic hotspots and 36 kbp in genomic backbone, the limitations of the chip design (probe assignment restricted to only up to five mismatches) precluded uniform distribution of the probes throughout the genome. Therefore, the actual probe density varied across regions of the human genome. (**a**) The plot shows the size-wise distribution of CNVs and the density of array probes targeted to the genomic hotspots and non-hotspot regions. Note that non-hotspot regions contain two different probe densities (probe spacing of 20,000 bp and about 30,000 bp labeled *a* & *b*) and the hotspots (shaded) are covered every 10,000 bp. (**b**) The histogram shows the CNV detection threshold for the hotspot v1 chip. The data represent all the CNV calls obtained from analyzing cases with intellectual disability (ID). The number of CNVs detected in the aggregate at different size thresholds is shown on the Y-axis. We utilized a threshold of >10 probes, >1.5 z-score, and >50 kbp for CNV detection analysis. While we were easily able to detect events >50 kbp in the hotspot regions, we were only able to call variants ranging from 150 kbp (hotspot-associated CNVs) and 300 kbp onwards (mostly non-hotspot CNVs) with confidence (i.e., able to validate) for the non-hotspot regions.

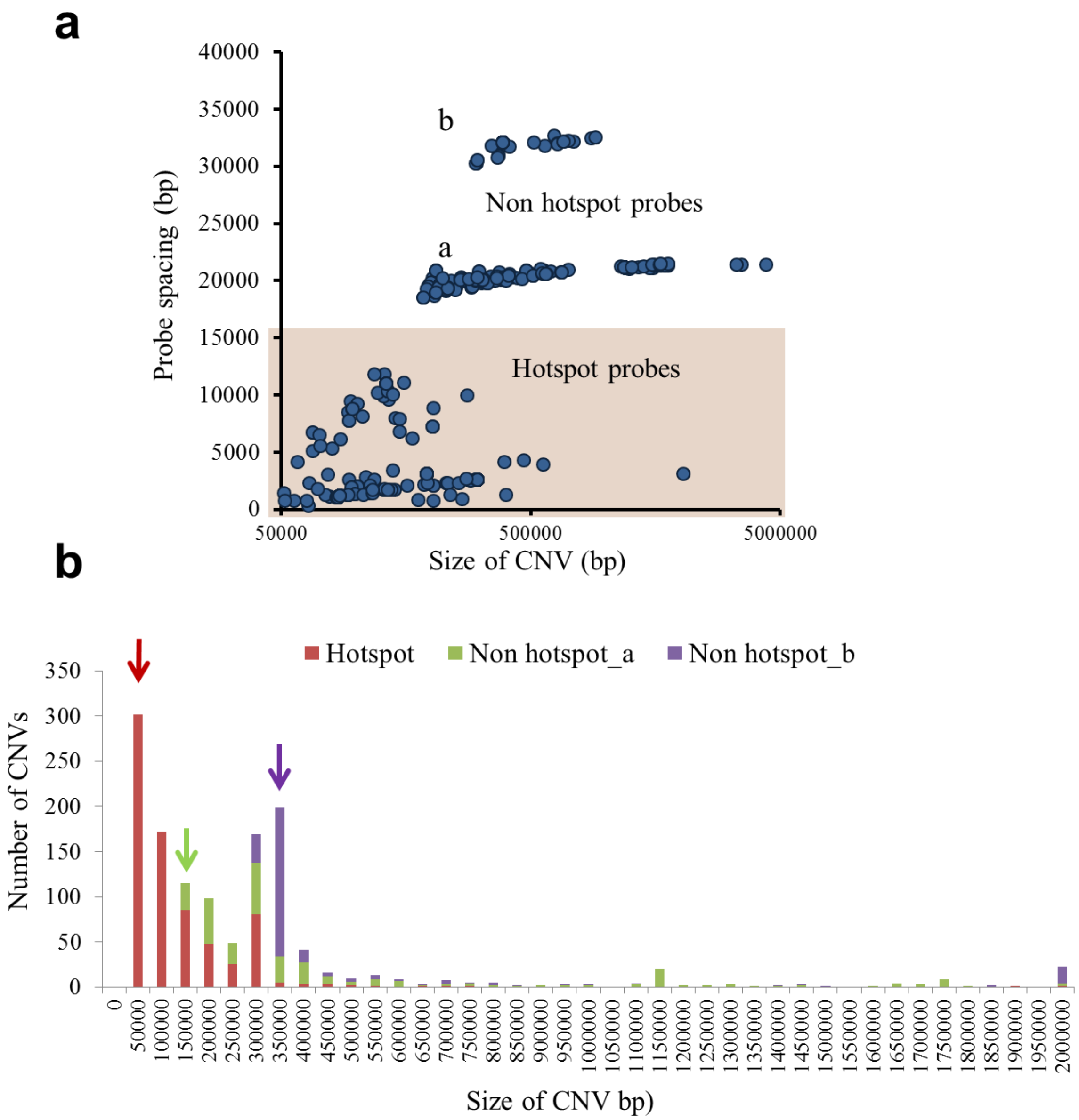

Supplement: Figure S1 — Probe densities and CNV detection threshold of hotspot v1 chip. Although the median density of the chip was designed to be approximately 2.6 kbp in the genomic hotspots and 36 kbp in genomic backbone, the limitations of the chip design (probe assignment restricted to only up to five mismatches) precluded uniform distribution of the probes throughout the genome. Therefore, the actual probe density varied across regions of the human genome. (a) The plot shows the size-wise distribution of CNVs and the density of array probes targeted to the genomic hotspots and non-hotspot regions. Note that non-hotspot regions contain two different probe densities (probe spacing of 20,000 bp and about 30,000 bp labeled a & b) and the hotspots (shaded) are covered every 10,000 bp. (b) The histogram shows the CNV detection threshold for the hotspot v1 chip. The data represent all the CNV calls obtained from analyzing cases with intellectual disability (ID). The number of CNVs detected in the aggregate at different size thresholds is shown on the Y-axis. We utilized a threshold of >10 probes, >1.5 z-score, and >50 kbp for CNV detection analysis. While we were easily able to detect events >50 kbp in the hotspot regions, we were only able to call variants ranging from 150 kbp (hotspot-associated CNVs) and 300 kbp onwards (mostly non-hotspot CNVs) with confidence (i.e., able to validate) for the non-hotspot regions. (PDF) [file pgen.1002334.s005.pdf]
