# Supplementary material for: Relative Burden of Large CNVs on a Range of Neurodevelopmental Phenotypes
Source: PLoS Genet. 2011 Nov 10;7(11):e1002334. doi: 10.1371/journal.pgen.1002334 (PMC3213131; doi:10.1371/journal.pgen.1002334)

Figure S3. Diagnostic yield of different microarray reports from literature.
Data is shown for sample sizes >50. Data obtained from Table 2 of Miller et al., AJHG.

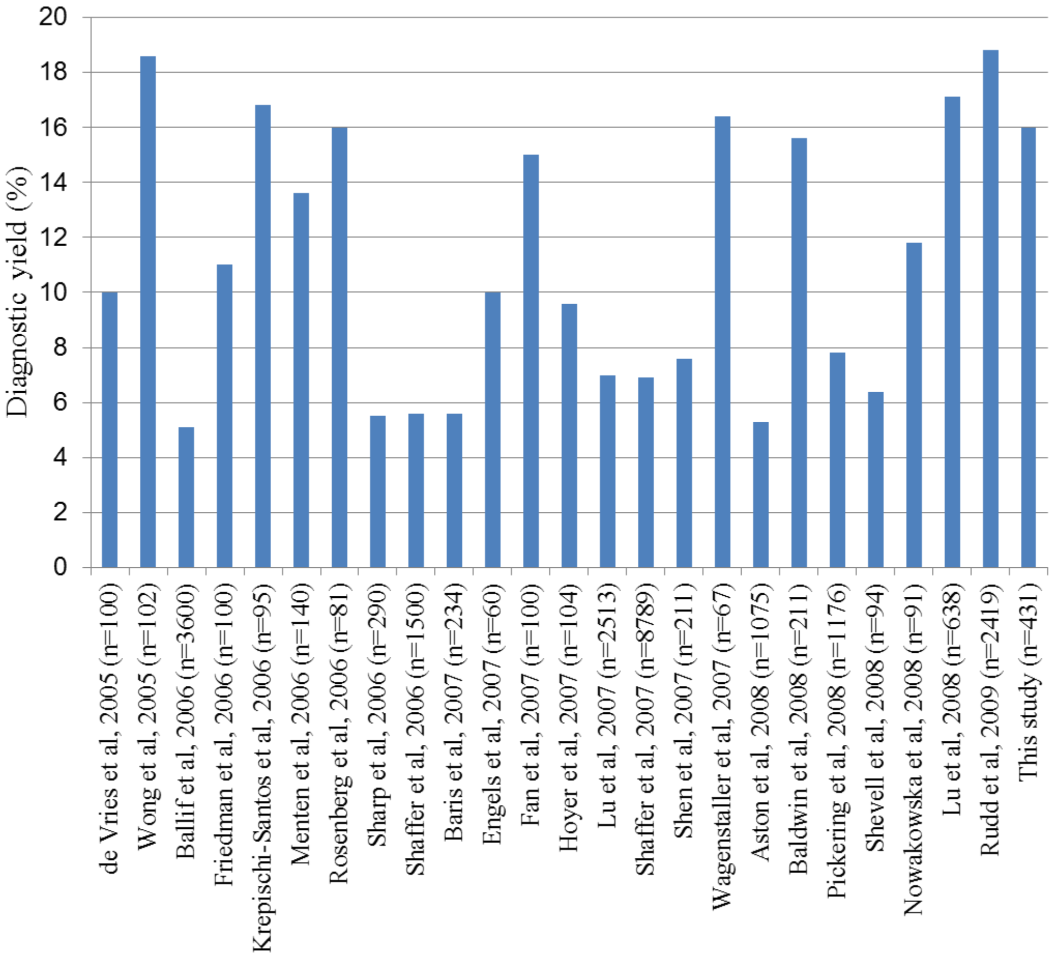

Supplement: Figure S3 — Diagnostic yield of different microarray reports from literature. Histograms show the number of rare CNVs (usually disease-associated) observed under different diagnostic centers. Data is shown for sample sizes >50. Data obtained from Table 2 of Miller et al., AJHG. (PDF) [file pgen.1002334.s007.pdf]
