# Supplementary material for: Relative Burden of Large CNVs on a Range of Neurodevelopmental Phenotypes
Source: PLoS Genet. 2011 Nov 10;7(11):e1002334. doi: 10.1371/journal.pgen.1002334 (PMC3213131; doi:10.1371/journal.pgen.1002334)
Supplement: Table S1 — Chromosomal regions and probes targeted in the hotspot chip. List of all regions targeted in the hotspot chip. The list is derived from the original curation of segmental duplication regions and genomic hotspots from Bailey et al, 2002 [14]. (PDF) [file pgen.1002334.s008.pdf]

Table S1. Chromosomal regions and probes targeted in the hotspot chip.

Chr	Start	End	Size	Number of probes	Probe spacing	Comments
chr1	39747785	40014937	267,152	101	2650	1q34.2
chr1	47159990	47395404	235,414	89	2650	1p33
chr1	108580322	108727851	147,529	56	2650	1p13.3
chr1	143500000	144800000	1,300,000	491	2650	1q21.1*
chr1	144900000	146437500	1,537,500	580	2650	1q21.1*
chr1	246650862	246900804	249,942	94	2650	1q44
chr2	86978679	89300000	2,321,321	876	2650	2p11.2
chr2	94700000	97580877	2,880,877	1087	2650	2q11.1q11.2
chr2	100063483	112904765	12,841,282	1000	12841	2q11.2q13
chr2	109800000	110900000	1,100,000	415	2650	2q11.2q13
chr2	130535282	131800847	1,265,565	478	2650	2q21.3
chr2	232901972	233023193	121,221	46	2650	2q37.1*
chr3	10065027	11908728	1,843,701	696	2650	3p25.3
chr3	125158409	131242152	6,083,743	1000	6084	3q21.1q21.3
chr3	196864663	198868245	2,003,582	756	2650	3q29*
chr4	3963456	9357304	5,393,848	1000	5394	4p16.2p16.1*
chr4	69713236	69923750	210,514	79	2650	4q13.2
chr4	70056451	70332530	276,079	104	2650	4q13.2
chr4	75501328	75712285	210,957	80	2650	4q13.3
chr4	119740794	120580940	840,146	317	2650	4q26
chr5	288806	1647139	1,358,333	513	2650	5p15.33
chr5	21540354	29492181	7,951,827	1000	7952	5p14.3p14.1
chr5	68886201	70696072	1,809,871	683	2650	5q13.2
chr5	98754229	99738292	984,063	371	2650	5q21.1
chr5	175417851	177414855	1,997,004	754	2650	5q35.2q35.3 (Sotos)*
chr6	167499260	167726129	226,869	86	2650	6q27
chr7	29659104	35197599	5,538,495	1000	5538	7p15.1p14.3
chr7	35951456	45755807	9,804,351	1000	9804	7p14.2p13
chr7	45798959	56428389	10,629,430	1000	10629	7p13p11.2
chr7	64160709	64898405	737,696	278	2650	7q11.21
chr7	66122765	71909367	5,786,602	1000	5787	7q11.22
chr7	72004121	76529725	4,525,604	1000	4526	7q11.23 (Williams)*
chr7	149217715	153493015	4,275,300	1000	4275	7q36.1q36.2
chr8	2167584	2331389	163,805	62	2650	8p23.3p23.2
chr8	6933974	12586975	5,653,001	1000	5653	8p23.1*
chr8	145259368	145429821	170,453	64	2650	8q24.3*
chr9	33372122	47107499	13,735,377	1000	13735	9p13.3p13.2
chr9	66194476	70221504	4,027,028	1000	4027	9q12q13
chr9	85632604	87600000	1,967,396	742	2650	9q21.32q21.33
chr9	89717505	89944931	227,426	86	2650	9q22.1
chr9	96108889	98751432	2,642,543	997	2650	9q22.32
chr10	27600000	28308559	708,559	267	2650	10p12.1
chr10	45491749	51585709	6,093,960	1000	6094	10q11.22
chr10	81129803	89119394	7,989,591	1000	7990	10q23.1*
chr11	48578987	51500000	2,921,013	1000	2921	11p11.2
chr11	54440000	55418071	978,071	369	2650	11q11
chr11	67312280	71193473	3,881,193	1000	3881	11q13.2q13.4

chr12	9327470	9492133	164,663	62	2650	12p13.31
chr12	34012279	37041888	3,029,609	1000	3030	12p11.1q12
chr13	18878590	24453912	5,575,322	1000	5575	13q12.11q12.12
chr15	19600000	20900000	1,300,000	491	2650	15q11.2 (BP1-BP2)*
chr15	20900000	26700000	5,800,000	1000	5800	15q11.2q13.1 (BP2-BP3)*
chr15	26700000	30687000	3,987,000	1000	3987	15q13.1q13.3 (BP3-BP5)*
chr15	70698860	73384192	2,685,332	1000	2685	15q24.1q24.2*
chr15	73760175	75985592	2,225,417	840	2650	15q24.2q24. 3*
chr15	80416103	83597521	3,181,418	1000	3181	15q25.2*
chr16	11926087	21500000	9,573,913	1000	9574	16p13.11p13.3*
chr16	21500000	28800000	7,300,000	1000	7300	16p12.1p11.2*
chr16	28800000	30254369	1,454,369	549	2650	16p11.2*
chr16	31868654	33678258	1,809,604	683	2650	16p11.2
chr16	68534999	73147662	4,612,663	1000	4613	16q22.1q22.3
chr17	2900904	3103469	202,565	76	2650	17p13.3 (Miller-Dieker)*
chr17	14014753	15750000	1,735,247	655	2650	17p12 (CMT1A/HNPP)*
chr17	15750000	18500000	2,750,000	1000	2750	17p11.2p12 (SMS)*
chr17	18500000	20750885	2,250,885	849	2650	17p11.2
chr17	21390699	21863694	472,995	178	2650	GAP
chr17	31430104	33585562	2,155,458	813	2650	17q12 (RCAD)*
chr17	33604213	43026435	9,422,222	1000	9422	17q21*
chr17	55005023	57730605	2,725,582	1000	2726	17q23.1q23.2
chr18	10594201	12221380	1,627,179	614	2650	18p11.2p11.21
chr19	22351817	22654242	302,425	114	2650	19p12
chr19	41455484	42488459	1,032,975	390	2650	19q13.12
chr19	53098555	55333144	2,234,589	843	2650	19q13.32q13.33
chr20	45887205	46571012	683,807	258	2650	20p13
chr22	15385667	23387607	8,001,940	1000	8002	22q11.2q11.23*
chr22	45200000	49500000	4,300,000	1000	4300	22q13*
chrX	36896326	37339751	443,425	167	2650	Xp12.1
chrX	48035332	52772103	4,736,771	1000	4737	Xp11.23p11.22
chrX	57617021	57972819	355,798	134	2650	Xp11.1
chrX	148421847	148866679	444,832	168	2650	Xq28
chrX	152024335	152216219	191,884	72	2650	Xq28
chrX	154219195	154387405	168,210	63	2650	Xq28
chrY	6167831	10362225	4,194,394	1000	4194	Yp11.2
chrY	22077505	23217056	1,139,551	430	2650	Yq11.23
chrY	23566258	26800000	3,233,742	1000	3234	Yq11.23q11.2

*Regions associated with known genomic disorders.
