# Supplementary material for: Relative Burden of Large CNVs on a Range of Neurodevelopmental Phenotypes
Source: PLoS Genet. 2011 Nov 10;7(11):e1002334. doi: 10.1371/journal.pgen.1002334 (PMC3213131; doi:10.1371/journal.pgen.1002334)
Supplement: Table S2 — Confirmation of CNVs arrays using custom high-density arrays. Validation of CNVs identified using NimbleGen hotspotv1 arrays (12×135 K) using a higher density 3×720K NimbleGen or 2×400K Agilent arrays. (PDF) [file pgen.1002334.s009.pdf]

Table S2. Confirmation of CNVs arrays using custom high density arrays.

Hotspot v1 calls					Validation using Agilent high density array						
chrA	startA	endA	idA	size	chrB	startB	endB	idB	size	SD (%)	Comments
chr1	76466419	77200494	T03399	734075	chr1	76471119	77195570	T03399	724451	0	non-hotspot
chr1	147781430	147965026	T02597	183596	chr1	147781430	147965026	T02597	183596	100	hotspot
chr1	246776970	246861620	T01613	84650	chr1	246776969	246877223	T01613	100254	49	hotspot
chr1	246776970	246861620	T03401	84650	chr1	246776969	246973708	T03401	196739	49	hotspot
chr1	246806247	246861620	T02457	55373	chr1	246806246	246948893	T02457	142647	22	hotspot
chr10	46200173	46840225	T03249	640052	chr10	46372252	46558714	T03249	186462	71	hotspot
chr10	46391938	46557601	T02539	165663	chr10	46382449	46557600	T02539	175151	1	hotspot
chr10	46391938	46560520	T02237	168582	chr10	46263774	46587337	T02237	323563	1	hotspot
chr10	46391938	46560520	T02237	168582	chr10	46391937	46560519	T02237	168582	1	hotspot
chr10	47006156	47209096	T01613	202940	chr10	47007445	47030359	T01613	22914	85	hotspot
chr10	47006156	47209096	T03401	202940	chr10	47007445	47207546	T03401	200101	85	hotspot
chr10	47007446	47243285	T01402	235839	chr10	47220214	47220825	T01402	611	87	hotspot
chr10	47007446	47243285	T01402	235839	chr10	47007445	47207546	T01402	200101	87	hotspot
chr10	47058972	47173905	T02511	114933	chr10	47006155	47209095	T02511	202940	74	hotspot
chr10	56612672	57015738	T02560	403066	chr10	56609630	57074555	T02560	464925	0	non-hotspot
chr10	128662416	129042087	T01402	379671	chr10	128656721	129054633	T01402	397912	0	non-hotspot
chr11	22232079	25091772	T01613	2859693	chr11	22250980	24986578	T01613	2735598	0	non-hotspot
chr11	55097313	55209463	T02457	112150	chr11	55110761	55209462	T02457	98701	0	hotspot
chr11	55097313	55209463	T03262	112150	chr11	55123517	55209462	T03262	85945	0	hotspot
chr11	55110762	55209463	T02237	98701	chr11	55110761	55209462	T02237	98701	0	hotspot
chr11	55110762	55209463	T02237	98701	chr11	55123780	55209462	T02237	85682	0	hotspot
chr11	55110762	55209463	T03187	98701	chr11	55110762	55209463	T03187	98701	0	hotspot
chr11	55123518	55209463	T01613	85945	chr11	55123780	55209462	T01613	85682	0	hotspot
chr11	55123518	55209463	T02257	85945	chr11	55123780	55209462	T02257	85682	0	hotspot
chr11	55123518	55209463	T03401	85945	chr11	55123780	55209462	T03401	85682	0	hotspot
chr11	55123518	55209463	T03413	85945	chr11	55123517	55209462	T03413	85945	0	hotspot
chr11	55123518	55209463	T03413	85945	chr11	55123780	55216262	T03413	92482	0	hotspot
chr11	55123518	55209463	T03448	85945	chr11	55123780	55209462	T03448	85682	0	hotspot
chr11	55123518	55209463	T072	85945	chr11	55123780	55209462	T072	85682	0	hotspot
chr11	55123518	55209463	T02539	85945	chr11	55123518	55209463	T02539	85945	0	hotspot
chr11	55123518	55221807	T02511	98289	chr11	55110761	55209462	T02511	98701	0	hotspot
chr11	84087102	84240239	T03099	153137	chr11	84090999	84247764	T03099	156765	0	non-hotspot
chr11	121813520	134447248	T02597	12633728	chr11	121809317	134424866	T02597	12615549	2	non-hotspot
chr13	48532965	48903297	T02522	370332	chr13	48532965	48903297	T02522	370332	0	non-hotspot
chr13	95576502	96051348	T02175	474846	chr13	95576501	96017618	T02175	441117	0	non-hotspot
chr13	112719313	113055112	T02237	335799	chr13	112766967	112767804	T02237	837	0	non-hotspot
chr15	18469959	19657464	T02597	1187505	chr15	18571399	18863527	T02597	292128	94	hotspot

chr15	18469959	19657464	T02597	1187505	chr15	19080050	19465053	T02597	385003	94	hotspot
chr15	18748445	19657464	T03249	909019	chr15	18748445	19657464	T03249	909019	92	hotspot
chr15	19080051	19465054	T02457	385003	chr15	19080050	19465053	T02457	385003	82	hotspot
chr15	19080051	19465054	T02511	385003	chr15	19080050	19465053	T02511	385003	82	hotspot
chr15	19080051	19465054	T02539	385003	chr15	19107458	19465053	T02539	357595	82	hotspot
chr15	19080051	19465054	T03262	385003	chr15	19080050	19465053	T03262	385003	82	hotspot
chr15	19080051	19465054	T03401	385003	chr15	19107458	19465053	T03401	357595	82	hotspot
chr15	19080051	19465054	T03448	385003	chr15	19107458	19465053	T03448	357595	82	hotspot
chr15	19763549	20084406	T02597	320857	chr15	19774581	20080161	T02597	305580	91	hotspot
chr15	19774582	19869177	T02511	94595	chr15	19774581	19835414	T02511	60833	100	hotspot
chr15	19774582	19922897	T02257	148315	chr15	19792418	19916386	T02257	123968	100	hotspot
chr15	19774582	20076090	T01164	301508	chr15	19774581	20076089	T01164	301508	90	hotspot
chr15	19774582	20076090	T01164	301508	chr15	19792418	19997318	T01164	204900	90	hotspot
chr15	19774582	20076090	T01164	301508	chr15	20055171	20082157	T01164	26986	90	hotspot
chr15	19774582	20080162	T02457	305580	chr15	19774581	20080161	T02457	305580	91	hotspot
chr15	19774582	20080162	T02539	305580	chr15	19792418	20082432	T02539	290014	91	hotspot
chr15	19774582	20080162	T03448	305580	chr15	19792418	20073872	T03448	281454	91	hotspot
chr15	19886399	20061404	T02511	175005	chr15	19926975	20061403	T02511	134428	84	hotspot
chr15	19886399	20080162	T03401	193763	chr15	19792418	20070064	T03401	277646	85	hotspot
chr15	19926976	19988453	T02237	61477	chr15	19792418	20094023	T02237	301605	72	hotspot
chr15	19926976	19988453	T02237	61477	chr15	19884597	20076089	T02237	191492	72	hotspot
chr15	26994610	30522495	T01879	3527885	chr15	26998905	27639098	T01879	640193	33	hotspot
chr15	26994610	30522495	T01879	3527885	chr15	26996853	28152233	T01879	1155380	33	hotspot
chr15	26994610	30522495	T01879	3527885	chr15	27942907	28031823	T01879	88916	33	hotspot
chr15	26994610	30522495	T01879	3527885	chr15	27648502	27652671	T01879	4169	33	hotspot
chr15	26994610	30522495	T01879	3527885	chr15	27668682	27890970	T01879	222288	33	hotspot
chr15	26994610	30522495	T01879	3527885	chr15	28425524	28461374	T01879	35850	33	hotspot
chr15	26994610	30522495	T01879	3527885	chr15	28064905	28295240	T01879	230335	33	hotspot
chr15	26994610	30522495	T01879	3527885	chr15	28315441	28518068	T01879	202627	33	hotspot
chr15	26994610	30522495	T01879	3527885	chr15	28703438	30225536	T01879	1522098	33	hotspot
chr15	26994610	30522495	T01879	3527885	chr15	28754787	29287087	T01879	532300	33	hotspot
chr15	26994610	30522495	T01879	3527885	chr15	29310191	29360403	T01879	50212	33	hotspot
chr15	26994610	30522495	T01879	3527885	chr15	29380916	29485266	T01879	104350	33	hotspot
chr15	26994610	30522495	T01879	3527885	chr15	29960686	30381701	T01879	421015	33	hotspot
chr15	26994610	30522495	T01879	3527885	chr15	29510391	29523381	T01879	12990	33	hotspot
chr15	26994610	30522495	T01879	3527885	chr15	29574652	29945959	T01879	371307	33	hotspot
chr15	26994610	30522495	T01879	3527885	chr15	30348746	30390543	T01879	41797	33	hotspot
chr15	26994610	30522495	T01879	3527885	chr15	30400384	30505772	T01879	105388	33	hotspot
chr15	80767738	100147041	T02522	19379303	chr15	81029877	81039845	T02522	9968	5	non-hotspot
chr15	80767738	100147041	T02522	19379303	chr15	81011780	81019879	T02522	8099	5	non-hotspot

chr15	80767738	100147041	T02522	19379303	chr15	83444383	83450742	T02522	6359	5	non-hotspot
chr15	80767738	100147041	T02522	19379303	chr15	81125842	81129261	T02522	3419	5	non-hotspot
chr15	80767738	100147041	T02522	19379303	chr15	81472226	81474343	T02522	2117	5	non-hotspot
chr15	80767738	100147041	T02522	19379303	chr15	81392480	81410164	T02522	17684	5	non-hotspot
chr15	80767738	100147041	T02522	19379303	chr15	81416166	81420168	T02522	4002	5	non-hotspot
chr15	80767738	100147041	T02522	19379303	chr15	82116141	82120064	T02522	3923	5	non-hotspot
chr15	80767738	100147041	T02522	19379303	chr15	81575659	81578551	T02522	2892	5	non-hotspot
chr15	80767738	100147041	T02522	19379303	chr15	81669530	81672359	T02522	2829	5	non-hotspot
chr15	80767738	100147041	T02522	19379303	chr15	81990994	81994086	T02522	3092	5	non-hotspot
chr15	80767738	100147041	T02522	19379303	chr15	82579682	82582077	T02522	2395	5	non-hotspot
chr15	80767738	100147041	T02522	19379303	chr15	83013827	83018237	T02522	4410	5	non-hotspot
chr15	80767738	100147041	T02522	19379303	chr15	83640941	83643184	T02522	2243	5	non-hotspot
chr15	80767738	100147041	T02522	19379303	chr15	87107799	87145348	T02522	37549	5	non-hotspot
chr15	80767738	100147041	T02522	19379303	chr15	86881518	86881875	T02522	357	5	non-hotspot
chr16	14936606	16426815	T03401	1490209	chr16	14956439	15196795	T03401	240356	45	hotspot
chr16	14936606	16426815	T03401	1490209	chr16	15213052	15224264	T03401	11212	45	hotspot
chr16	14936606	16426815	T03401	1490209	chr16	15320723	16308653	T03401	987930	45	hotspot
chr16	14936606	16426815	T03401	1490209	chr16	16368337	16432169	T03401	63832	45	hotspot
chr16	14956201	15313894	T03401	357693	chr16	14956439	15196795	T03401	240356	88	hotspot
chr16	14956201	15313894	T03401	357693	chr16	15213052	15224264	T03401	11212	88	hotspot
chr16	15386338	16177142	T03401	790804	chr16	15320723	16308653	T03401	987930	4	hotspot
chr16	22538122	22622184	T01879	84062	chr16	22465067	22618053	T01879	152986	0	hotspot
chr16	22538122	22622184	T01879	84062	chr16	22538121	22611974	T01879	73853	0	hotspot
chr16	29554938	30104150	T02257	549212	chr16	29559250	30107017	T02257	547767	0	hotspot
chr16	29560614	29619541	T02257	58927	chr16	29559250	30107017	T02257	547767	0	hotspot
chr16	29698146	30101408	T02257	403262	chr16	29559250	30107017	T02257	547767	0	hotspot
chr17	34089604	34566438	T072	476834	chr17	34107261	34553178	T072	445917	6	hotspot
chr17	34112649	34553179	T072	440530	chr17	34107261	34553178	T072	445917	6	hotspot
chr17	41781685	42145162	T01402	363477	chr17	41781685	42145162	T01402	363477	98	hotspot
chr18	50965716	52820402	T01164	1854686	chr18	50968251	51875891	T01164	907640	0	non-hotspot
chr18	50965716	52820402	T01164	1854686	chr18	50965715	52991818	T01164	2026103	0	non-hotspot
chr18	50965716	52820402	T01164	1854686	chr18	51876959	52835234	T01164	958275	0	non-hotspot
chr19	60032498	61147051	T03262	1114553	chr19	60032497	61214380	T03262	1181883	6	non-hotspot
chr2	89093011	89400666	T02560	307655	chr2	89093010	89405605	T02560	312595	93	hotspot
chr2	89571966	89921719	T02560	349753	chr2	89571965	89900508	T02560	328543	92	hotspot
chr2	188179827	188853079	T02522	673252	chr2	188179827	188853079	T02522	673252	0	non-hotspot
chr2	242392773	242736154	T01879	343381	chr2	242499630	242704103	T01879	204473	30	hotspot-associated
chr2	242392773	242736154	T01879	343381	chr2	242526867	242736153	T01879	209286	30	hotspot-associated
chr22	17011430	17292686	T02597	281256	chr22	17011430	17292686	T02597	281256	96	hotspot
chr22	18471415	18748557	T01893	277142	chr22	17274543	18748556	T01893	1474013	48	hotspot

chr22	19797358	20100802	T03249	303444	chr22	19838221	19943293	T03249	105072	100	hotspot-associated
chr3	50846910	58424157	T03448	7577247	chr3	50929636	51281103	T03448	351467	0	non-hotspot
chr3	50846910	58424157	T03448	7577247	chr3	52546856	52782978	T03448	236122	0	non-hotspot
chr3	50846910	58424157	T03448	7577247	chr3	52871729	53026639	T03448	154910	0	non-hotspot
chr3	50846910	58424157	T03448	7577247	chr3	53297489	57397354	T03448	4099865	0	non-hotspot
chr3	62489009	63295392	T03445	806383	chr3	62486882	63317886	T03445	831004	0	non-hotspot
chr3	62489781	63320060	T03445	832020	chr3	62486882	63317886	T03445	831004	1	hotspot-associated
chr3	127000260	131353408	T02237	4353148	chr3	128948483	128994675	T02237	46192	10	hotspot-associated
chr3	127000260	131353408	T02237	4353148	chr3	130143643	130167164	T02237	23521	10	hotspot-associated
chr3	127000260	131353408	T02237	4353148	chr3	130697089	130705883	T02237	8794	10	hotspot-associated
chr3	127000260	131353408	T02237	4353148	chr3	131273933	131289308	T02237	15375	10	hotspot-associated
chr3	163645814	164487738	T02539	841924	chr3	163666597	163990861	T02539	324264	0	non-hotspot
chr3	163645814	164487738	T02539	841924	chr3	164100819	164636767	T02539	535948	0	non-hotspot
chr3	163645815	164649782	T02539	1003967	chr3	163666597	163990861	T02539	324264	0	non-hotspot
chr3	163645815	164649782	T02539	1003967	chr3	164100819	164636767	T02539	535948	0	non-hotspot
chr3	163679745	164557737	T02457	877992	chr3	163679744	164487737	T02457	807993	0	non-hotspot
chr3	180461081	180763350	T03401	302269	chr3	180468733	180628891	T03401	160158	2	hotspot-associated
chr3	196881289	196950899	T03401	69610	chr3	196910403	196961676	T03401	51273	45	hotspot
chr3	196906336	196958628	T03445	52292	chr3	196882266	197001549	T03445	119283	13	hotspot
chr3	197188186	198861094	T03249	1672908	chr3	197231467	197352530	T03249	121063	5	hotspot-associated
chr3	197188186	198861094	T03249	1672908	chr3	197442329	197843020	T03249	400691	5	hotspot-associated
chr3	197188186	198861094	T03249	1672908	chr3	198246793	198534763	T03249	287970	5	hotspot-associated
chr3	197188186	198861094	T03249	1672908	chr3	197862429	197869846	T03249	7417	5	hotspot-associated
chr3	197188186	198861094	T03249	1672908	chr3	197877081	198111628	T03249	234547	5	hotspot-associated
chr3	197188186	198861094	T03249	1672908	chr3	198132998	198158201	T03249	25203	5	hotspot-associated
chr3	197188186	198861094	T03249	1672908	chr3	198167857	198177065	T03249	9208	5	hotspot-associated
chr3	197188186	198861094	T03249	1672908	chr3	198639865	198650589	T03249	10724	5	hotspot-associated
chr3	197188186	198861094	T03249	1672908	chr3	198690562	198694242	T03249	3680	5	hotspot-associated
chr3	197188186	198861094	T03249	1672908	chr3	198789801	198799183	T03249	9382	5	hotspot-associated
chr4	70072160	70285818	T01402	213658	chr4	70062193	70088108	T01402	25915	98	hotspot-associated
chr4	70072160	70285818	T01402	213658	chr4	70160392	70264770	T01402	104378	98	hotspot-associated
chr4	70142222	70261760	T02539	119538	chr4	70190808	70264770	T02539	73962	97	hotspot-associated
chr4	70152740	70268157	T02511	115417	chr4	70153463	70261759	T02511	108296	97	hotspot-associated
chr4	70162889	70261760	T03401	98871	chr4	70177280	70264770	T03401	87490	96	hotspot-associated

chr4	70162889	70261760	T03445	98871	chr4	70160392	70264770	T03445	104378	96	hotspot-associated
chr4	70165722	70261760	T02237	96038	chr4	70160392	70264770	T02237	104378	96	hotspot-associated
chr4	70165722	70261760	T02237	96038	chr4	70184221	70260690	T02237	76469	96	hotspot-associated
chr5	714831	849094	T02237	134263	chr5	734090	866073	T02237	131983	86	hotspot-associated
chr5	714831	849094	T02237	134263	chr5	719383	849093	T02237	129710	86	hotspot-associated
chr5	717373	849094	T02539	131721	chr5	734090	901677	T02539	167587	88	hotspot-associated
chr5	717373	874583	T02257	157210	chr5	734090	901677	T02257	167587	88	hotspot-associated
chr5	719384	814246	T03448	94862	chr5	714830	748527	T03448	33697	87	hotspot
chr5	730867	849094	T02457	118227	chr5	745887	849093	T02457	103206	98	hotspot-associated
chr5	1051432	1391721	T02237							NA	not validated
chr5	68876515	70650796	T02597	1774281	chr5	70342727	70345397	T02597	2670	100	hotspot
chr5	69087097	70315414	T03249	1228317	chr5	69087097	70315414	T03249	1228317	100	hotspot
chr5	69087097	70344444	T01402	1257347	chr5	69108248	69151466	T01402	43218	100	hotspot
chr5	69087097	70344444	T01402	1257347	chr5	69274221	69506747	T01402	232526	100	hotspot
chr5	69087097	70344444	T01402	1257347	chr5	69688060	69700814	T01402	12754	100	hotspot
chr5	69087097	70344444	T01402	1257347	chr5	69979510	69987295	T01402	7785	100	hotspot
chr5	69087097	70344444	T01402	1257347	chr5	70161181	70330293	T01402	169112	100	hotspot
chr5	70441720	70650796	T01402	209076	chr5	70426301	70507131	T01402	80830	100	hotspot
chr5	156526018	164133824	T03448	7607806	chr5	156573853	159472988	T03448	2899135	0	hotspot-associated
chr5	156526018	164133824	T03448	7607806	chr5	159671194	164135198	T03448	4464004	0	hotspot-associated
chr7	75974196	76384959	T02560	410763	chr7	75891314	76477399	T02560	586085	100	hotspot
chr7	149105718	149484098	T02597	378380	chr7	149060871	149548751	T02597	487880	70	hotspot-associated
chr8	7242905	7451662	T01402	208757	chr8	7242905	7451662	T01402	208757	100	hotspot
chr8	7562522	7971704	T01402	409182	chr8	7562522	7971704	T01402	409182	100	hotspot
chr8	39173157	39477281	T03401	304124	chr8	39353969	39504514	T03401	150545	1	hotspot-associated
chr8	39352427	39506424	T03187	153997	chr8	39352427	39506424	T03187	153997	2	hotspot-associated
chr9	11765283	12138404	T02560	373121	chr9	11775234	11868161	T02560	92927	1	hotspot-associated
chr9	11765283	12138404	T02560	373121	chr9	12013043	12149108	T02560	136065	1	hotspot-associated
chr9	73827781	79830447	T03413	6002666	chr9	73822864	79779763	T03413	5956899	0	non-hotspot
chr9	73827781	79830447	T03413	6002666	chr9	73830596	79814193	T03413	5983597	0	non-hotspot
