# Supplementary material for: Relative Burden of Large CNVs on a Range of Neurodevelopmental Phenotypes
Source: PLoS Genet. 2011 Nov 10;7(11):e1002334. doi: 10.1371/journal.pgen.1002334 (PMC3213131; doi:10.1371/journal.pgen.1002334)
Supplement: Table S3 — Global analysis for CNVs in neurodevelopmental disorders. Summary and characteristics of deletions and duplications identified in the four cohorts studied. (PDF) [file pgen.1002334.s010.pdf]

Table S3. Global analysis for CNVs in neurodevelopmental disorders

					Deletions		Duplications	
	Total CNVs	Del/Dup ratio	Percent deletions	Median size	Total deletions	Perc gene disrupting	Total duplications	Perc gene disrupting
Controls	1,074	0.605	0.377	167,749	405	0.26	669	0.37
Dyslexia	1,041	0.587	0.370	161,522	385	0.22	656	0.42
Autism No ID	923	0.482	0.325	157,556	300	0.20	623	0.43
Autism with ID	362	0.692	0.409	168,582	148	0.22	214	0.41
Autism	1,285	0.535	0.349	167,749	448	0.21	837	0.43
ID	1,306	0.499	0.333	225,272	435	0.27	871	0.43
ID/MCA	380	0.551	0.355	305,580	135	0.36	245	0.41
Combined ID	1,686	0.511	0.338	247,603	570	0.29	1,116	0.43
