# Supplementary material for: Relative Burden of Large CNVs on a Range of Neurodevelopmental Phenotypes
Source: PLoS Genet. 2011 Nov 10;7(11):e1002334. doi: 10.1371/journal.pgen.1002334 (PMC3213131; doi:10.1371/journal.pgen.1002334)
Supplement: Table S4 — (A) Characteristics of dyslexia cases from UW. Dyslexia measurements and scores of children tested at the UW. WATT- WRMT-R Woodcock Reading Mastery Test – Revised; Word Attack subtest. A measure of untimed reading of single non-words. WRAT3sp - Wide Range Achievement Tests – Third Addition; Spelling subtest. Spelling of single words from dictation in writing. WIAT(2)sp - Wechsler Individual Achievement Test (2nd edition); Spelling subtest. Spelling of single words from dictation in writing. WID - WRMT-R Woodcock Reading Mastery Test – Revised; Word Identification subtest. A measure of untimed reading of single words. (B) Characteristics of dyslexia cases recruited from Atlanta. Gender, phenotype and age of children recruited from the Atlanta collection is shown. (PDF) [file pgen.1002334.s011.pdf]

Table S4A. Characteristics of dyslexia cases from UW

Sample code	proband	birthdate	famid	sex	ethnicity	comment	viq	wid	watt	wrat3sp	wiat2sp	wiatsp
852	1	12/25/1981	010	M	Caucasian	In writing tutorial and imaging studies.	112	95	92	88	.	80
782	1	9/10/1982	012	F	Native American	math is very strong. In writing tutorial.	94	82	80	78	.	91
959	1	1/8/1983	025	M	Caucasian	in writing tutorial. Consultation with mom re: IEP	100	68	72	75	.	72
836	1	4/21/1985	042	M	Caucasian	In reading tutorial.	111	92	96	102	.	91
1102	1	6/14/1982	047	F	Caucasian	.	122	86	91	90	.	88
1114	1	12/13/1980	053	M	Caucasian	.	100	71	76	78	.	74
1633	1	2/14/1982	073	F	Caucasian	.	105	76	82	83	.	79
957	1	1/18/1984	075	F	Caucasian	Consulted with school for IEP - sent phonological training program.	105	74	79	85	.	80
1163	1	2/15/1985	076	M	Caucasian	language delay - speech therapy for articulation problems. consultation with school - tutoring 1998	95	59	71	63	.	73
1149	1	11/26/1983	087	M	Caucasian	Very high IQ (147)	147	96	80	96	.	82
1182	1	3/2/1985	094	M	Caucasian	.	110	78	83	80	.	84
1201	1	11/2/1985	096	M	Caucasian	.	129	69	65	65	.	79
1311	1	4/15/1985	105	M	Caucasian	.	110	68	77	72	.	80
1287	1	5/13/1986	106	M	Caucasian	.	110	81	88	83	.	91
1260	1	7/3/1986	111	M	Caucasian	"Some pregnancy complications, toxemia."	106	70	86	83	.	91
1328	1	2/10/1981	114	M	Caucasian	.	98	83	89	75	.	77
1349	1	5/25/1984	119	M	Caucasian	"Premature birth, many complications, identified early"	95	67	86	81	.	82
1358	1	10/7/1983	122	F	Caucasian	.	110	69	84	81	.	81
1369	1	10/8/1982	126	F	Caucasian	.	93	78	75	80	.	74

1554	1	1/26/1982	132	M	Caucasian	.	118	70	79	68	.		65
1840	1	3/29/1987	139	M	Caucasian	.	101	63	82	70	.		76
2250	1	1/0/1900	202	M	Caucasian	.	140	65	73	69	68	.	
2236	1	1/0/1900	204	F	Caucasian	.	94	52	45	62	59	.	
2284	1	1/0/1900	205	M	Caucasian	.	117	83	96	80	93	.	
2244	1	1/0/1900	207	M	Caucasian	.	99	64	81	69	67	.	
2264	1	1/0/1900	209	M	Caucasian	.	106	53	65	65	60	.	
2242	1	1/0/1900	210	M	Caucasian	.	98	83	92	90	85	.	
2235	1	1/0/1900	212	M	Caucasian	.	125	93	83	87	77	.	
2266	1	1/0/1900	214	F	Caucasian	.	98	75	74	82	77	.	
2280	1	1/0/1900	215	F	Caucasian	.	92	76	84	81	76	.	
2293	1	1/0/1900	216	M	Caucasian	.	100	93	83	85	84	.	
2330	1	1/0/1900	220	M	Asian	.	117	105	93	78	76	.	
2288	1	1/0/1900	221	F	Caucasian	.	123	89	103	87	81	.	
2341	1	1/0/1900	223	M	African American	.	118	92	96	83	84	.	
2335	1	1/0/1900	224	M	Caucasian	.	104	94	95	93	82	.	
2407	1	1/0/1900	225	M	Caucasian	.	117	95	91	83	76	.	
2328	1	1/0/1900	227	M	Caucasian	.	117	66	79	73	65	.	
2411	1	1/0/1900	228	F	Caucasian	.	104	95	89	84	82	.	
2394	1	1/0/1900	229	M	Caucasian	.	115	89	83	81	78	.	
2364	1	1/0/1900	230	F	Caucasian	.	104	84	81	88	86	.	
2315	1	1/0/1900	231	M	Caucasian	.	119	93	94	87	96	.	
2349	1	1/0/1900	232	M	Caucasian	.	125	99	86	93	86	.	
2342	1	1/0/1900	233	M	Caucasian	.	111	76	67	82	76	.	
2333	1	1/0/1900	237	F	Asian	.	101	88	88	81	80	.	
2370	1	1/0/1900	239	M	Caucasian	.	90	59	67	76	84	.	
2397	1	1/0/1900	240	M	Caucasian	.	128	82	87	88	78	.	
2400	1	1/0/1900	241	M	Caucasian	.	119	67	82	75	66	.	
2382	1	1/0/1900	242	F	Caucasian	.	105	91	83	87	86	.	
2383	1	1/0/1900	243	F	Caucasian	.	106	89	87	80	83	.	
2391	1	1/0/1900	244	M	Caucasian	.	104	69	67	75	75	.	
2450	1	1/0/1900	246	F	Caucasian	.	118	86	88	87	90	.	
2446	1	1/0/1900	249	F	Caucasian	.	142	132	127	104	105	.	
2449	1	1/0/1900	250	F	Caucasian	.	98	63	62	75	65	.	
2556	1	1/0/1900	258	M	Caucasian	.	99	96	100	100	92	.	
2508	1	1/0/1900	259	F	Other	.	105	92	78	87	84	.	
2478	1	1/0/1900	260	M	Caucasian	.	125	88	77	69	57	.	
2477	1	1/0/1900	261	F	African American	.	137	68	84	79	73	.	
2522	1	1/0/1900	265	M	Caucasian	.	105	85	84	90	83	.	
2818	1	1/0/1900	266	F	Caucasian	.	124	116	113	116	110	.	

2618	1	1/0/1900	267	M	Caucasian	.	92	74	66	69	70	.
2545	1	1/0/1900	268	M	Caucasian	.	112	75	79	85	87	.
2585	1	1/0/1900	270	M	Caucasian	.	90	63	49	69	69	.
2626	1	1/0/1900	271	F	Asian	.	100	56	74	81	76	.
2620	1	1/0/1900	272	F	Caucasian	.	111	96	96	90	90	.
2551	1	1/0/1900	277	M	Caucasian	.	107	62	66	73	67	.
2564	1	1/0/1900	278	F	Caucasian	.	110	70	84	82	80	.
2568	1	1/0/1900	279	M	Caucasian	.	115	96	91	95	94	.
2558	1	1/0/1900	280	F	Caucasian	.	105	88	98	93	94	.
2774	1	1/0/1900	282	M	Caucasian	.	113	99	104	98	111	.
2602	1	1/0/1900	284	M	Caucasian	.	119	77	68	78	70	.
2609	1	1/0/1900	285	M	Caucasian	.	101	82	68	73	76	.
2619	1	1/0/1900	286	M	Caucasian	.	100	88	73	77	73	.
2642	1	1/0/1900	288	F	Caucasian	.	90	73	64	78	76	.
2804	1	1/0/1900	290	M	Caucasian	.	107	56	81	87	87	.
2760	1	1/0/1900	294	M	Other	.	110	90	91	78	71	.
2830	1	1/0/1900	304	M	Caucasian	.	110	98	100	88	79	.
2835	1	8/20/1984	307	M	Caucasian	.	104	96	94	88	91	.
2824	1	1/0/1900	310	F	Caucasian	.	112	92	96	85	88	.
2858	1	3/18/1985	313	M	Caucasian	.	100	83	78	78	76	.
2893	1	4/18/1988	317	M	Caucasian	.	135	111	106	96	96	.
2877	1	1/0/1900	318	F	Unknown	.	127	101	93	85	79	.
2867	1	2/7/1988	319	M	Caucasian	.	122	102	97	96	94	.
2888	1	11/6/1987	320	F	Caucasian	.	107	98	94	85	89	.
2881	1	6/30/1988	321	F	Caucasian	.	119	96	93	96	92	.
2864	1	3/21/1983	324	M	Native American	.	115	92	84	75	73	.
2937	1	9/27/1987	330	F	Caucasian	.	125	98	96	90	87	.
2944	1	4/6/1986	331	M	Caucasian	.	111	86	79	87	86	.
2954	1	12/20/1986	334	M	Caucasian	.	95	94	78	85	80	.
2967	1	12/28/1986	337	F	Caucasian	.	123	97	95	101	98	.
3017	1	7/6/1987	344	M	Caucasian	.	99	87	77	90	84	.
3015	1	6/15/1989	345	F	Caucasian	.	111	79	77	75	78	.
3395	1	3/15/1989	353	M	Unknown	.	99	92	83	88	94	.
3389	1	3/23/1988	355	M	Unknown	.	105	39	57	65	49	.
3397	1	11/11/1992	357	M	Unknown	.	122	105	105	90	101	.
874	1	5/7/1981	001	M	Caucasian	Is in reading tutoring with Sylvia (older group). ADHD Inattentive.	115	91	96	93	.	91
751	1	1/29/1983	002	F	Caucasian	Was in pilot tutorial for	106	83	91	84	.	93

						reading/writing.						
731	1	4/27/1983	003	M	Caucasian	math is strong. Was in pilot reading/writing tutorial.	123	109	96	105	.	105
875	1	2/19/1983	004	F	Caucasian	was in pilot reading/writing tutorial.	119	107	105	113	.	110
641	1	5/31/1984	005	F	Caucasian	"Very poor on orthographic choice; couldn't complete. Late sentences, offered tutoring but she dropped out."	90	81	85	90	.	82
924	1	8/4/1983	006	M	Native American	offered tutoring - dropped out because of transportation problems.	90	75	80	82	.	86
938	1	5/16/1981	009	F	Caucasian	In reading tutorial for older students.	117	96	89	85	.	87
865	1	4/17/1982	011	M	Caucasian	In reading and writing tutorials and brain imaging. ADHD Inattentive but no dr. diagnosis.	111	74	80	81	.	89
854	1	12/29/1981	013	M	Caucasian	delayed in single words and sentences.	129	74	63	70	.	68
1026	1	1/0/1900	014	M	Caucasian	family dropped from study						
898	1	12/4/1980	015	M	Caucasian	"mild bilateral hearing loss. 3 1/2 uneven language development. Services in physical therapy, speech services, and special education. ADHD INattentive"	110	102	96	85	.	83
1456	1	6/19/1984	016	M	Caucasian	.	125	88	90	90	.	94
762	1	2/8/1984	018	M	Caucasian	hypertonia as an infant. has had a lot of special	90	92	92	76	.	86

						education services.						
838	1	4/19/1981	019	F	Caucasian	.	119	95	89	82	.	81
844	1	10/2/1982	021	M	Caucasian	was in pilot for reading/writing tutorial	110	78	81	87	.	85
1045	1	7/5/1981	022	M	Caucasian	.	107	92	78	88	.	79
948	1	9/1/1982	023	F	Caucasian	in writing tutorial	128	82	89	81	.	85
886	1	9/23/1982	024	F	Caucasian	Is in the writing tutorial.	111	85	89	81	.	87
908	1	10/24/1982	027	M	Caucasian	Was in reading tutorial; dropped out.	112	74	77	78	.	81
879	1	9/13/1983	029	M	Caucasian	In reading tutorial.						
794	1	5/3/1983	030	M	Caucasian	"math is strong, writing tutorial"	98	74	78	81	.	82
769	1	2/28/1985	032	M	Caucasian	In reading tutoring.	112	93	94	90	.	91
799	1	7/1/1983	034	M	Caucasian	Was in writing tutorial but dropped out because of transportation problems.	125	107	96	97	.	104
824	1	10/24/1982	035	M	Asian	"In writing, tutoring and imaging studies."	105	63	71	72	.	72
876	1	10/23/1984	036	M	Caucasian	Is in reading tutorial. Genotyped at Marshfield.	141	69	89	82	.	85
781	1	12/7/1982	037	M	Native American	Based on tests at the UW in 8/96 and by private testing in 5/96. Private tutoring in Oregon.	115	79	82	78	.	87
793	1	4/20/1983	038	F	Caucasian	very strong in math. consulted with school.	115	85	75	84	.	90
776	1	3/9/1982	039	M	Caucasian	"ritalin no writing sample score. Offered writing tutorial - declined. Diagnosed with ADHD but on Ritalin, so questionnaire doesn't reflect	106	89	90	96	.	92

						symptoms."						
909	1	2/2/1983	040	M	Caucasian	"Anxious by history. Receives counseling. In writing tutorial, dropped out."	128	82	95	81	.	75
816	1	3/6/1982	041	F	Caucasian	Consulted with school via written report and conversation with mother. In reading tutorial (older group)	107	90	98	96	.	100
857	1	10/14/1982	043	F	Caucasian	received reading tutoring (older group)	100	82	88	87	.	81
889	1	4/16/1983	045	M	Hispanic	not on ritalin - change handedness to 100% right. IN reading tutoring and imaging studies. ADHD Combined	119	65	80	78	.	80
803	1	3/31/1985	048	F	Caucasian	no GORT oral reading quotient Consulted with father -- offered tutoring -- didn't participate.	106	91	85	94	.	96
801	1	7/14/1981	049	M	Caucasian	lives too far away for tutoring.	98	82	82	85	.	79
0	1	6/18/1983	050	M	Caucasian	Severely learning disabled in all domains. Unclear what role ADD plays in his learning problems. Has been very resistant in writing tutorial.	127	44	54	63	.	69
797	1	12/30/1980	054	F	East Indian	Only affected in spelling at this time. Mild phonological problems. May have compensated	107	97	95	90	.	83

						by this age (6th grade). Math is strong						
872	1	6/24/1982	056	F	Caucasian	very high IQ - was in tutoring - dropped out because of scheduling problems.	131	108	101	88	.	92
0	1	7/19/1983	059	F	Caucasian	.	101	55	72	77	.	75
1096	1	2/8/1982	060	M	Caucasian	.	112	74	76	76	.	72
887	1	1/22/1980	061	F	Caucasian	In reading tutorial for older students.ADHD Inattentive	98	71	82	75	.	73
923	1	9/4/1980	062	F	Caucasian	language delay. Tutoring summer 1988. Taking Ritalin so questionnaire reflects behavior on medication.	99	67	65	78	.	73
893	1	4/5/1981	063	M	Caucasian	Consulted with tutor and school.	98	67	66	83	.	73
894	1	6/23/1984	064	M	Caucasian	language delays - age 3	112	82	88	91	.	88
899	1	4/3/1983	066	M	Native American	math strong - delayed in talking in sentences. In reading and writing tutorial.	104	80	74	90	.	87
915	1	7/11/1983	067	M	Asian	In reading tutorial for older students.	135	107	98	99	.	105
939	1	6/9/1985	070	F	Caucasian	2 months premature but no reported language delays.	98	61	70	82	.	87
922	1	6/26/1981	071	M	Caucasian	.	92	93	92	83	.	83
1212	1	2/13/1985	072	M	Caucasian	.	144	103	92	97	.	95
956	1	10/23/1982	074	M	Caucasian	Tutoring summer 1998.	108	85	67	76	.	86
1004	1	4/30/1981	077	M	Caucasian	Eligible for tutoring in summer 1998	107	89	65	75	.	76
1012	1	1/14/1983	078	F	Caucasian	Consultation with mother -- summer	111	99	88	85	.	78

						tutoring 1998.						
1022	1	7/12/1982	079	M	Caucasian	language milestones were mildly delayed. Tutoring summer 1988 -- will drive from Bellingham. ADHD Inattentive.	92	49	64	65	.	68
1037	1	10/8/1982	081	F	Caucasian	.	98	64	79	73	.	74
1047	1	1/0/1900	083	F	Unknown	.						
1060	1	5/6/1986	084	M	Caucasian	Consultation with mother who gave her instructional materials. Tutoring Summer 1988.	124	92	97	95	.	98
1081	1	8/16/1985	085	M	Caucasian	.	110	100	92	89	.	96
1121	1	5/22/1985	086	M	Caucasian	.	90	67	76	76	.	73
1133	1	7/4/1983	088	F	Hispanic	High IQ	126	107	99	92	.	103
1136	1	6/17/1982	089	M	Caucasian	High IQ. Diagnosed as ADHD.	125	85	90	85	.	81
1161	1	12/26/1984	090	M	Caucasian	.	123	99	94	91	.	87
1173	1	1/8/1982	091	M	Caucasian	.	128	67	81	67	.	73
1169	1	1/0/1900	092	M	Caucasian	.						
1221	1	5/9/1983	093	M	Caucasian	ADHD Inattentive	112	103	95	92	.	96
1209	1	11/9/1982	095	M	Caucasian	.	106	95	108	104	.	98
1210	1	10/19/1984	097	M	Caucasian	.	133	106	87	82	.	80
1252	1	4/2/1982	098	M	Caucasian	.	93	63	70	72	.	70
1280	1	3/31/1985	099	M	Caucasian	.	118	69	74	61	.	76
1272	1	10/8/1980	100	M	African American	.	90	68	80	69	.	75
1240	1	5/10/1983	101	M	Caucasian	.	117	88	88	90	.	80
1379	1	7/2/1982	102	M	Caucasian	very affected with ADHD. ADHD Combined	118	99	94	86	.	83
1340	1	5/24/1984	103	F	Caucasian	.	102	91	80	78	.	86
1242	1	5/22/1983	104	F	Caucasian	.	112	83	89	88	.	90
1305	1	5/19/1985	107	M	Caucasian	.	123	94	98	84	.	88
1355	1	5/18/1983	108	M	Caucasian	.	101	59	67	62	.	70
1263	1	3/20/1984	109	M	Caucasian	.	90	41	65	66	.	68
1275	1	5/18/1980	110	M	Caucasian	.	100	80	84	71	.	67

1257	1	11/21/1982	112	F	Caucasian	.	94	81	78	88	.			82
1284	1	4/26/1982	113	F	Caucasian	"Fine and gross motor delays, diagnosed with 'sensory integration disorder', problems since infancy."	105	83	89	80	.			0
1278	1	10/5/1981	115	M	Caucasian	.	101	58	95	75	.			71
1342	1	6/5/1982	116	F	African American	.	104	84	78	80	.			75
1367	1	7/5/1980	117	M	Caucasian	.	105	88	100	74	.			79
1335	1	3/22/1982	120	F	Caucasian	.	99	72	86	80	.			73
1319	1	2/24/1982	121	F	Native American	ADHD Combined	94	71	82	78	.			83
1395	1	4/21/1980	127	M	Hispanic	.	100	81	78	67	.			70
1403	1	1/31/1984	128	M	Caucasian	.	112	80	75	87	.			85
1409	1	10/30/1982	129	M	Caucasian	.	128	96	85	93	.			93
1411	1	7/18/1984	130	M	Caucasian	.	112	83	79	77	.			82
1417	1	9/30/1984	131	M	Caucasian	.	98	48	68	79	.			70
1583	1	8/9/1984	133	M	Caucasian	.	112	106	110	110	.			118
1726	1	4/20/1982	134	M	Caucasian	.	90	76	85	80	.			73
1759	1	6/19/1984	135	M	Caucasian	.	90	55	74	68	.			69
1714	1	6/11/1987	136	M	Caucasian	.	135	96	100	106	.			103
1806	1	4/4/1983	137	F	Caucasian	.	122	89	77	88	.			86
2231	1	1/0/1900	201	M	Caucasian	.	119	99	93	86		75	.	
2232	1	1/0/1900	203	M	Caucasian	.	118	84	80	83		81	.	
2229	1	1/0/1900	206	M	Caucasian	.	94	73	82	75		74	.	
2290	1	1/0/1900	208	M	Caucasian	Only 1.7 micrograms. Need a blood sample.<br><br>Second sample taken with Ora Gene sputum kit 2004.	93	93	91	85		84	.	
2270	1	1/0/1900	211	M	Caucasian	.	110	84	87	75		71	.	
2274	1	1/0/1900	217	M	Caucasian	.	118	93	88	74		76	.	
2286	1	1/0/1900	218	M	Caucasian	.	119	80	89	85		79	.	
2300	1	1/0/1900	222	M	Caucasian	.	104	113	109	94		96	.	
2314	1	1/0/1900	226	M	Caucasian	.	98	57	72	75		71	.	
2346	1	1/0/1900	235	M	Caucasian	.	119	109	101	92		93	.	
2369	1	1/0/1900	238	M	Caucasian	.	92	74	80	74		70	.	
2398	1	1/0/1900	245	M	Caucasian	.	107	98	90	90		80	.	

2479	1	1/0/1900	247	M	Caucasian	.	101	65	83	69	60	.
2418	1	1/0/1900	248	M	Caucasian	.	98	84	99	80	76	.
2435	1	1/0/1900	251	F	Unknown	.						
2448	1	1/0/1900	252	M	Caucasian	.	117	71	69	81	78	.
2510	1	1/0/1900	253	M	Caucasian	.	129	97	90	94	96	.
2525	1	1/0/1900	255	M	Caucasian	.	118	105	101	111	107	.
2484	1	1/0/1900	256	M	Caucasian	.	113	90	94	85	86	.
2517	1	1/0/1900	257	M	Caucasian	.	93	90	82	82	85	.
2513	1	1/0/1900	262	M	Caucasian	.	110	89	88	95	88	.
2493	1	1/0/1900	263	F	Caucasian	.	113	81	79	88	87	.
2532	1	1/0/1900	264	F	Caucasian	.	139	99	90	90	87	.
2499	1	1/0/1900	269	M	Unknown	.						
2624	1	1/0/1900	274	F	Asian	.	99	64	77	78	80	.
2552	1	1/0/1900	275	F	Unknown	.						
2614	1	1/0/1900	276	F	Caucasian	.	99	89	97	94	87	.
2569	1	1/0/1900	281	F	Caucasian	.	90	83	85	90	77	.
2658	1	1/0/1900	283	M	Caucasian	.	131	115	100	92	102	.
2715	1	1/0/1900	289	M	Caucasian	.	100	79	72	87	78	.
2738	1	1/0/1900	291	M	Caucasian	.	119	83	100	77	89	.
2673	1	1/0/1900	292	M	Unknown	.	101	64	74	64	79	.
2766	1	1/0/1900	293	F	Caucasian	.	105	94	89	88	93	.
2749	1	1/0/1900	295	F	Other	.	100	109	103	83	77	.
2756	1	1/0/1900	296	F	Caucasian	.	112	90	81	93	85	.
2832	1	9/12/1985	297	M	Unknown	.						
2972	1	1/0/1900	298	F	Caucasian	.	111	96	95	88	88	.
2852	1	12/18/1986	299	M	Caucasian	.	110	98	103	90	84	.
2753	1	1/0/1900	300	M	Other	.	119	86	86	90	83	.
2811	1	1/0/1900	301	M	Caucasian	.	112	91	90	90	85	.
2837	1	9/27/1985	303	M	Caucasian	.	129	99	89	90	85	.
2963	1	4/27/1987	305	M	Caucasian	.	142	114	108	109	92	.
2826	1	10/28/1989	306	M	Caucasian	.	107	88	94	91	89	.
2810	1	1/0/1900	308	M	Caucasian	.	105	78	79	77	68	.
2834	1	6/14/1985	309	F	Caucasian	.	135	113	96	97	86	.
2833	1	6/13/1987	314	F	Caucasian	.	125	91	90	88	85	.
2863	1	6/15/1983	315	M	Caucasian	.	110	90	82	77	73	.
2868	1	1/25/1988	316	F	Caucasian	.	118	96	91	88	99	.
2894	1	4/17/1989	322	F	Caucasian	.	94	79	81	87	78	.
2917	1	1/0/1900	323	F	Caucasian	Mouthwash only.	112	99	85	93	100	.
2907	1	8/18/1987	325	F	Unknown	.	107	93	96	96	89	.
3040	1	1/0/1900	327	F	Unknown	.	122	92	94	96	96	.

2914	1	9/16/1998	328	M	Unknown	.					
2910	1	7/7/1988	329	F	Unknown	.					
2958	1	1/0/1900	332	M	Caucasian	.	133	99	88	93	94	.
2959	1	5/7/1989	333	M	Caucasian	.	137	105	95	102	90	.
2977	1	11/14/1986	336	M	Caucasian	.	137	104	85	90	96	.
0	1	1/0/1900	338	F	Caucasian	.	107	90	80	93	84	.
3048	1	4/19/1986	339	F	Caucasian	.	115	99	86	101	100	.
3022	1	4/24/1989	340	F	Caucasian	.	107	96	87	93	83	.
3058	1	4/15/1989	342	M	Caucasian	.	110	92	88	87	61	.
3018	1	4/13/1984	343	M	Caucasian	.	110	89	98	77	73	.
3218	1	12/16/1989	346	F	Caucasian	.	115	83	82	81	85	.
3363	1	4/12/1992	348	M	Unknown	.					
3373	1	8/29/1989	349	F	Unknown	.	134	94	94	90	96	.
3356	1	12/13/1988	350	M	Unknown	.					
3404	1	12/4/1988	351	M	Unknown	.	112	82	80	80	88	.
3376	1	4/4/1989	352	M	Unknown	.	125	94	89	85	85	.
3383	1	10/18/1991	354	M	Unknown	.	104	89	92	97	93	.
3405	1	12/17/1990	356	F	Unknown	.	124	108	104	97	102	.
3393	1	6/1/1990	358	M	Unknown	.					
3431	1	6/9/1988	359	F	Unknown	.	110	89	97	97	96	.
3455	1	1/0/1900	362	M	Unknown	.	104	103	104	106	99	.
3418	1	2/18/1989	363	F	Unknown	.	105	97	97	90	86	.
3437	1	1/0/1900	364	M	Unknown	.	111	83	82	85	81	.
3465	1	1/0/1900	365	M	Unknown	.	106	87	80	63	74	.
3441	1	6/22/1992	366	F	Unknown	.	101	88	87	70	84	.
3486	1	5/11/1988	367	F	Unknown	.	123	94	82	83	90	.
3462	1	7/20/1989	368	M	Unknown	.	137	81	89	85	77	.
3518	1	7/12/1993	369	M	Unknown	.	111	75	85	77	69	.
3478	1	8/28/1989	370	F	Unknown	.	119	83	83	82	75	.
3669	1	10/9/1992	371	F	Unknown	.	105	80	86	84	84	.
3461	1	4/14/1990	372	M	Unknown	.	133	93	86	87	94	.
3489	1	10/24/1989	373	M	Unknown	.	106	63	67	77	73	.
3477	1	9/27/1988	374	F	Unknown	.	112	89	85	80	78	.
3467	1	10/26/1989	375	F	Unknown	.	94	82	76	90	82	.
3469	1	2/10/1989	376	M	Unknown	.	118	96	89	88	84	.
3470	1	12/15/1989	377	F	Unknown	.	118	95	94	82	85	.
3482	1	8/29/1988	378	M	Unknown	.	100	55	80	73	71	.
3514	1	5/20/1992	379	M	Unknown	.	117	83	80	87	84	.
3481	1	12/17/1988	380	M	Unknown	.	105	78	80	85	72	.
**3521**	**1**	**10/15/1991**	**381**	**F**	**Unknown**	**.**	**113**	**90**	**103**	**94**	**86**	**.**

3526	1	3/27/1991	382	F	Unknown	.	95	79	79	90	78	.
3536	1	9/25/1990	384	M	Unknown	.	106	70	69	70	69	.
3556	1	6/11/1993	385	M	Unknown	Proband does not qualify. Use family for LLD study.						
3583	1	6/16/1986	386	M	Unknown	.	100	73	69	71	69	.
3606	1	3/6/1987	387	M	Unknown	.	100	78	76	76	76	.
3577	1	5/25/1992	389	M	Unknown	.	104	97	94	94	94	.
3633	1	12/13/1993	390	F	Unknown	.	117	62	70	70	65	.
3650	1	3/19/1988	391	F	Unknown	.	98	82	79	82	.	.
3656	1	10/21/1989	392	F	Unknown	.						
3632	1	4/20/1985	393	M	Unknown	.	90	81	97	84	75	.
3655	1	1/0/1900	394	M	Unknown	.	104	81	68	76	76	.
3652	1	3/19/1988	395	M	Unknown	.	95	64	72	68	68	.
3657	1	3/29/1985	396	M	Unknown	.	94	92	89	75	66	.
3653	1	12/3/1991	397	M	Unknown	.	128	84	87	90	83	.
3658	1	8/17/1989	398	M	Unknown	.	98	74	71	78	74	.
3665	1	1/18/1989	399	M	Unknown	.	104	81	90	73	66	.
3748	1	3/19/1989	401	F	Unknown	.	113	75	69	80	72	.
3718	1	8/2/1993	402	M	Unknown	.	115	98	103	90	98	.
3781	1	5/20/1990	403	F	Unknown	.	100	90	86	88	82	.
3779	1	4/21/1988	404	F	Unknown	.	111	93	95	78	90	.
3774	1	2/3/1989	405	M	Unknown	.	119	103	90	77	99	.
3775	1	1/2/1989	406	M	Unknown	.	139	108	90	93	80	.
3802	1	6/5/1989	407	M	Unknown	.	94	96	103	78	83	.
3817	1	2/23/1989	410	F	Unknown	.	141	103	89	100	105	.
3819	1	2/28/1989	412	M	Unknown	.	124	99	93	105	110	.

Table S4B. Characteristics of dyslexia cases recruited from Atlanta

Sample code	Family ID	Phenotype	Relationship	Gender	Age
GFID_1	7	no	Mother	F	44
GFID_101	19	dyslexic	Proband	F	10
GFID_102	19	no	Father	M	48
GFID_103	19	no	Mother	F	48
GFID_104	19	difficulty spelling	Sister	F	8
GFID_106	5	no	Father	M	45
GFID_107	5	dyslexic	Mother	F	44
GFID_11	13	ND	Maternal Grandmother	F	72
GFID_113	5	dyslexic	Proband	F	11
GFID_114	5	dyslexic	Brother	M	7
GFID_117	23	dyslexic	Father	M	48
GFID_118	23	dyslexic	Brother	M	12

GFID_12	13	difficulty spelling	Maternal Grandfather	M	74
GFID_120	23	no	Brother	M	9
GFID_126	20	dyslexic	Proband	M	10
GFID_128	20	no	Father	M	44
GFID_129	20	no	Mother	F	43
GFID_131	20	no	Sister	F	13
GFID_132	20	dyslexic	Sister	F	16
GFID_134	28	dyslexic	Proband	M	8
GFID_135	28	no	Father	M	53
GFID_136	4	no	Mother	F	49
GFID_137	4	no	Father	M	50
GFID_138	4	no	Sister	F	14
GFID_139	4	dyslexic	Proband	F	11
GFID_140	28	no	Brother	M	6
GFID_145	4	difficulty spelling	Sister	F	18
GFID_147	4	no	Brother	M	16
GFID_148	28	dyslexic	Mother	F	40
GFID_149	28	no	Brother	M	3
GFID_15	12	difficulty spelling	Paternal Grandmother	F	68
GFID_157	27	difficulty spelling	Father	M	44
GFID_158	27	dyslexic	Maternal Grandmother	F	70
GFID_16	12	no	Paternal Grandfather	M	68
GFID_160	17	no	Mother	F	43
GFID_161	17	no	Sister	F	13
GFID_162	17	no	Brother	M	6
GFID_164	17	dyslexic	Brother	M	8
GFID_17	26	no	Paternal Grandfather	M	76
GFID_170	27	dyslexic	Mother	F	47
GFID_171	27	no	Paternal Grandmother	F	79
GFID_172	27	no	Paternal Grandfather	M	79
GFID_187	26	dyslexic	Aunt	F	53
GFID_194	26	no	Mother	F	41
GFID_195	26	dyslexic	Proband	M	9
GFID_2	7	no	Brother	M	10
GFID_20	7	no	Paternal Grandfather	M	71
GFID_203	1	dyslexic	Proband	F	10
GFID_204	1	no	Mother	F	43
GFID_209	16	no	Sister	F	10
GFID_21	6	ND	Paternal Grandmother	F	74
GFID_210	16	no	Father	M	50
GFID_211	29	no	Father	M	44
GFID_212	29	dyslexic	Proband	M	11
GFID_221	24	no	Brother	M	11
GFID_224	24	dyslexic	Proband	M	9
GFID_230	29	no	Mother	F	40
GFID_238	9	dyslexic	Proband	M	11
GFID_241	9	no	Father	M	42
GFID_242	9	no	Mother	F	40
GFID_243	9	no	Maternal Grandmother	F	61
GFID_244	9	no	Sister	F	7

GFID_245	6	no	Mother	F	43
GFID_248	6	no	Father	M	41
GFID_25	26	no	Paternal Grandmother	F	68
GFID_300	ND	dyslexic	Proband	ND	ND
GFID_301	ND	dyslexic	proband	ND	ND
GFID_302	ND	dyslexic	proband	ND	ND
GFID_303	ND	dyslexic	proband	ND	ND
GFID_304	ND	dyslexic	proband	ND	ND
GFID_305	ND	dyslexic	proband	ND	ND
GFID_306	ND	dyslexic	proband	ND	ND
GFID_307	ND	dyslexic	proband	ND	ND
GFID_308	ND	dyslexic	proband	ND	ND
GFID_309	ND	dyslexic	proband	ND	ND
GFID_310	ND	dyslexic	proband	ND	ND
GFID_311	ND	dyslexic	proband	ND	ND
GFID_312	ND	dyslexic	proband	ND	ND
GFID_313	ND	dyslexic	proband	ND	ND
GFID_314	ND	dyslexic	proband	ND	ND
GFID_315	ND	dyslexic	proband	ND	ND
GFID_316	ND	dyslexic	proband	ND	ND
GFID_317	ND	dyslexic	proband	ND	ND
GFID_318	ND	dyslexic	proband	ND	ND
GFID_319	ND	dyslexic	proband	ND	ND
GFID_320	ND	dyslexic	proband	ND	ND
GFID_321	ND	dyslexic	proband	ND	ND
GFID_322	ND	dyslexic	proband	ND	ND
GFID_323	ND	dyslexic	proband	ND	ND
GFID_324	ND	dyslexic	proband	ND	ND
GFID_325	ND	dyslexic	proband	ND	ND
GFID_329	ND	dyslexic	proband	ND	ND
GFID_330	ND	dyslexic	proband	ND	ND
GFID_334	ND	dyslexic	proband	ND	ND
GFID_335	ND	dyslexic	proband	ND	ND
GFID_336	ND	dyslexic	proband	ND	ND
GFID_337	ND	dyslexic	proband	ND	ND
GFID_34	29	no	Grandmother	F	75
GFID_340	ND	dyslexic	proband	ND	ND
GFID_341	ND	dyslexic	proband	ND	ND
GFID_342	ND	dyslexic	proband	ND	ND
GFID_343	ND	dyslexic	proband	ND	ND
GFID_344	ND	dyslexic	proband	ND	ND
GFID_345	ND	dyslexic	proband	ND	ND
GFID_346	ND	dyslexic	proband	ND	ND
GFID_347	ND	dyslexic	proband	ND	ND
GFID_348	ND	dyslexic	proband	ND	ND
GFID_349	ND	dyslexic	proband	ND	ND
GFID_35	29	no	Grandfather	M	77
GFID_350	ND	dyslexic	proband	ND	ND
GFID_351	ND	dyslexic	proband	ND	ND
GFID_352	ND	dyslexic	proband	ND	ND

GFID_353	ND	dyslexic	proband	ND	ND
GFID_354	ND	dyslexic	proband	ND	ND
GFID_355	ND	dyslexic	proband	ND	ND
GFID_356	ND	dyslexic	proband	ND	ND
GFID_357	ND	dyslexic	proband	ND	ND
GFID_358	ND	dyslexic	proband	ND	ND
GFID_359	ND	dyslexic	proband	ND	ND
GFID_36	18	no	Maternal Grandmother	F	74
GFID_360	ND	dyslexic	proband	ND	ND
GFID_361	ND	dyslexic	proband	ND	ND
GFID_362	ND	dyslexic	proband	ND	ND
GFID_363	ND	dyslexic	proband	ND	ND
GFID_364	ND	dyslexic	proband	ND	ND
GFID_365	ND	dyslexic	proband	ND	ND
GFID_366	ND	dyslexic	proband	ND	ND
GFID_367	ND	dyslexic	proband	ND	ND
GFID_368	ND	dyslexic	proband	ND	ND
GFID_369	ND	dyslexic	proband	ND	ND
GFID_37	18	no	Sister	F	18
GFID_370	ND	dyslexic	proband	ND	ND
GFID_371	ND	dyslexic	proband	ND	ND
GFID_372	ND	dyslexic	proband	ND	ND
GFID_373	ND	dyslexic	proband	ND	ND
GFID_374	ND	dyslexic	proband	ND	ND
GFID_375	ND	dyslexic	proband	ND	ND
GFID_376	ND	dyslexic	proband	ND	ND
GFID_377	ND	dyslexic	proband	ND	ND
GFID_378	ND	dyslexic	proband	ND	ND
GFID_379	ND	dyslexic	proband	ND	ND
GFID_38	25	no	Maternal Grandfather	M	69
GFID_380	ND	dyslexic	proband	ND	ND
GFID_381	ND	dyslexic	proband	ND	ND
GFID_382	ND	dyslexic	proband	ND	ND
GFID_383	ND	dyslexic	proband	ND	ND
GFID_384	ND	dyslexic	proband	ND	ND
GFID_385	ND	dyslexic	proband	ND	ND
GFID_386	ND	dyslexic	proband	ND	ND
GFID_387	ND	dyslexic	proband	ND	ND
GFID_388	ND	dyslexic	proband	ND	ND
GFID_389	ND	dyslexic	proband	ND	ND
GFID_39	25	no	Maternal Grandmother	F	67
GFID_390	ND	dyslexic	proband	ND	ND
GFID_392	ND	dyslexic	proband	ND	ND
GFID_393	ND	dyslexic	proband	ND	ND
GFID_394	ND	dyslexic	proband	ND	ND
GFID_395	ND	dyslexic	proband	ND	ND
GFID_396	ND	dyslexic	proband	ND	ND
GFID_397	ND	dyslexic	proband	ND	ND
GFID_398	ND	dyslexic	proband	ND	ND
GFID_399	ND	dyslexic	proband	ND	ND

GFID_4	7	dyslexic	Father	M	44
GFID_40	1	no	Maternal Grandmother	F	73
GFID_400	ND	dyslexic	proband	ND	ND
GFID_401	ND	dyslexic	proband	ND	ND
GFID_402	ND	dyslexic	proband	ND	ND
GFID_403	ND	dyslexic	proband	ND	ND
GFID_404	ND	dyslexic	proband	ND	ND
GFID_405	ND	dyslexic	proband	ND	ND
GFID_406	ND	dyslexic	proband	ND	ND
GFID_407	ND	dyslexic	proband	ND	ND
GFID_408	ND	dyslexic	proband	ND	ND
GFID_409	ND	dyslexic	proband	ND	ND
GFID_41	1	no	Maternal Grandfather	M	83
GFID_410	ND	dyslexic	proband	ND	ND
GFID_411	ND	dyslexic	proband	ND	ND
GFID_412	ND	dyslexic	proband	ND	ND
GFID_413	ND	dyslexic	proband	ND	ND
GFID_414	ND	dyslexic	proband	ND	ND
GFID_415	ND	dyslexic	proband	ND	ND
GFID_416	ND	dyslexic	proband	ND	ND
GFID_417	ND	dyslexic	proband	ND	ND
GFID_418	ND	dyslexic	proband	ND	ND
GFID_419	ND	dyslexic	proband	ND	ND
GFID_42	16	no	Maternal Grandmother	F	64
GFID_420	ND	dyslexic	proband	ND	ND
GFID_421	ND	dyslexic	proband	ND	ND
GFID_422	ND	dyslexic	proband	ND	ND
GFID_423	ND	dyslexic	proband	ND	ND
GFID_424	ND	dyslexic	proband	ND	ND
GFID_425	ND	dyslexic	proband	ND	ND
GFID_426	ND	dyslexic	proband	ND	ND
GFID_427	ND	dyslexic	proband	ND	ND
GFID_428	ND	dyslexic	proband	ND	ND
GFID_429	ND	dyslexic	proband	ND	ND
GFID_43	16	no	Maternal Grandfather	M	66
GFID_430	ND	dyslexic	proband	ND	ND
GFID_431	ND	dyslexic	proband	ND	ND
GFID_432	ND	dyslexic	proband	ND	ND
GFID_433	ND	dyslexic	proband	ND	ND
GFID_434	ND	dyslexic	proband	ND	ND
GFID_435	ND	dyslexic	proband	ND	ND
GFID_436	ND	dyslexic	proband	ND	ND
GFID_437	ND	dyslexic	proband	ND	ND
GFID_438	ND	dyslexic	proband	ND	ND
GFID_439	ND	dyslexic	proband	ND	ND
GFID_44	22	no	Paternal Grandfather	M	71
GFID_440	ND	dyslexic	proband	ND	ND
GFID_441	ND	dyslexic	proband	ND	ND
GFID_442	ND	dyslexic	proband	ND	ND
GFID_443	ND	dyslexic	proband	ND	ND

GFID_444	ND	dyslexic	proband	ND	ND
GFID_445	ND	dyslexic	proband	ND	ND
GFID_446	ND	dyslexic	proband	ND	ND
GFID_447	ND	dyslexic	proband	ND	ND
GFID_448	ND	dyslexic	proband	ND	ND
GFID_449	ND	dyslexic	proband	ND	ND
GFID_45	22	no	Paternal Grandmother	F	66
GFID_450	ND	dyslexic	proband	ND	ND
GFID_451	ND	dyslexic	proband	ND	ND
GFID_452	ND	dyslexic	proband	ND	ND
GFID_453	ND	dyslexic	proband	ND	ND
GFID_454	ND	dyslexic	proband	ND	ND
GFID_455	ND	dyslexic	proband	ND	ND
GFID_456	ND	dyslexic	proband	ND	ND
GFID_457	ND	dyslexic	proband	ND	ND
GFID_458	ND	dyslexic	proband	ND	ND
GFID_459	ND	dyslexic	proband	ND	ND
GFID_460	ND	dyslexic	proband	ND	ND
GFID_461	ND	dyslexic	proband	ND	ND
GFID_462	ND	dyslexic	proband	ND	ND
GFID_463	ND	dyslexic	proband	ND	ND
GFID_464	ND	dyslexic	proband	ND	ND
GFID_465	ND	dyslexic	proband	ND	ND
GFID_466	ND	dyslexic	proband	ND	ND
GFID_467	ND	dyslexic	proband	ND	ND
GFID_468	ND	dyslexic	proband	ND	ND
GFID_469	ND	dyslexic	proband	ND	ND
GFID_470	ND	dyslexic	proband	ND	ND
GFID_471	ND	dyslexic	proband	ND	ND
GFID_472	ND	dyslexic	proband	ND	ND
GFID_473	ND	dyslexic	proband	ND	ND
GFID_474	ND	dyslexic	proband	ND	ND
GFID_5	3	ND	Maternal Grandfather	M	71
GFID_68	15	dyslexic	Proband	M	9
GFID_69	15	no	Mother	F	41
GFID_7	2	no	Maternal Grandmother	F	60
GFID_70	15	no	Sister	F	11
GFID_71	15	no	Father	M	39
GFID_92	21	ND	Brother	M	9
GFID_93	21	ND	Sister	F	5
GFID_95	21	dyslexic	Proband	F	11
GFID_96	21	no	Mother	F	42
Dyslexic	189				
relative not dyslexic	38				
relative difficulty spelling	5				
relative don't know	2				
Total samples	234				
