# Supplementary material for: Relative Burden of Large CNVs on a Range of Neurodevelopmental Phenotypes
Source: PLoS Genet. 2011 Nov 10;7(11):e1002334. doi: 10.1371/journal.pgen.1002334 (PMC3213131; doi:10.1371/journal.pgen.1002334)
Supplement: Table S5 — Clinical details of cases with autism (both with and without ID). Clinical features of individuals recruited through the Simons Simplex Collection (n = 350) are shown. (PDF) [file pgen.1002334.s012.pdf]

Table S5. Clinical details of cases with autism (both with and without ID)

Sample ID	ID group	Sex	Age (years)	Age (m)	Ethnicity	ADOS score	Verbal IQ score	Non-verbal IQ score	Full scale IQ	Vineland composite score
Si31	profound	male	18	10	Caucasian	9	19	19	18	56
Si341	profound	female	17	7	Caucasian	7	18	26	23	45
Si86	severe	female	17	12		10	19	29	26	53
Si176	severe	male	14	10		9	20	29	26	52
Si98	severe	male	14	13	Caucasian	7	19	32	28	50
Si159	severe	female	14	17		6	21	31	28	55
Si151	severe	female	8	8	Caucasian	7	18	42	30	50
Si260	severe	female	14	8	Hispanic	10	21	34	30	43
Si312	severe	female	16	8	Caucasian	10	31	30	30	63
Si344	severe	male	15	10	Caucasian	6	28	33	31	52
Si305	severe	male	7	12		8	22	40	31	70
Si62	severe	male	10	14	Caucasian	8	15	49	32	60
Si242	severe	male	15	11		7	26	35	32	57
Si254	severe	female	12	17	Caucasian	6	27	34	32	54
Si164	severe	male	8	11	Asian	9	22	43	33	59
Si306	severe	male	14	14	Caucasian	6	26	36	33	59
Si4	severe	male	13	12	Caucasian	6	24	39	34	54
Si197	severe	male	15	13	Caucasian	9	35	34	34	57
Si253	severe	male	12	13		6	30	36	34	64
Si282	severe	male	10	9	Caucasian	6	24	41	35	63
Si273	severe	male	14	17	Caucasian	7	26	39	35	54
Si91	severe	female	11	11	Caucasian	8	36	36	36	64
Si264	severe	male	8	14		7	32	40	36	51
Si48	severe	male	11	12	Caucasian	10	30	45	37	57
Si222	severe	female	16	12		10	19	46	37	45
Si14	severe	male	8	14		5	31	44	38	60
Si101	severe	male	7	11		7	30	48	39	66
Si195	severe	male	12	9		10	26	45	39	58
Si64	severe	female	10	6		6	47	41	40	70
Si115	severe	female	12	13	Asian	7	31	45	40	61
Si166	severe	female	11	17	Caucasian	8	30	45	40	67
Si83	moderate	male	12	12	Caucasian	10	40	41	41	64
Si204	moderate	male	9	12		7	43	44	42	67
Si225	moderate	female	11	7	Caucasian	8	30	49	42	69
Si89	moderate	male	17	15	Asian	10	32	62	43	60
Si100	moderate	male	17	13	Caucasian	7	33	63	43	83
Si329	moderate	male	15	13	Caucasian	8	27	59	43	66
Si163	moderate	male	8	15	Caucasian	7	47	49	46	63
Si227	moderate	male	7	17		8	38	57	46	63

Si53	moderate	male	12	9	Caucasian	10	40	50	47	65
Si162	moderate	male	7	11		7	38	56	47	64
Si250	moderate	male	15	15	African-American	6	60	39	47	66
Si301	moderate	male	6	12		6	32	49	47	59
Si133	moderate	female	12	6	African-American	9	39	59	48	67
Si145	moderate	male	15	13	Caucasian	9	37	68	48	70
Si323	moderate	male	15	6		10	36	61	48	56
Si139	moderate	male	12	13		8	62	44	50	69
Si324	moderate	female	16	11	African-American	10	28	72	51	59
Si39	moderate	female	11	12	Caucasian	10	46	62	52	63
Si266	moderate	male	13	9	Caucasian	7	37	59	52	55
Si66	moderate	male	9	10	Caucasian	7	50	59	53	64
Si147	moderate	male	11	10		6	47	60	53	67
Si272	moderate	male	13	12		10	34	71	53	64
Si335	moderate	female	14	16		8	47	66	53	61
Si200	moderate	male	10	8	Caucasian	8	44	63	53	91
Si201	moderate	male	8	10	Caucasian	10	47	61	53	64
Si156	moderate	female	10	12		5	62	55	54	67
Si321	moderate	male	8	9	Caucasian	10	60	59	54	67
Si34	moderate	male	10	6		10	63	55	55	74
Si137	moderate	male	13	6	African-American	10	39	71	55	74
Si277	moderate	male	14	16		6	30	76	55	61
Si96	mild	male	15	13	Hispanic	9	58	55	56	58
Si84	mild	male	14	7	Caucasian	8	53	67	56	77
Si298	mild	male	18	12	Caucasian	8	23	82	56	48
Si287	mild	male	9	8		8	40	74	57	72
Si309	mild	male	8	15	Caucasian	9	51	65	57	65
Si169	mild	female	9	10	Caucasian	10	65	60	57	57
Si347	mild	female	8	7		7	75	55	58	72
Si241	mild	male	13	9		8	67	56	59	61
Si229	mild	female	11	16	Caucasian	9	45	67	59	69
Si245	mild	male	10	16		6	55	67	59	72
Si74	mild	male	13	8		9	51	66	60	60
Si81	mild	male	7	7	Caucasian	8	38	73	61	70
Si129	mild	male	6	7	Asian	9	58	64	61	77
Si333	mild	male	13	13		7	51	67	61	65
Si11	mild	female	10	6		7	67	65	62	77
Si178	mild	male	7	16	Caucasian	9	64	67	62	70
Si230	mild	male	18	17			70	60	63	75
Si303	mild	male	11	17	Caucasian	10	56	68	63	76

Si310	mild	male	10	9	Caucasian	9	61	70	63	66
Si41	mild	female	10	15	Caucasian	10	83	54	64	70
Si134	mild	male	10	7		6	49	79	64	71
Si171	mild	female	17	9		10	63	66	64	67
Si251	mild	male	16	12		10	31	89	64	59
Si109	mild	male	8	8	Caucasian	6	79	64	65	62
Si138	mild	male	9	10	Hispanic	9	59	74	65	69
Si325	mild	male	9	10	Caucasian	8	58	75	66	77
Si36	mild	male	9	8		6	40	87	67	66
Si130	mild	male	11	9		6	54	74	67	68
Si118	mild	male	11	15	African-American	8	56	76	68	63
Si238	mild	male	10	13		8	63	76	68	71
Si285	mild	male	12	8	Caucasian	9	57	77	68	87
Si340	mild	male	11	6	Caucasian	10	51	85	68	73
Si297	mild	male	7	13	Caucasian	8	79	62	69	74
Si261	mild	male	11	9		8	67	73	69	80
Si311	mild	male	7	13		8	59	59	69	67
Si6	mild	male	7	12	Asian	8	62	79	70	79
Si203	no MR	male	7	9	Asian	7	73	75	71	79
Si52	no MR	male	7	14	Caucasian	9	76	75	72	74
Si76	no MR	male	12	7		10	65	79	72	90
Si120	no MR	female	7	6	Caucasian	10	76	75	72	63
Si106	no MR	male	17	10		9	52	88	73	74
Si123	no MR	male	11	15	Caucasian	7	87	68	73	74
Si47	no MR	male	7	13		7	56	89	74	69
Si258	no MR	male	12	10		6	80	74	74	69
Si173	no MR	male	10	14	Caucasian	7	67	82	74	75
Si131	no MR	male	10	7	Caucasian	10	82	74	75	72
Si140	no MR	male	12	13	Caucasian	10	84	71	75	64
Si161	no MR	female	17	14		6	80	74	75	61
Si257	no MR	male	17	8		7	48	94	75	63
Si302	no MR	male	16	12	Caucasian	9	71	82	75	47
Si44	no MR	male	19	17	Hispanic		93	67	76	76
Si247	no MR	male	13	6		6	46	93	76	57
Si304	no MR	male	12	13	Hispanic	7	48	94	76	68
Si7	no MR	male	17	15			88	73	77	67
Si40	no MR	male	16	11		7	82	77	77	78
Si141	no MR	female	10	15		6	77	80	77	85
Si232	no MR	male	7	16	Caucasian	8	88	71	77	72
Si243	no MR	male	12	8		10	83	96	77	68
Si286	no MR	male	12	11		8	48	91	77	73
Si28	no MR	male	11	10	Caucasian	7	69	87	78	69

Si182	no MR	male	9	11	Caucasian	8	60	92	78	62
Si224	no MR	male	11	7	Caucasian	10	69	88	78	77
Si274	no MR	male	10	12		4	75	83	78	88
Si190	no MR	male	9	6	Caucasian	7	96	72	78	75
Si50	no MR	male	13	12		10	78	81	79	60
Si65	no MR	male	11	10		7	96	74	79	81
Si117	no MR	male	10	16	Caucasian	9	86	78	79	81
Si186	no MR	male	11	13	Hispanic	5	86	78	79	76
Si192	no MR	male	7	16		6	60	94	79	85
Si216	no MR	male	11	13		8	83	80	79	73
Si21	no MR	female	9	16	Asian	10	77	85	80	76
Si126	no MR	male	6	9	Caucasian	6	78	85	80	65
Si177	no MR	male	10	8	Caucasian	8	96	75	80	80
Si271	no MR	male	12	8	Caucasian	10	63	93	80	69
Si319	no MR	male	12	6		8	93	92	80	73
Si78	no MR	male	17	9	Caucasian	6	77	89	81	78
Si342	no MR	male	6	9		5	75	89	81	84
Si343	no MR	male	11	8		8	72	89	81	77
Si205	no MR	male	8	12	Caucasian	8	75	99	81	77
Si13	no MR	male	11	6		6	78	87	82	73
Si24	no MR	female	14	14	Caucasian	10	102	74	82	87
Si113	no MR	female	8	8	Caucasian	6	81	84	82	94
Si280	no MR	male	15	12	Caucasian	6	84	83	82	67
Si5	no MR	male	8	13	Caucasian	8	92	81	83	63
Si79	no MR	male	16	13		8	77	83	83	68
Si127	no MR	male	7	11		8	77	90	83	75
Si318	no MR	male	12	11		7	89	80	83	68
Si2	no MR	female	11	7		8	95	81	84	79
Si181	no MR	male	11	7	Caucasian	10	75	92	84	70
Si221	no MR	male	10	16	Caucasian	6	76	91	84	92
Si269	no MR	male	8	13		7	89	85	84	70
Si9	no MR	male	9	12		4	64	100	85	78
Si25	no MR	male	13	9		7	90	84	85	61
Si68	no MR	male	16	14		6	80	90	85	82
Si152	no MR	male	12	17	u	9	80	94	85	69
Si281	no MR	male	10	16	Asian	6	89	84	85	69
Si345	no MR	male	13	8		9	86	87	85	73
Si210	no MR	male	16	10	Caucasian	8	95	87	85	69
Si217	no MR	male	10	7	Caucasian	7	82	90	85	86
Si288	no MR	male	12	6	u	9	93	83	85	82
Si346	no MR	male	9	7		10	77	92	85	78
Si56	no MR	male	15	11	Caucasian	7	106	77	86	64
Si111	no MR	male	8	12		9	75	97	86	88

Si136	no MR	male	8	8		6	75	95	86	77
Si146	no MR	male	18	10	African-American	8	73	95	86	74
Si214	no MR	male	8	11	Caucasian	8	99	82	86	81
Si75	no MR	male	10	6	Caucasian	5	84	90	87	81
Si187	no MR	male	9	16	Caucasian	8	74	105	87	76
Si326	no MR	male	11	6		9	96	85	87	93
Si3	no MR	male	8	15		10	80	96	88	110
Si55	no MR	male	18	9		8	86	92	88	64
Si348	no MR	male	17	13	Caucasian	10	50	104	88	67
Si212	no MR	male	8	9		10	81	96	88	72
Si276	no MR	male	10	7	Caucasian	8	79	98	88	79
Si158	no MR	male	11	15		10	83	94	89	72
Si289	no MR	male	9	11	ai	7	88	91	89	84
Si185	no MR	male	11	15		6	89	90	89	78
Si220	no MR	male	8	14	Caucasian	8	95	89	89	74
Si267	no MR	male	11	15		7	91	89	89	76
Si249	no MR	male	11	8	Caucasian	6	95	89	89	85
Si80	no MR	male	6	6	Caucasian	6	87	95	90	85
Si97	no MR	male	10	8	Caucasian	10	87	93	90	75
Si102	no MR	male	9	10		10	93	92	90	77
Si119	no MR	male	9	9	Caucasian	10	86	94	90	84
Si183	no MR	male	13	7	Asian	7	73	102	90	66
Si168	no MR	male	19	7	Caucasian		104	84	90	65
Si8	no MR	male	16	15		7	93	91	91	66
Si49	no MR	male	12	8	Caucasian	4	88	94	91	73
Si85	no MR	male	13	14		9	81	101	91	80
Si88	no MR	female	7	6	Caucasian	5	91	94	91	83
Si153	no MR	male	10	16		10	74	103	91	75
Si128	no MR	male	8	15		7	83	98	91	69
Si315	no MR	male	9	12		10	97	89	91	81
Si144	no MR	male	10	13		10	82	96	92	71
Si194	no MR	male	9	13	Caucasian	10	104	87	92	70
Si339	no MR	male	8	17	Caucasian	8	91	95	92	76
Si337	no MR	male	8	10	Caucasian	6	104	90	93	92
Si218	no MR	male	11	15	Caucasian	10	96	93	93	90
Si338	no MR	male	11	10	Caucasian	9	82	101	93	80
Si116	no MR	male	7	9	Caucasian	10	88	100	94	68
Si184	no MR	male	10	17		10	77	105	94	70
Si124	no MR	male	14	7	u	6	77	105	94	67
Si213	no MR	male	10	8	Caucasian	7	89	100	94	96
Si252	no MR	male	13	10	African-American	8	106	89	94	78

Si290	no MR	male	8	11	Caucasian	6	93	97	94	64
Si291	no MR	male	10	11	African-American	4	110	86	94	88
Si317	no MR	male	16	7		8	98	94	95	93
Si18	no MR	male	13	6	Hispanic	9	106	91	96	78
Si19	no MR	male	8	15	Caucasian	6	101	95	96	66
Si63	no MR	male	11	13	Caucasian	9	81	106	96	71
Si108	no MR	female	8	10	Caucasian	9	98	97	96	87
Si35	no MR	male	17	17		8	96	98	97	66
Si46	no MR	female	6	12		10	94	100	97	86
Si72	no MR	male	17	6		5	89	102	97	69
Si73	no MR	male	16	13	Caucasian	9	83	108	97	85
Si135	no MR	male	13	7		5	108	93	97	88
Si193	no MR	male	8	15		8	84	106	97	86
Si284	no MR	male	11	13	Caucasian	9	106	92	97	68
Si236	no MR	male	13	14	Caucasian	7	98	102	97	68
Si69	no MR	male	6	11		5	95	101	98	62
Si331	no MR	male	12	11		8	86	104	98	73
Si228	no MR	male	10	6	Caucasian	10	89	107	99	96
Si334	no MR	male	15	7		9	100	123	99	76
Si17	no MR	male	9	10	Caucasian	7	102	100	100	91
Si255	no MR	male	6	9		5	99	101	100	88
Si244	no MR	male	17	10	Caucasian	9	102	110	100	58
Si10	no MR	male	7	17	Caucasian	9	103	100	101	80
Si15	no MR	male	14	8	Caucasian	4	86	109	101	84
Si82	no MR	male	14	8		4	104	100	101	81
Si104	no MR	male	11	6	Caucasian	9	90	88	101	80
Si350	no MR	male	7	17	Caucasian	6	115	93	101	82
Si189	no MR	female	9	14	Hispanic	10	99	119	101	72
Si316	no MR	female	10	14	Caucasian	7	122	90	101	92
Si327	no MR	male	8	17		9	106	96	101	83
Si165	no MR	male	14	7		8	98	127	102	76
Si191	no MR	male	12	15		7	108	102	102	76
Si237	no MR	male	13	17	Hispanic	6	104	108	102	68
Si322	no MR	male	12	9		10	80	114	102	76
Si148	no MR	female	6	16	Caucasian	5	107	100	103	87
Si155	no MR	male	13	8	Caucasian	8	120	93	103	80
Si179	no MR	male	10	16	Caucasian	5	94	108	103	81
Si292	no MR	male	14	11		9	100	105	103	72
Si27	no MR	male	17	14	Caucasian	7	93	110	104	65
Si99	no MR	male	10	12	Caucasian	4	100	107	104	94
Si198	no MR	male	10	8	ai	7	105	103	104	103
Si233	no MR	male	10	16	Asian	7	85	114	104	78

Si299	no MR	male	9	7		9	102	104	104	76
Si307	no MR	male	15	13	Caucasian	9	96	111	104	70
Si320	no MR	male	9	11		7	110	101	104	90
Si270	no MR	male	12	12		6	96	109	104	76
Si16	no MR	male	11	6	Caucasian	10	108	100	105	66
Si32	no MR	male	7	8		6	85	115	105	100
Si175	no MR	female	16	10		5	105	105	105	72
Si174	no MR	male	9	14	Asian	7	104	104	105	82
Si12	no MR	male	7	12		8	99	108	106	87
Si30	no MR	male	11	10	Caucasian	8	113	102	106	71
Si110	no MR	male	6	6	Caucasian	6	97	111	106	88
Si122	no MR	female	8	12	Caucasian	6	122	95	106	80
Si328	no MR	male	10	16	Caucasian	8	86	129	106	84
Si226	no MR	male	12	9	Caucasian	4	91	112	106	77
Si43	no MR	male	9	14		10	114	104	107	93
Si95	no MR	male	8	15	Caucasian	5	98	112	107	73
Si142	no MR	male	6	10	Caucasian	4	117	99	107	90
Si157	no MR	male	12	14		4	119	100	107	78
Si199	no MR	male	9	9	Caucasian	6	101	109	107	
Si58	no MR	male	8	15		6	90	117	108	82
Si336	no MR	male	7	10	Hispanic	8	111	104	108	96
Si20	no MR	male	12	14	Caucasian	7	123	101	109	68
Si22	no MR	male	6	15	Caucasian	8	104	111	109	89
Si23	no MR	male	9	17	Caucasian	5	105	110	109	96
Si26	no MR	male	8	7	Caucasian	6	106	109	109	92
Si87	no MR	male	15	16	Caucasian	9	114	117	109	81
Si170	no MR	male	18	17	Caucasian		128	97	109	77
Si283	no MR	male	10	14	Caucasian	9	99	125	109	89
Si300	no MR	male	8	11		5	114	103	109	86
Si313	no MR	male	13	13	Caucasian	8	108	108	109	75
Si29	no MR	female	8	7	Caucasian	6	105	111	110	83
Si314	no MR	male	7	10	Caucasian	10	109	109	110	94
Si42	no MR	female	6	15		8	107	111	111	85
Si180	no MR	male	19	7	Caucasian		129	100	111	73
Si349	no MR	male	19	13			128	100	111	71
Si248	no MR	male	9	8		5	119	103	111	77
Si279	no MR	male	19	14	Caucasian		128	101	111	64
Si77	no MR	male	14	12		6	114	109	112	74
Si211	no MR	male	10	12	Caucasian	7	108	115	112	91
Si67	no MR	male	7	17	Caucasian	6	123	104	113	100
Si235	no MR	male	10	7	Caucasian	9	99	119	113	95
Si256	no MR	female	14	8		4	107	114	113	77
Si57	no MR	male	11	11	Caucasian	6	117	111	114	83

Si70	no MR	male	10	13	Caucasian	8	118	111	114	83
Si125	no MR	male	10	7	Caucasian	8	117	111	114	89
Si207	no MR	male	7	8		7	113	111	114	91
Si294	no MR	female	10	12		7	121	109	114	73
Si263	no MR	male	11	14		6	121	117	114	79
Si61	no MR	male	13	11		6	128	106	115	67
Si92	no MR	male	6	16	Caucasian	4	98	123	115	86
Si93	no MR	male	14	10	Caucasian	4	111	114	115	80
Si132	no MR	female	16	11			117	113	115	72
Si33	no MR	male	19	11			114	115	116	89
Si103	no MR	male	8	8	Caucasian	6	103	121	116	106
Si215	no MR	male	11	12	Hispanic	6	120	112	116	77
Si143	no MR	male	10	8		4	108	119	117	84
Si208	no MR	male	14	15		4	84	132	117	61
Si231	no MR	male	9	9	Caucasian	6	111	117	117	93
Si196	no MR	male	17	14			116	117	118	74
Si275	no MR	male	15	11		7	130	109	118	77
Si206	no MR	male	13	17	Caucasian	8	114	119	119	101
Si246	no MR	male	18	15			117	118	119	59
Si202	no MR	male	14	11	Caucasian	4	123	115	120	84
Si223	no MR	female	19	8			121	125	120	59
Si45	no MR	male	9	14		7	95	131	121	86
Si60	no MR	male	10	16	Caucasian	6	107	125	121	76
Si114	no MR	female	11	15	Caucasian	8	119	119	121	96
Si121	no MR	male	9	9	Caucasian	5	136	110	121	91
Si149	no MR	male	13	13		8	131	112	121	74
Si240	no MR	male	7	13		6	114	123	122	83
Si265	no MR	male	10	13		10	136	111	122	76
Si295	no MR	female	19	15	Hispanic		140	109	123	69
Si51	no MR	male	12	12		7	113	129	127	95
Si71	no MR	male	15	11	Caucasian	5	128	124	127	96
Si160	no MR	male	9	15		10	99	138	127	82
Si1	no MR	male	12	8	Caucasian	8	120	126	128	72
Si37	no MR	male	10	13	Caucasian	8	115	129	128	82
Si239	no MR	male	8	9		4	118	129	128	79
Si209	no MR	male	13	15	Caucasian	6	120	128	129	90
Si219	no MR	male	9	10	Caucasian	8	118	128	129	101
Si293	no MR	male	10	10		7	107	134	129	88
Si38	no MR	male	15	11		10	116	131	130	70
Si105	no MR	male	10	14		6	110	134	130	78
Si107	no MR	male	10	8		6	123	127	130	89
Si167	no MR	male	15	7	Caucasian	8	128	118	130	82
Si332	no MR	male	13	13	Caucasian	6	121	128	130	100

Si259	no MR	male	12	12		9	119	137	132	79
Si234	no MR	male	13	15		8	121	132	133	81
Si188	no MR	male	9	17	Caucasian	7	134	128	135	91
Si94	no MR	male	14	11	Caucasian	8	106	146	137	83
Si268	no MR	female	16	17			149	128	141	71
Si296	no MR	male	18	10			143	132	141	68
Si172	no MR	male	12	13	Caucasian	9	113	158	148	84
Si54	unknown	male	8	10						
Si59	unknown	female	7	17						
Si90	unknown	male	9	13						
Si112	moderate	male	11	17	Caucasian	10	40	77	~55	69
Si150	unknown	male	9	11						
Si154	unknown	male	7	11						
Si262	unknown	male	9	16						
Si278	unknown	male	9	17						
Si308	unknown	male	11	13						
Si330	unknown	male	12	6						
