# Supplementary material for: Relative Burden of Large CNVs on a Range of Neurodevelopmental Phenotypes
Source: PLoS Genet. 2011 Nov 10;7(11):e1002334. doi: 10.1371/journal.pgen.1002334 (PMC3213131; doi:10.1371/journal.pgen.1002334)
Supplement: Table S6 — Clinical details of cases with idiopathic ID. Clinical features of individuals with ID are shown. These individuals were recruited and evaluated at the IRCCS Associazione Oasi Maria Santissima, Troina. (PDF) [file pgen.1002334.s013.pdf]

Table S6. Clinical details of cases with idiopathic ID

Patient Code	Sex	Age (years)	Phenotype	Other genetic tests
01012	M	20	Profound ID, Myoclonic epilepsy	
01019	F	27	Moderate ID, glycogenosis, microcephaly, ataxia, cerebellar hypoplasia and hypotrophy	
01113	M	34	Moderate ID, motor polyneuropathy, retinitis pigmentosa, CNS developmental defects	
01124	F	34	Profound ID, Drug-resistant epilepsy, Microcephaly, Spastic tetraplegia, Perinatal hypoxic ischemic encephalopathy	
01164	M	20	Severe ID, dysmorphic features	
01230	F		Mild ID, PDD, epilepsy	
01235	F	15	Severe ID, Microcephaly, Tetraplegia, Ataxia	
01236	M	24	Moderate ID, macrocephaly, myopathy	
01246	M	15	Borderline Intellectual Functioning, ASD	
01274	M	17	Severe ID, Saethre-Chotzen syndrome (ACS III)	
01314	M	15	Severe ID, Obesity	
01318	M	18	Severe ID, Macrocephaly, Ataxia, Dysmorphic features	
01357	M	24	Moderate ID, Saethre-Chotzen syndrome (ACS III)	
01398	M	14	Profound ID, Spastic Tetraparesis, Microcephaly, CNS Malformation	
01402	M	30	Moderate ID, Anxiety disorder	
01434	M			
01438	M	19	Moderate ID	
01447	M			
01509	M	27	Severe ID, ASD, Focal Epilepsy	
01512	M	15	Borderline Intellectual Functioning, Expressive speech impairment	
01522	M	38	Severe ID, Lujan-Fryns phenotype	
01538	M			
01540	F			
01575	M	26	Moderate ID, Psychosis	
01583	M	27	Mild ID, Neurosensory deafness, Hypoxic Ischemic Encephalopathy	

01593	F	22	Severe ID, Spastic Paraparesis, Saethre-Chotzen syndrome, Generalized Developmental Disorder	
01609	M	17		
01613	F	13	Moderate ID, Spastic Tetraparesis, Hypoxic Ischemic Encephalopathy	
01616	M	16	Severe ID, Pervasive Developmental Disorder	
01619	F	14	Severe ID, Partial Occipital Epilepsy	
01631	M	14	Severe ID, ASD, Hypospadia, Cryptorchidism	
01634	M	21	Profound ID, ASD	
01658	F	51	Moderate ID, Cerebral Atrophy	
01686	F	16	Severe ID, Microcephaly, IUGR	
01705	M	22	Severe MCA/ID, PDD, Epilepsy	
01728	M	24	Moderate ID, Obesity, Astigmatism	
01746	M	19	Moderate MCA/ID, Partial Epilepsy	
01755	M	12	Mild ID, Craniosynostosis	
01827	M	22	Moderate ID, Partial Epilepsy, Perinatal Hypoxic Ischemic Encephalopathy	
01833	F	11	Severe MCA/ID, Microcephaly, PDD	
01856	M	16	Severe ID, PDD	
01879	F	19	Severe ID, Partial Epilepsy	
01883	F	26	Borderline Intellectual Functioning, Psychosis, MCA	
01893	F	17	Mild ID	
01911	M	21	Moderate ID, Microcephaly, Partial Epilepsy	
01930	M	34	Probable Autosomal Dominant Cerebellar Ataxia	Frataxin
01959	M	42	Borderline Intellectual Functioning, Cerebellar Ataxia, Spastic Paraparesis, Polyneuropathy	
01977	F	19	Mild ID, Epilepsy, Congenital Cerebellar Ataxia, Left Parieto-Occipital Ischemic Lesion	
01996	M	13	Moderate ID, PDD	
02003	M	11	Severe ID	
0201	M	38	Severe ID, Lujan-Fryns phenotype	
02012	M	72	Mild ID, Cerebellar Ataxia, Neurosensory Deafness, Cerebral and	SCA

			Cerebellar Atrophy	
0203	F	21	Severe ID, Spastic Paraparesis, Motor Axonal Neuropathy	
02031	M	13	Severe ID, PDD	
02040	F	93	Cognitive Decline, Cortical Atrophy, Hypertension, Cronic Renal Failure	
02043	M	64	Cerebellar Ataxia	
02055	F	40	Severe ID, Spastic Tetraparesis, Ataxia, Epilepsy, Microcephaly, Sensory Motor Neuropathy, Cerebral and Cerebellar Atrophy	
02082	M	25	Mild ID, Saethre-Chotzen syndrome (ACS III)	TWIST gene
02087	M	18	Partial Epilepsy, Perinatal Hypoxic Ischemic Encephalopathy, Anxiety Disorder, Marfanoid Habitus	
02092	F	12	Moderate MCA/ID, karyotype 46,XX,15p+	Karyotype
02099	M	28	Severe ID	
02106	M	12	Moderate ID, Ectodermal Dysplasia NOS	
02110	M	38	Schyzoid Personality Disorder, Myopathy, Macrocephaly	
02117	M	26	Mild ID, PDD	
02120	M	33	Moderate ID, Mild cerebral and cerebellar vermis atrophy	
02129	M	15	NOS ID, Infantile Psychosis	
02139	M	20	Moderate ID, Microcephaly, Left Spastic Hemiparesis, Partial Epilepsy, Widespread Dysarrangement of Neuronal Migration and Cortical set-up	
02140	F	40	Profound ID, Spastic Tetraparesis, Microcephaly, CNS Malformation	
02147	M	9	ADHD, Craniosynostosis	
02154	F	10	Mild ID	
02159	M	13	Mild ID, Tetraplegia, Probable Hallervorden-Spatz disease	
02160	F	16	Moderate ID, Spastic Tetraparesis, Mitochondrial Encephalomyopathy with POLG1 gene Mutation	POLG1 gene
02174	F			
02175	F	11	Mild ID	
02182	F	17	Moderate ID, Arnold-Chiari I, Syringomielia, Psychosis	
02184	M	26	Borderline Intellectual Functioning, Anxiety Disorder. Crouzon Syndrome	
02185	F	10	NOS MCA/ID	
02186	M	13	Severe ID, Schimmelpenning-Feuerstein syndrome	

02200	M	65		
02701	M	Dead 9-year-old	Profound ID, CNS Malformation, Familiar Lissencephaly	
02222	M	9	Mild ID, ACS	MLPA Genes Craniosynostosis
02223	F	10	Mild ID	
02227	M	21	NOS ID, NOD PDD	TWIST gene, FGFR gene
02237	M	11	Miopathy	
02245	F	37	Profound ID, Mosaic Turner syndrome, Crouzon Craniosynostosis	Karyotype
02248	M	19	Profound ID, ASD, Microcephaly	
02257	F	17	Moderate ID	TWIST and FGFRs genes
02259	M	12	Borderline Intellectual Functioning, Microcephaly, ADHD, Miopathy	FRAXA, FRAXE
02304	M	8	NOS ID, Plagiocephaly, Brachycephaly	TWIST and FGFRs genes
0231	M	36	NOS ID, Schyzophrenia, Dysmorphic features	
02322	M	25	Severe ID, Miopathy, Dysmorphic features	
02325	M	16	Severe MCA/ID, Craniosynostosis	TWIST and FGFRs genes
02334	M	17	Moderate ID, PDD, Miopathy	POLG1 gene
02412	M	23	Severe ID, Psychosis	Subtelomeric FISH
02417	F	17	Moderate ID, PDD, Fetal Alcohol Syndrome	
02423	M	9		
02424	M		NOS ID	
02427	F	11		
02433	M	15	Mild ID, PDD, Partial Epilepsy, Left Hemihypertrophy	
02434	M	15	Moderate ID, Psychosis	
02437	F	26	Mild ID, Schizophrenia	
02438	F	16	Mild ID, Partial Epilepsy, Perinatal Hypoxic Ischemic Encephalopathy	
02442	M	17		
02443	F	23	Moderate ID, Epilepsy, Polimicrogiria, Schizencephaly	Karyotype, Subtelomeric MLPA
02447	F	27	Moderate ID	
02448	M	26	Moderate ID, Partial Epilepsy	

02449	M	34	Moderate ID, Spastic Tetraparesis, Perinatal Hypoxic Ischemic Encephalopathy	Karyotype, PQBP1 gene
02450	M	7	Right Hemiparesis	
02452	M	19	Moderate MCA/ID	
02453	M	28	Profound ID, AR Primary Microcephaly	
02455	M	10	Borderline Intellectual Functioning	
02456	F	9	NOS MCA/ID, PDD	Karyotype, Subtelomeric MLPA
02457	M	25	Moderate MCA/ID, Epilepsy	Subtelomeric MLPA
02458	M	18	Severe ID, ASD	Subtelomeric MLPA
02459	M	35	Mild ID, Microcephaly, Miopathy	
02461	M	11	NOS ID, PDD	
02462	M	14	ASD High Functioning	
02464	F	12	Mild ID, Epilepsy, Down Syndrome	Karyotype
02465	F	11	Moderate ID, Down Syndrome	Karyotype
02466	M	15	Mild ID, Psychosis, Obesity	
02467	M	18	ASD, Borderline Intellectual Functioning	
02470	M	9	Borderline Intellectual Functioning, Perthes Disease	
02471	F	13	Profound ID, Spastic Tetraparesis, Microcephaly, Motor peripheral neuropathy	
02473	F	15	Mild ID, Microcephaly, EEG anomalies	
02475	M	23	Mild ID, Anxiety Disorder	Karyotype, Subtelomeric MLPA
02477	F	10	Moderate ID	
02478	M	17	Borderline Intellectual Functioning, OCD, Partial Epilepsy	PTEN gene
02479	M	6	CHD, Normal Intellectual Functioning	Karyotype, FISH 22q11.23, Subtelomeric MLPA
02480	M	11	Severe ID, PDD	
02482	F	9	Down Syndrome, Moderate ID, Hypothyroidism	Karyotype
02485	F	17	Left Hemiparesis, Normal Intellectual Functioning	Karyotype, Subtelomeric MLPA
02487	M	35	Severe MCA/ID, EEG and EMG anomalies	Subtelomeric MLPA
02491	M	10	Expressive Speech Disorder	FISH 22q11.23, Subtelomeric MLPA

02492	M	8	Severe ID, 18p Tetrasomy	Karyotype
02493	M	19	NOS ID, ASD	Subtelomeric MLPA
02494	F	44	Profound MCA/ID	Karyotype, Subtelomeric MLPA
02495	M	29	Mild ID, Psychotic Disorder	PTEN gene
02496	M	9	Moderate ID, ASD	PTEN gene
02500	F	15	Mild ID, Early Onset Psychosis	Subtelomeric MLPA
02502	M	23	Severe ID, Triplication Critical Region 21q 22.13-q22.2	Karyotype, LSI21 FISH
02503	M	33	Borderline Intellectual Functioning, Partial Epilepsy	
02508	M	8	Mild MCA/ID	
02509	M	10	Mild ID, Dysmorphic features	Karyotype, Subtelomeric MLPA
02510	M	21	Mild ID	Karyotype, Subtelomeric MLPA
02511	F	11	Moderate MCA/ID, PDD	
02512	M	27	Profound ID. Partial 11p Trisomy	Karyotype
02513	M	22	Mild ID	Karyotype, Subtelomeric MLPA
02514	M	18	Moderate ID	Karyotype, Subtelomeric MLPA
02515	M	13	ADHD, BIF	Karyotype, Subtelomeric MLPA
02518	F	25	Mild ID	Karyotype, Subtelomeric MLPA
02520	M	9	Mild ID, ASD	Karyotype, Subtelomeric MLPA
02521	M	15	Learning Disability, Obesity	
02522	F	26	Severe ID, Partial Trisomy 15	Karyotype
02524	M	7	NOS ID, Spastic Tetraparesis	
02525	M	21	Mild ID	Karyotype, Subtelomeric MLPA
02526	M	16	Moderate ID, ASD	
02528	F	11	Moderate ID, OCD	
02529	M	18	Severe ID, ASD, EEG anomalies	Subtelomeric MLPA
02531	M	26	Moderate ID, OCD	Karyotype, Subtelomeric MLPA
02532	F	25	Mild ID, Psychosis, Neurosensory Deafness	Karyotype, Subtelomeric MLPA
02533	F	16	Mild ID, OCD	
02535	M	19	Moderate ID, ASD	Karyotype, Subtelomeric MLPA

02537	F	16	Moderate ID	
02538	F	16	Mild ID, ADHD	
02539	M	8	Severe ID, Epilepsy	
02542	F	28	Severe ID, Obesity	Subtelomeric MLPA
02543	F	19	Mild ID, Microcephaly, Spastic Tetraparesis	Subtelomeric MLPA
02546	M	9	Mild ID	Karyotype, Subtelomeric MLPA
02547	F	8	NOS ID	Karyotype, Subtelomeric MLPA
02548	F	10	Severe ID, PDD	
02550	M	21	BIF, Psychosis, EEG Anomalies	Karyotype, Subtelomeric MLPA, FraXE
02551	M	15	Mild ID, ADHD, Obesity	Karyotype, Subtelomeric MLPA
02553	F	34	ADCA2	SCA2
02554	F	46	SCA	SCA
02555	F	15	Mild MCA/ID, Paraparesis, Immune Dysfunction	PTEN gene
02557	M	11	Mild ID	Subtelomeric MLPA
02560	M	10	Mild ID, Diplegia, Microcephaly	Subtelomeric MLPA
02561	F	7	Aicardi syndrome, Infantile Spasms, Severe ID	
02562	F	17	Mild ID, Epilepsy, Spastic Diplegia	Kartotype, Subtelomeric MLPA
02563	M	12	Severe ID, ASD	
02566	M	36	Down syndrome, Severe ID, Epilepsy	Karyotype
02568	M	59	Narcolepsy	
02569	M	28	NOS ID, Psychosis, Macrocephaly, EEG anomalies	Karyotype, PTEN and FMR-1 genes
02571	M	14	Mild ID, PDD, Obesity	Subtelomeric MLPA, PTEN Gene
02572	M	15	BIF, PDD	Subtelomeric MLPA
02573	M	7	Mild ID	
02574	F	57	Ataxia	SCA
02575	F	23	BIF, Partial Epilepsy, Microcephaly	Karyotype
02576	F	18	Moderate ID	Subtelomeric MLPA
02577	M	13	BIF, ADHD	
02578	F	18	Mild ID, Early Onset Psychosis	Karyotype, FISH 22q11.23

02579	M	12	BIF, MCA	Subtelomeric MLPA. FISH 22q11.23
02580	M	12	BIF	Subtelomeric MLPA. FISH 22q11.23
02581	M	31	Moderate MCA/ID	PTEN gene
02582	M	11	NOS ID, ASD	Subtelomeric MLPA
02585	M	11	Mild ID, Left Hemiparesis	PQBP-1 gene
02586	M	12	NOS ID, PDD	Subtelomeric MLPA
02587	F	45	AR Microcephaly	
02589	M	10	Severe ID	
02590	F	22	NOS MCA/ID, Epilepsy	Karyotype
02592	F	12	NOS, MCA/ID, PDD	Subtelomeric MLPA, MECP-2
02593	M	16	Moderate ID, Myoclonic Epilepsy	
02595	F	7	NOS ID, Microcephaly, Tetraparesis	Subtelomeric MLPA
02598	F	12	Williams syndrome, Mild ID	FISH 7q11.23
02599	M	11	Severe MCA/ID	
02601	F	9	Ophthalmoplegia Cerebellar Hypoplasia	
02602	F	30	Severe ID, Epilepsy, Microcephaly	
02604	M	7	Mild ID	
02605	M	33	Profound MCA/ID, Tetraparesis, Partial Epilepsy	Karyotype
02609	M	48	Severe MCA/ID	Subtelomeric MLPA
02610	F	20	Moderate ID, ASD	Subtelomeric MLPA
02612	M	7	Severe ID, Epilepsy	Subtelomeric MLPA, ARX gene, Mitocondrial DNA screening
02615	M	11	BIF, PDD, Craniosynostosis	Karyotype, TWIST ans FGFRs genes, Subtelomeric MLPA
02620	M	16	BIF, Early Onset Psychosis, Macrocephaly	Karyotype, PTEN gene
02623	F	17		
02624	F	9	BIF, Trigonocephaly	TWIST and FGFRs genes
02627	M	8	Mild ID, PDD	Subtelomeric MLPA
02628	M	66	Spastic Tetraparesis, Medullary Focal Atrophy by Ischemic lesion	
02630	F	9	MCA, Cytomegalovirus Connatal Infection	

02635	F	49	Severe ID, Epilepsy, ASD	
02636	M	26	Severe ID, ASD	
02637	M	35	Profound MCA/ID, Epilepsy, Immune Deficiency	
02638	M	7	NOS ID, Unbalanced 4-15 translocation 4q35 and 15 PTER®q12 deletions. Phenotype compatible with Angelman syndrome	Karyotype, FISH
02639	M	18	Severe MCA/ID	Karyotype, Subtelomeric MLPA, FMR-1
02641	M	8	Expressive speech delay, Dolichocephaly	Karyotype, Subtelomeric MLPA
02642	M	22	Profound ID, Epilepsy, Spastic Tetraparesis, Perinatal Hypoxic Ischemic Encephalopathy	ARX gene
02643	M	20	Severe ID, ASD, Right Neurosensory Deafness	
02644	F	10	Mild ID, Brachycephaly	
02645	M	8	Severe ID, Spastic Tetraparesis, Partial Epilepsy	Subtelomeric MLPA
02653	M	13	Moderate ID, Psychosis	
02656	M	14	Severe ID, PDD	Subtelomeric MLPA
02770	M	10	Mild ID	
02853	F	14	Moderate ID, Generalized Epilepsy	
03058	M	14	Moderate ID, Hyperactivity	
03182	M	15	Mild ID, OCD	
03183	F	33	NOS ID	Karyotype
03184	M	45	Severe MCA/ID, Psychosis	
03185	M	9		
03187	M	8	Mild MCA/ID, NOS Nuromuscular disease	
03188	F	13	NOS ID, Tetraparesis, Microcephaly	
03189	M	9	Mild ID	
03201	M	17	Severe ID, PDD, Left Neurosensort Deafness	
03202	F	22	Severe MCA/ID, Partial Epilepsy	
03203	M	9	Moderate MCA/ID, IUGR	
03204	M	7	NOS ID, EEG Anomalies	
03205	M	5	NOS ID, Plagiocephaly	Subtelomeric MLPA
03207	M	13	Mild ID, Obesity	

03208	M	14	BIF, Microcephaly	
03210	M	10	Mild ID	
03247	F	20	Mild MCA/ID, OCD	
03249	F	12	Moderate ID	
03250	F	21	Mild MCA/ID, Microcephaly, Short Stature	
03252	M	8	Normal Intellectual Functioning	
03256	M	11	BIF, PDD	
03258	M	12	Mild ID, ADHD	
03259	M	14	BIF, PDD, EEG Anomalies	
03266	M	7	NOS ID, ASD	
03268	M	8	Moderate ID, PDD, Rubinstein Taybi phenotype	
03269	M	17	Moderate ID	
03270	F	8	Moderate MCA/ID	
03271	M	22	Moderate ID, Ataxia	SCA genes. Mitochondrial Genes Mutation Screening
03272	M	7	NOS ID, PDD	
03275	M	8	Expressive Speech Disorder	
03276	M	14	Mild ID, Microcephaly	
03279	M	21	Moderate ID, Compulsive Behaviour	
03280	M	9	Gait Disturbance, First Complex Mitochondrial Disease	
03282	F	11	Severe MCA/ID	
03284	M	14	Severe ID, ASD	
03285	M	14	Mild ID	
03286	M	23	Mild ID	
03287	M	7	Severe ID, PDD	
03288	F	7	NOS ID	
03289	M	10	Mild ID, Chiari 1 Anomaly	
03290	M	13	Learning Disability	
03291	F	62		

03292	M	18	Mild ID	
03293	F	13	Mild MCA/ID	
03294	F	14	Mild ID	
03295	F	27	Severe ID, Generalized Epilepsy	PTEN gene
03296	M	27	Moderate ID, Partial Epilepsy	
03297	F	46	Spastic Paraparesis, Motor Axonal Neuropathy	
03298	M	15	BIF, Epilepsy	
03299	M	16	NOS MCA/ID	
03300	M	5	NOS ID, Macrocephaly	
03301	M	6	Moderate ID, Myoclonic Epilepsy	
03302	M	14	Moderate MCA/ID	
03303	M	11	BIF	
03305	M	8	BIF	
03306	F	3	Profound ID, Microcephaly, Epilepsy	
03307	F	55	Ataxia, Tetraparesis, Epilepsy	SCA genes
03308	M	19	Profound MCA/ID, Partial Epilepsy	
03311	M	6	Severe ID, PDD	
03312	M	5	Expressive Speech Disorder, Epilepsy, Macrocephaly, External Hydrocephalus	
03313	M	19	Severe ID, PDD	
03314	M	11	Moderate ID, Epilepsy	
03315	F	26	Profound ID, Epilepsy	MECP2
03316	F	28	Severe ID, Microcephaly	
03317	F	51	SCA	
03319	M	14	Moderate ID, Neurosensory Deafness, Myopathy, COX deficit	
03320	M	14	Mild ID, ADHD, Epilepsy	
03321	M	28	Severe ID, PDD, Epilepsy	
03322	M	16	BIF	
03323	F	9	Mild ID	

03324	F	6	Mild ID, ADHD
03325	M	15	BIF
03326	M	22	Profound ID
03327	M	7	Moderate MCA/ID
03328	F	32	Mild ID, Anxiety Disorder, Neurosensory Deafness
03329	M	17	Moderate ID
03330	M	23	BIF
03331	M	11	Mild ID, Microcephaly
03332	M	21	Severe MCA/ID
03347	M	7	Mild ID, PDD
03348	F	8	BIF
03349	M	18	Moderate ID
03352	M	14	Moderate ID, Craniosynostosis, EEG anomalies
03353	M	17	Severe ID, Partial Epilepsy
03355	F	41	NOS ID, Drug-resistant Partial Epilepsy
03356	M	13	Moderate ID
03357	F	23	NOS ID, Psychosis
03358	M	8	Mild ID, PDD
03359	M	18	Severe MCA/ID
03360	M	13	Bipolar Disorder
03361	M	10	BIF
03362	F	14	Severe ID, ASD, EEG Anomalies
03363	M	7	NOS ID, ASD, EEG Anomalies
03364	M	6	NOS ID, Partial Epilepsy
03365	M	3	Epileptic Encephalopathy
03366	F	28	Profound ID, Severe Myoclonic Epilepsy
03367	F	17	Moderate ID
03368	M	10	Severe ID, VACTERL Association
03369	M	43	Severe ID

03370	F	45	NOS ID, Microcephaly	
03371	F	10	Moderate ID, Obesity, EEG anomalies	
03372	M	19	Moderate ID	
03373	F	30	Moderate ID, Psychosis	
03374	F	22	Moderate ID	
03376	M	12	Mild ID, ADHD	
03378	F	8	Severe ID, ASD	
03379	F	7	Moderate ID, PDD	
03380	F	5	Mild ID	
03381	F	12	NOS MCA/ID	
03382	M	9	BIF	
03383	M	7	PDD	
03384	M	11	BIF	
03385	M	12	NOS ID, Early Onset Psychosis	
03386	M	6	NOS ID, PDD	
03387	M	7	Moderate ID, Epilepsy, Klinefelter syndrome	Karyotype
03388	F	23	Moderate ID, Schizophrenia	
03389	M	27	Severe ID, Palpebral Myoclonus Syndrome with Absences	
03391	F	23	Severe ID, Partial Epilepsy, Spastic Tetraparesis	
03394	M	17	Moderate ID	
03396	F	19	Profound ID, PCH 2	
03397	F	16	Mild ID	
03398	M	28	Paranoid Schizophrenia	
03399	F	25	Mild ID, Anxiety Disorder	
03400	M	12	Moderate ID, ASD, Obesity	
03401	F	16	Severe MCA/ID, Epilepsy	
03402	F	6	NOS ID, PDD	
03403	M	9	BIF, Mixed Speech Impairment, EEG Anomalies	
03404	M	11	Mild ID, Early Onset Psychosis	

03405	M	40	Mild ID, Schizoid Personality Disorder
03406	F	7	BIF, Expressive Speech Disorder
03407	M	16	Mild MCA/ID
03411	M	8	Moderate ID, Epilepsy
03412	F	6	Speech Delay, EEG Anomalies
03413	M	10	NOS MCA/ID, PDD, Generalized Epilepsy
03414	M	6	NOS ID, ASD, Plagiocephaly
03415	M	12	Mild ID, Expressive Speech Disorder, Epilepsy
03419	M	13	Mild ID, ADHD, Epilepsy
03422	F	14	BIF, Anxiety Disorder
03425	F	28	Severe ID, Obesity
03434	M	16	Moderate ID
03435	F	5	NOS ID
03436	F	13	Severe ID, ASD
03437	M	14	Moderate MCA/ID
03439	M	16	Mild ID, Psychosis
03440	M	13	Moderate ID
03441	M	6	NOS ID, PDD
03442	M	20	Moderate ID
03443	M	22	Mild ID, Anxiety Disorder, Epilepsy
03444	M	16	Moderate ID, ASD, Mood Disorder
03445	M	11	Mild ID
03447	F	10	Learning Disability, Craniosynostosis
03448	F	23	Moderate MCA/ID, Obesity
03449	M	10	Mild MCA/ID
03466	M	6	Mild MCA/ID
03467	F	11	Mild ID
03468	M	16	Severe ID
03469	M	14	Severe ID

03470	M	8	NOS ID, PDD	
03471	F	14	BIF, ADHD	
03472	F	11	BIF	
03473	M	44	Normal Intellectual Functioning, Infertility	
03474	F	7	Severe ID, Microcephaly	
03475	F	5	Severe ID, Generalized Epilepsy	
03476	M	8	NOS ID, ASD, 15q11.2 deletion	
03477	M	20	Mild ID	
03478	F	8	Severe MCA/ID, Spastic Tetraparesis	
0422	F	25	Severe ID, ASD, Epilepsy	
0699	F	21	Severe ID, Epilepsy, 47,XXX	Karyotype
0701	F	23	Profound ID, Microcephaly, partial Epilepsy	
047	F	22	Moderate ID, Severe Myoclonic Epilepsy	
0626	F	56	Mild ID, Coffin-Lowry Syndrome	
0644	M	22	Mild MCA/ID	
072	M	23	Severe ID, ASD	
078	F	36	Severe MCA/ID, Ataxia, Cerebellar Hypoplasia	
0782	M	24	Profound MCA/ID, Epilepsy	
0837	M	20	Profound ID, Spastic Tetraparesis, Microcephaly	
1799	M	10	Severe ID, Brachmann De Lange syndrome	
2797	M	10	BIF, PDD	
3213	M	10	Severe ID	
3042	M	7	PDD	
3262	F	25	Severe MCA/ID, Alopecia	
2559	M	13	Profound ID, 46, XY, dup (2)(q32®q33	Karyotype
3236	F	10	Severe ID, PDD, Partial Epilepsy, Macrocephaly	PTEN gene
