# Supplementary material for: Relative Burden of Large CNVs on a Range of Neurodevelopmental Phenotypes
Source: PLoS Genet. 2011 Nov 10;7(11):e1002334. doi: 10.1371/journal.pgen.1002334 (PMC3213131; doi:10.1371/journal.pgen.1002334)
Supplement: Table S7 — Clinical features of cases with ID plus MCA. Presenting clinical features, additional malformations, and preliminary cytogenetic and genetic evaluations performed for these cases are shown. (PDF) [file pgen.1002334.s014.pdf]

Table S7. Clinical features of cases with ID plus MCA

Sample ID	Age at diagnosis	Sex	Clinical features	Malformations	Other testing
GB1	2 y	F	Goldenhar-VACTERL spectrum	bilateral anotia, bilateral choanal atresia, palatoschisis, multiple VSD, hemivertebrae	CDH 7 molecular analysis:negative
GB2	12 y	M	DD/ MR, Cohen syndrome	none	
GB3	5 y	F	MCA- DD/MR, Odho syndrome	NA	
GB4	2 months	M	MCA, Haemifacial microsomia	left atresia of external auditive duct, right microtia and low set bud ear, admedial cleft hard and soft palate, malar hypoplasia with left temporal depression, balanic hypospadia, cutaneous syndactyly of 2nd and 3th toes	not performed
GB5	6 y	M	MCA, Piebaldism	right calcaneovalgus, left talipes varus	
GB6	7 y	F	MCA, Rieger syndrome	right congenital glaucoma, left microphtalmia and anterior segment dysgenesis, cleft uvula, hypodontia and conical teeth, hyperplasia of frenulum of the tongue	not performed<br>proposed PITX2 analysis
GB7	6 months	F	MCA-DD, Rubinstein-Taybi syndrome	Patent ductus arteriosus, ASD,<br>broad thumbs	FISH 22q11 : neg<br>EP300 gene: neg<br>CREBB gene : neg
GB8	17 y	F	MCA, Turner phenotype - XX Ovarian Dysgenesis	spondylosyndesis C2-C3 and C6-C7, sacralization of L5, pectus carinatum,	karyotype onfibroblasts and ovaric tissue: 46,XX
GB9	11 months	M	MCA-DD/MR, Microphtalmia plus syndrome	left microphthalmia, right anophthalmia and orbital hypoplasia, left choanal atresia,right cleft palate, platispondilia of thoracic vertebral body, right ectopic kidney cryptorchidism, micropenis	
GB10	7 y	M	MCA-DD/MR, Seckel-like syndrome	NA	
GB11	2 y	M	MCA-DD/MR, VACTERL	left microphthalmia, right anophthalmia and orbital hypoplasia, left choana atresia,right choana stenosis,right cleft palate, maxillary fission,<br>platispondilia of thoracic vertebral body, right ectopic kidney, cryptorchidism, micropenis	DEB: negative for FANCONI Syndrome
GB12	1 y	M	MCA-DD/MR, Beckwith-Wiedemann syndrome	NA	VLCFA dosage:negative; FISH 22 and 10:negative;<br>methylation analysis 11p15: negative
GB13	6 y	M	MCA-DD/MR, Coffin-Siris syndrome	hypoplastic distal phalanges	17p microdeletion: negative

GB14	2 y	M	MCA-DD/MR, COFS syndrome	bilateral congenital cataract, VSD, bilateral pyelectasia	Analyses of DNA repair on fibroblast: NEG; metabolic analysis: negative
GB15	2 y	F	MCA-DD/MR, Goldenhar spectrum	left anotia, cheilognathopalatoschisis, hypoplastic tongue,VSD	not performed
GB16	8 y	F	MCA-DD/MR, Hemifacial macrosomia	left external auditory duct atresia, left poliotia, external right ear malformation, right epibulbar dermoid cyst, ASD and VSD, transposition of the great vessels	FISH 22q11.2 negative
GB17	5 y	F	MCA-DD/MR, Hypomelanosis of Ito	camptodactyly of 5th finger	
GB18	4 months	F	MCA-DD/MR, Mowat-Wilson syndrome	Hirschprung disease, Patent doctus arteriousus	FISH 22q11.2 negative
GB19	15 y	M	MCA-DD/MR, Myhre syndrome	agenesia of 5 teeth,conic teeth,cleft palate,bilateral cryptorchidism,agenesia of deferent,brachydactylia	
GB20	4 months	M	MCA, Blepharophymosis-epicanthus inversus	none	
GB21	14 months	M	MCA-DD/MR, Overgrowth syndrome, Cranio-fronto-nasal syndrome spectrum	diastasis recti abdominis, cryptorchidism, hypospadia	NSD,FGFR2,FGFR3,TWIST, EPHRIN B, EFNB analysis: negative; del5q35.3:negative,
GB22	3 days old	F	MCA, Seckel like phenotype	cleft palate, arthrogryposis	
GB23	4 y	F	MCA-DD/MR, Sotos like phenotype	blue scleras	FRAXA:negative; FISH 5q35.3:negative; NSD1 analysis:negative; molecular analysis for Prader-Willi syndrome, Steinert syndrome: negative. Muscular biopsy: negative.
GB24	11 y	M	MCA-DD/MR, Joubert syndrome suspicion	NA	
GB25	6 months	F	MCA-DD/MR, Aicardì syndrome	pseudocoloboma iris	
GB26	5 y	M	MCA-DD/MR, Pallister-Killian Syndrome suspicion	VSD	
GB27	1 month	F	MCA-DD/MR, WAGR/Beckwith-Wiedemann syndrome	congenital cataract, left megaureter, right pyelectasia	metabolic analysis:negative; 11p15 methylation pattern negative
GB28	7 months	M	BECKWITH	lower limb's dysmetria,	11p15 methylation pattern negative

			WIEDEMANN SYNDROME	umbelical hernia,auricular pits	
GB29	1 month	M	Susp Menkes,susp Cutis Laxa syndrome	none	connexina 26 neg, MENKES negative,<br>cystic fibrosis negative
GB30	8 y	F	DD/MR, Cornelia de Lange spectrum	NA	NIPLB 1 analysis: in progress
GB31	17 y	F	DD/MR, Beckwith Wiedemann	asymmetric lower extremities,<br>mandibulofacial dysostosis	11p15 methylation pattern negative
GB32	8 y	F	DD/MR,Dubowitz Syndrome	NA	
GB33	3 months	M	MCA, Lung hypoplasia syndrome	dextrocardia, ASD,<br>right lung hypoplasia	not performed
GB34	2 days	M	SPECIFIC SYNDROMIC PHENOTYPE<br>MCA<br>VACTERL association	cleft palate, left upper extremity hemimelia, left hand agenesia, T3 butterfly vertebra, 5th rib arch anomaly, right kidney hypoplasia and ectopia, left kidney malrotation imperforate anus	not performed
GB35	11 y	F	OTHER, CNS abnormalities	none	metabolic analysis:negative
GB36	11 y	F	OTHER, familial CHD	bilateral cleft palate,<br>aortic subvalvular stenosis	
GB37	3 y	M	OTHER, familial CHD	patent ductus arteriosus,bilateral crytorchidism	not performed
GB38	6 months	M	Syndromic CHD	tetralogy of Fallot, pulmonary valve agenesis, posteriorly placed anonymous vein, short humerus	
GB39	4 y	F	CHD	myocardiopathy and<br>Wolff-Parkinson-White syndrome	
GB40	6 y	F	Facial chromosomal phenotype	NA	
GB41	4 months	M	Isolated hemihypertrophy	NA	normal metilation pattern of KvDMR1 and H19 (BWS negative)
GB42	10 y	F	MCA DD/MR, Mulwihill-Smith Syndrome	ASD	analysis of gene laminin: normal; serphin gene not deleted
GB43	11 y	F	MCA- DD/MR, microcephaly-lymphedema syndrome	S1 schysis	FISH 22q11 and FISH10p:negative
GB44	1 month	M	MCA-DD, COFS spectrum	II-III toes syndactyly	metabolic analysis:negative

GB45	1 day	M	DD/MR, De Lange spectrum	unilaterla preaxial polidactyly	
GB46	11 y	M	DD/MR, CHARGE syndrome	choanal stenosis,short neck, carenate breastbone,pyelic ectasia	
GB47	2 y	F	DD/MR, DiGeorge syndrome-like phenotype	truncus	FISH22q11:negative deletion 22q11 on amniocytes(microsatellites)
GB48	2 months	M	DD/MR, 8 Trisomy mosaic	patent ductus arteriosus,mild septal hypertrophy, hypospadia, right cryptorchidism,right pyelectasia	Karyotype on 50 metaphases:47,XY,+8 (84%)/46,XY
GB49	18 y	M	MCA-DD/MR, Beals Syndrome	cataract, camptodactyly, severe scoliosis, joint contractures, left cryptorchidism	
GB50	8 y	F	MCA-DD, Beckwith-Wiedemann-like phenotype	NA	
GB51	5 months	F	Developmental delay	low set ears, hypertelorism, long philtrum	
GB52	9 months	M	Beckwith-Wiedemann	NA	metabolic analysis:negative; 11p15 methylation pattern negative
GB53	NA.	M	Noonan-like phenotype	multiple VSD, cryptorchidism, short stature	PTPN11 and SOS1 neg
GB54	NA.	F	Noonan-like phenotype	pulmonic valve stenosis	PTPN11 negative
GB55	NA.	M	Noonan-like phenotype	severe hypertrophic cardiomyopathy	PTPN11 negative
GB56	NA.	F	Noonan-like phenotype	pulmonic valve stenosis mitral insufficiency	PTPN11 negative SOS1 negative
GB57	NA.	M	Noonan-like phenotype	NA	PTPN11 negative
GB58	NA.	M	Noonan-like phenotype	VSD, cryptorchidism,	PTPN11 negative SOS1 negative
GB59	NA.	M	Noonan-like phenotype	arrhythmia cryptorchidism	PTPN11 negative SOS1 positve
GB60	NA.	M	Noonan-like phenotype	pulmonic valve stenosis, cryptorchidism	PTPN11 negative
GB61	NA.	M	Noonan-like phenotype	pulmonic valve stenosis	PTPN11 negative
GB62	NA.	M	Noonan-like phenotype	tetralogy of Fallot, cryptorchidism	PTPN11 negative
GB63	NA.	F	Noonan-like phenotype	pulmonic valve	PTPN11

				stenosis	negative
GB64	NA.	M	Noonan-like phenotype	hypertrophic cardiomyopathy	PTPN11 negative
GB65	NA.	F	Noonan-like phenotype	pulminc valve stenosis	PTPN11 negative
GB66	NA.	M	Noonan-like phenotype	double outlet right ventricle	PTPN11 negative
GB67	5 days	M	MCA/VATER association	right coloboma of the retina,right posterior polar cataract, right microphthalmia,D-T butterfly vertebras, XII rib agenesia	
GB68	9 y	M	MCA-DD/MR, Cornelia de Lange syndrome	atrial sept defect (ostium primum and ostium secundum), mitralic valve incompetence, crytorchidism,clinodactyly 5th finger	NIPLB 1 analysis:negative
GB71	17 y	F	MCA-DD/MR. 9 Trisomy	congenital dislocation of the hip	
GB72	1 month	F	MCA, Mowat-Wilson	Hirschprung's disease	
GB73	1 month	F	MCA-DD/MR, Mowat-Wilson	Hirschprung's disease	RET:negative ZFHX1B:negative
GB88	NA.	M	MCA, 1 Trisomy	polycistic kidney, duodenum stenosis,bowel malrotation,pancreas malformation,staphyloschisis	
GB92	NA.	M	Williams syndrome	anomalous pulmonary venous return, long SQT interval (pacemaker), polmunary regurgitation	Del7q21.23
