# Supplementary material for: Relative Burden of Large CNVs on a Range of Neurodevelopmental Phenotypes
Source: PLoS Genet. 2011 Nov 10;7(11):e1002334. doi: 10.1371/journal.pgen.1002334 (PMC3213131; doi:10.1371/journal.pgen.1002334)
Supplement: Table S8 — (A) Comparison of rare CNV rates in the cohorts studied. (B) Rare CNVs in dyslexia, autism, and ID. (C) Inheritance of rare CNVs in the disease cohorts. (D) Individuals with two rare copy number variants (two hits). (PDF) [file pgen.1002334.s015.pdf]

Table S8A. Comparison of rare CNV rates in the cohorts studied

Cohort1	Cohort2	No. of rare CNV cases (cohort1)	No. of rare CNV cases (cohort2)	Significance (p-value)	Odds ratio
IDMCACombined	Dyslexia	69	6	3.55E-12	10.016404
IDMCACombined	Autism	69	35	0.018974303	1.6110072
Autism	Dyslexia	35	6	2.41E-06	6.1095819
ID	MCA	60	9	0.225328002	1.4306148
AutismMR	AutismNoMR	11	25	0.476515456	1.0922966
Dyslexia	NIMH control	6	6	0.578710884	0.9494
ID	NIMH control	60	6	1.09E-11	10.0671
Autism	NIMH control	35	6	5.06702E-06	5.814
IDMCACombined	NIMH control	69	6	1.43E-11	9.5304

Table S8B. Rare CNVs in dyslexia, autism, and ID

Cohort	Total	Cases	CNVs	Two hits	RefSeq genes	median size	Hotspot CNVs	non-hotspot CNVs	genomic disorder
Dyslexia	322	6 (1.9%)	6 (2%)	0 (0%)	10	302 kb	3 (50%)	3	0
Autism	336	35 (10.4%)	36 (10.7%)	1 (2.8%)	680	662 kb	21 (58%)	15	8
ID	358	60 (16.8%)	64 (17.9%)	4 (6.6%)	1537	849 kb	20 (31%)	44	15
ID/MCA	73	9 (12.3%)	13 (17.8%)	4 (44%)	678	1.86 Mb	5 (60%)	8	5

Table S8C. Inheritance of rare CNVs in the disease cohorts

	**Total tested**	***de novo***	**Paternal**	**Maternal**	**Total inherited**	**Both**	**Complex***
Dyslexia	8	0	5	3	8 (100%)		
Autism	35	14 (40%)	9	11	21 (60%)	1	
ID	47	30 (63.8%)	5	9	14 (29.8%)		3

*Complex inheritance includes balanced translocations.

Table S8D. Individuals with two rare copy number variants

**Cohort**	**Sample**	**Chrom**	**Start**	**Size**	**Chr. Band**	**CNV**	**inheritance**

ID	699	chr4	152,300,259	423,718	4q31.3	deletion	de novo
		chrY	3,072,083	3,082,442	Yp11.2	duplication	de novo
ID	2522	chr15	80,767,738	19,379,303	15q25	duplication	paternal or de novo
		chr2	188,179,827	673,252	2q32.1	duplication	paternal or de novo
ID	2597	chr11	121,813,520	12,633,728	11q24.1-q25	deletion	de novo
		chrX	65,611,216	322,784	Xq12	deletion	maternal
ID	3448	chr3	50,846,910	7,577,247	3p21.31p14.3	duplication	de novo
		chr5	156,526,018	7,607,806	5q33.3q34	duplication	de novo
ID with MCA	GB58	chr3	197,391,142	2,039,784	3q29	duplication	de novo
		chr17	3,707	1,856,379	17p13.3	deletion	de novo
ID with MCA	GB6	chr4	110,560,125	3,335,124	4q25	deletion	de novo
		chr16	15,170,619	3,491,890	16p13.11	duplication	paternal
ID with MCA	GB71	chr9	201336	16,470,976	partial trisomy 9	duplication	46,XX rcp (8;10)(q2.2;q21.2) + t (9;12)(p2.2;p1.3)
		chr13	83,679,489	705,821	13q31.1 dup	duplication	maternal
ID with MCA	GB88	chr5	90,252	1,540,511	5p15.33	deletion	46,XX,t(1;5)(q23;p15) balanced (maternal)
		chr1	174,979,260	63,278,601	1q24qter	duplication	46,XX,t(1;5)(q23;p15) balanced (maternal)
Autism with ID	Si84	chr16	29,546,342	583,059	16p11.2	deletion	de novo
		chr22	21,298,606	2,064,156	22q11.22	duplication	maternal

Shaded rows depict two hits due to a derivative chromosome representing a single unbalanced translocation event
