# Supplementary material for: Relative Burden of Large CNVs on a Range of Neurodevelopmental Phenotypes
Source: PLoS Genet. 2011 Nov 10;7(11):e1002334. doi: 10.1371/journal.pgen.1002334 (PMC3213131; doi:10.1371/journal.pgen.1002334)
Supplement: Table S9 — Genomic disorders identified in 1,227 cases with ID, autism, and ID/MCA. Genomic disorders are defined as copy number variants that are previously identified to be associated significantly in individuals with disease compared to controls. These CNVs can either map within genomic hotspot (HS) or non-hotspot regions (non-HS). (PDF) [file pgen.1002334.s016.pdf]

Table S9. Genomic disorders identified in 1,227 cases with dyslexia, autism, ID, and ID/MCA.

Chr	Start	End	Size	Chr. Band	CNV	Sample	Cohort	Type	Inheritance	Control CNVs	Gene count
chr1	743498	5334310	4590812	1p36	deletion	3332	ID	non-HS	de novo	0	91
chr1	245415305	247043877	1628572	1q44	deletion	Si138	Autism	non-HS	maternal	0	48
chr15	18045749	30589746	12543997	15q11.2q13.3	duplication	3213	ID	HS	de novo	0	142
chr15	26994610	30522495	3527885	15q13.1q13.3	deletion	1879	ID	HS	de novo	0	17
chr16	14936606	16426815	1490209	16p13.12	deletion	3401	ID	HS	maternal	2	15
chr16	15170619	18662509	3491890	16p13.11	duplication	GB6	ID/MCA	HS	paternal	2	14
chr16	15367033	18321829	2954796	16p11.31p12.3	deletion	3376	ID	HS	maternal	0	12
chr16	15386338	16177142	790804	16p13.12	duplication	2449	ID	HS	maternal	10	9
chr16	28730254	28949347	219093	16p11.2	deletion	3360	ID	HS	NA	1	9
chr16	29554938	30104150	549212	16p11.2	deletion	2257	ID	HS	NA	3	27
chr16	29655898	29979351	323453	16p11.2	deletion	3042	ID	HS	NA	3	18
chr17	3707	1860086	1856379	17p13.3	deletion	GB58	ID/MCA	non-HS	de novo	0	31
chr17	13958086	15457720	1499634	17p12	duplication	Si102	Autism	HS	de novo	2	9
chr17	31880822	33305362	1424540	17q12	deletion	3299	ID	HS	de novo	2	18
chr17	41063431	41533855	470424	17q21.31	deletion	GB61	ID/MCA	HS	de novo	0	9
chr22	46841144	49542685	2701541	22q13.22	deletion	3282	ID	non-HS	Paternal/de novo	0	41
chr3	197188186	198861094	1672908	3q29	deletion	3249	ID	HS	de novo	0	24
chr3	197391142	199430926	2039784	3q29	duplication	GB58	ID/MCA	HS	de novo	0	33
chr5	175479594	177400099	1920505	5q35	deletion	3359	ID	HS	de novo	0	41
chr6	100383567	103310184	2926617	6q16.3	deletion	1755	ID	non-HS	de novo	0	4
chr7	72300576	73798218	1497642	7q11.23	deletion	GB92	ID/MCA	HS	de novo	0	28
chr8	8085497	11899019	3813522	8p23.1	deletion	2473	ID	HS	NA	0	32
chr15	18193472	26871283	8677811	15q11.2q13.1	duplication	Si41	Autism	HS	de novo	0	125
chr16	29546342	30129401	583059	16p11.2	deletion	Si175	Autism	HS	de novo	3	36
chr16	29546342	30129401	583059	16p11.2	deletion	Si245	Autism	HS	de novo	3	36
chr16	29546342	30129401	583059	16p11.2	deletion	Si84	Autism	HS	de novo	3	36
chr22	17266528	19800553	2534025	22q11.21	deletion	Si123	Autism	HS	de novo	0	59
chr22	21298606	23362762	2064156	22q11.22	duplication	Si84	Autism	HS	maternal	3	41
chr15	20301665	20602651	300986	15q11.2	deletion	937	Dyslexia	HS	paternal	25	7
