# Supplementary material for: Relative Burden of Large CNVs on a Range of Neurodevelopmental Phenotypes
Source: PLoS Genet. 2011 Nov 10;7(11):e1002334. doi: 10.1371/journal.pgen.1002334 (PMC3213131; doi:10.1371/journal.pgen.1002334)
Supplement: Table S10 — Clinical features of a case with 6q16 deletion. Comparison of clinical features from published sources with those in the current study. (PDF) [file pgen.1002334.s017.pdf]

Table S10. Clinical features of an individual with 6q16 deletion.

**Clinical features**	Previous studies*	This study
Age of the patient	NA	10 year old male
Inheritance	NA	de novo
Developmental delay	10/10	mild mental retardation
Language delay	6/10	No
Hyperphagia	5/10	No
Hypotonia	8/10	No
Obesity	7/10	No
Tapered fingers	2/10	No
Clinodactyly	2/10	No
Short stature	2/10	No
Vision anomalies	7/10	Hypermetropic astigmatism
Behavioral problems (autism features)	6/10	No
Cardiac features	2/10	No
Brain anomalies	5/10	No
EEG anomalies	4/10	No
Rounded face/full cheeks	4/10	asymmetric facies
Skull anomalies	7/10	craniosynostosis (plagiocephaly Left posterior and Right anterior
Microcephaly	1/10	No
Eye anomalies	5/10	No
Low-set ears	4/10	No
malformed ears	9/10	Asymmetry
Nose	9/10	No
Philtrum	3/10	smooth philtrum
Microretrognathia	7/10	No
Mouth/malpositioned teeth	2/10	narrow mouth
High arched palate	2/10	No
Skeletal features	NA	joint laxity, pes planus, partial 2nd-3rd toe cutaneous syndactyly
Perinatal history		Fracture on the right clavicle during eutocic delivery after 38 weeks of pregnancy
Birth weight	NA	3,150 grams
Birth length	NA	NA
Head circumference (OFC)	NA	34 cm
Maternal age	NA	NA
Paternal age	NA	NA

*Previous study includes Bonaglia et al., 2008[2]; Klein et al., 2007[3]; Le Caignec et al., 2005[4]; and Verela et al., 2006[5].
