# Supplementary material for: Relative Burden of Large CNVs on a Range of Neurodevelopmental Phenotypes
Source: PLoS Genet. 2011 Nov 10;7(11):e1002334. doi: 10.1371/journal.pgen.1002334 (PMC3213131; doi:10.1371/journal.pgen.1002334)
Supplement: Figure S2 — Comparison of 9p24 deletions identified in our study to DECIPHER database. The figure shows genome browser snapshot of the 9p24 region with red and blue bars denoting deletions and duplications respectively in the DECIPHER database. Major clinical features observed in these patients are also shown. Please note that the black bars at the top denote CNVs identified in our study. PDD-NOS, Pervasive developmental delay-not otherwise specified; ID/DD, intellectual disability/developmental delay. (PDF) [file pgen.1002334.s006.pdf]

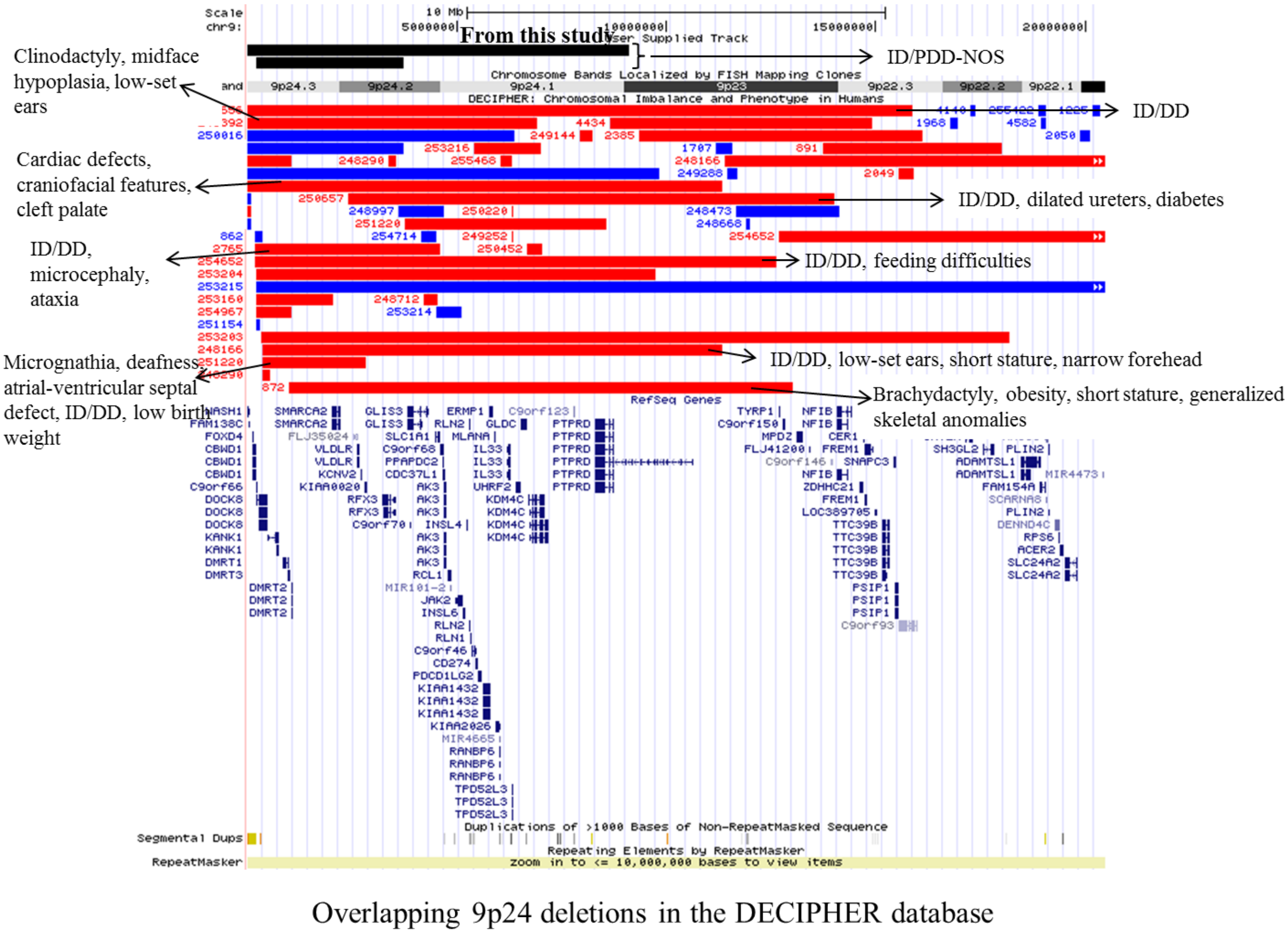

Figure S2. Comparison of 9p24 deletions identified in our study to those reported in the DECIPHER database
